# Supplementary material for: The Atypical MAP Kinase ErkB Transmits Distinct Chemotactic Signals through a Core Signaling Module
Source: Dev Cell. 2019 Feb 25;48(4):491–505.e9. doi: 10.1016/j.devcel.2018.12.001 (PMC6397043; doi:10.1016/j.devcel.2018.12.001)
Supplement: Document S1. Figures S1–S7 and Tables S1 and S2 [file mmc1.pdf]

Developmental Cell, Volume *48*

Supplemental Information

The Atypical MAP Kinase ErkB Transmits Distinct Chemotactic Signals through a Core Signaling Module

John M.E. Nichols, Peggy Paschke, Sew Peak-Chew, Thomas D. Williams, Luke Tweedy, Mark Skehel, Elaine Stephens, Jonathan R. Chubb, and Robert R. Kay

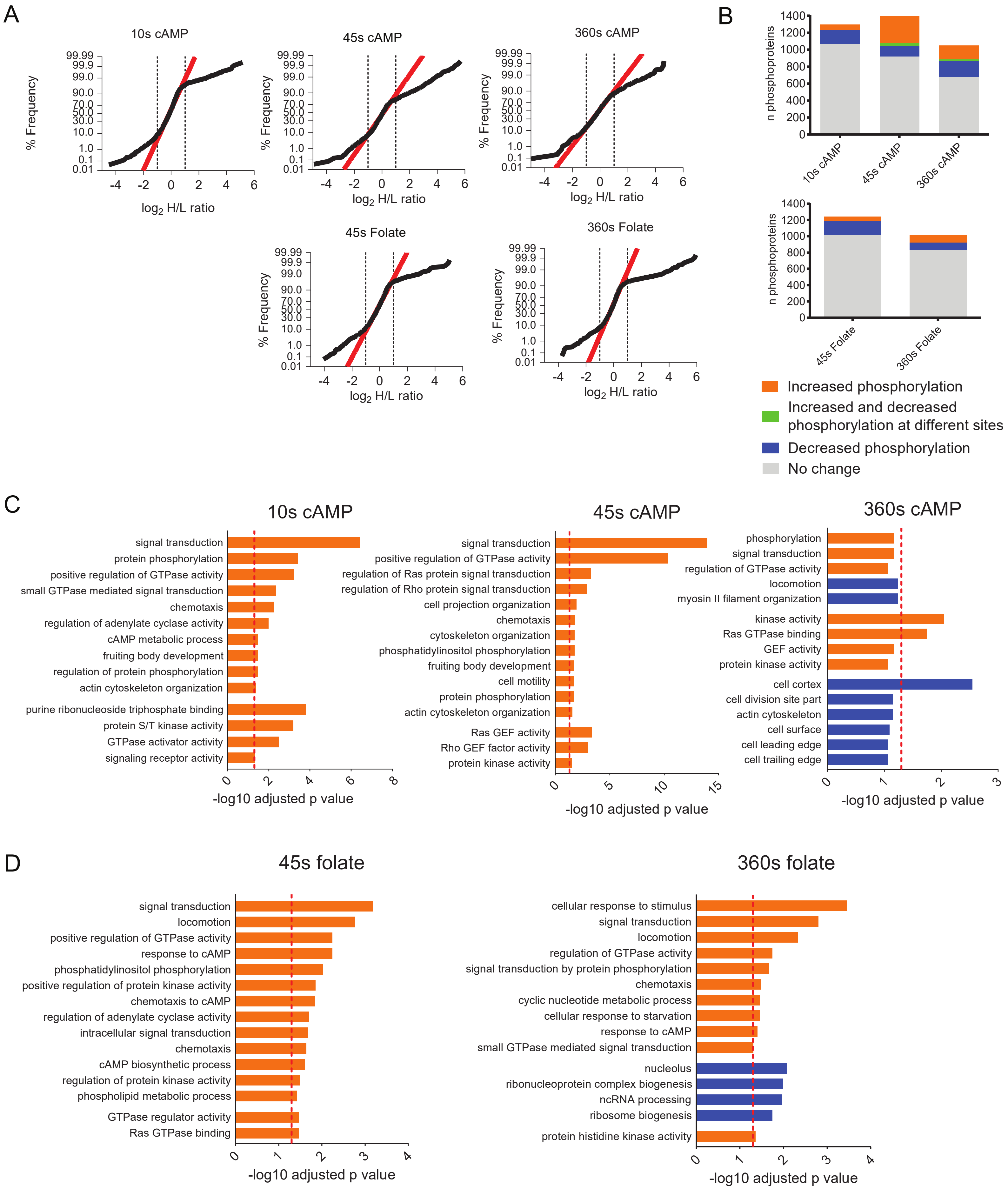

A
10s cAMP
45s cAMP
360s cAMP
45s Folate
360s Folate
% Frequency
log2 H/L ratio
B
n phosphoproteins
10s cAMP
45s cAMP
360s cAMP
45s Folate
360s Folate
Increased phosphorylation
Increased and decreased phosphorylation at different sites
Decreased phosphorylation
No change
C
10s cAMP
signal transduction
protein phosphorylation
positive regulation of GTPase activity
small GTPase mediated signal transduction
chemotaxis
regulation of adenylate cyclase activity
cAMP metabolic process
fruiting body development
regulation of protein phosphorylation
actin cytoskeleton organization
purine ribonucleoside triphosphate binding
protein S/T kinase activity
GTPase activator activity
signaling receptor activity
-log10 adjusted p value
45s cAMP
signal transduction
positive regulation of GTPase activity
regulation of Ras protein signal transduction
regulation of Rho protein signal transduction
cell projection organization
chemotaxis
cytoskeleton organization
phosphatidylinositol phosphorylation
fruiting body development
cell motility
protein phosphorylation
actin cytoskeleton organization
Ras GEF activity
Rho GEF factor activity
protein kinase activity
-log10 adjusted p value
360s cAMP
phosphorylation
signal transduction
regulation of GTPase activity
locomotion
myosin II filament organization
kinase activity
Ras GTPase binding
GEF activity
protein kinase activity
cell cortex
cell division site part
actin cytoskeleton
cell surface
cell leading edge
cell trailing edge
-log10 adjusted p value
D
45s folate
signal transduction
locomotion
positive regulation of GTPase activity
response to cAMP
phosphatidylinositol phosphorylation
positive regulation of protein kinase activity
chemotaxis to cAMP
regulation of adenylate cyclase activity
intracellular signal transduction
chemotaxis
cAMP biosynthetic process
regulation of protein kinase activity
phospholipid metabolic process
GTPase regulator activity
Ras GTPase binding
-log10 adjusted p value
360s folate
cellular response to stimulus
signal transduction
locomotion
regulation of GTPase activity
signal transduction by protein phosphorylation
chemotaxis
cyclic nucleotide metabolic process
cellular response to starvation
response to cAMP
small GTPase mediated signal transduction
nucleolus
ribonucleoprotein complex biogenesis
ncRNA processing
ribosome biogenesis
protein histidine kinase activity
-log10 adjusted p value

Figure S1. Phosphorylation changes in response to cAMP and folate treatment. Related to Figure 1.

(A) Cumulative frequency plots showing distributions of SILAC ratios for cAMP and folate phosphoproteomic experiments. Red line shows Gaussian distributions fitted to the data for each experiment. Dashed lines show 2-fold SILAC ratio threshold used to define phosphorylation changes.
(B) Numbers of phosphoproteins with phosphorylation sites detected in each cAMP and folate experiment.
(C) and (D) GO analysis of phosphorylation changes following cAMP treatments (C) and folate treatments (D). Orange bars denote enrichment among sites of increased phosphorylation. Blue bars denote enrichment among sites with decreased phosphorylation. Dashed line indicates p=0.05. Reported p-values corrected for multiple comparisons using the Benjamini-Hochberg method.

A

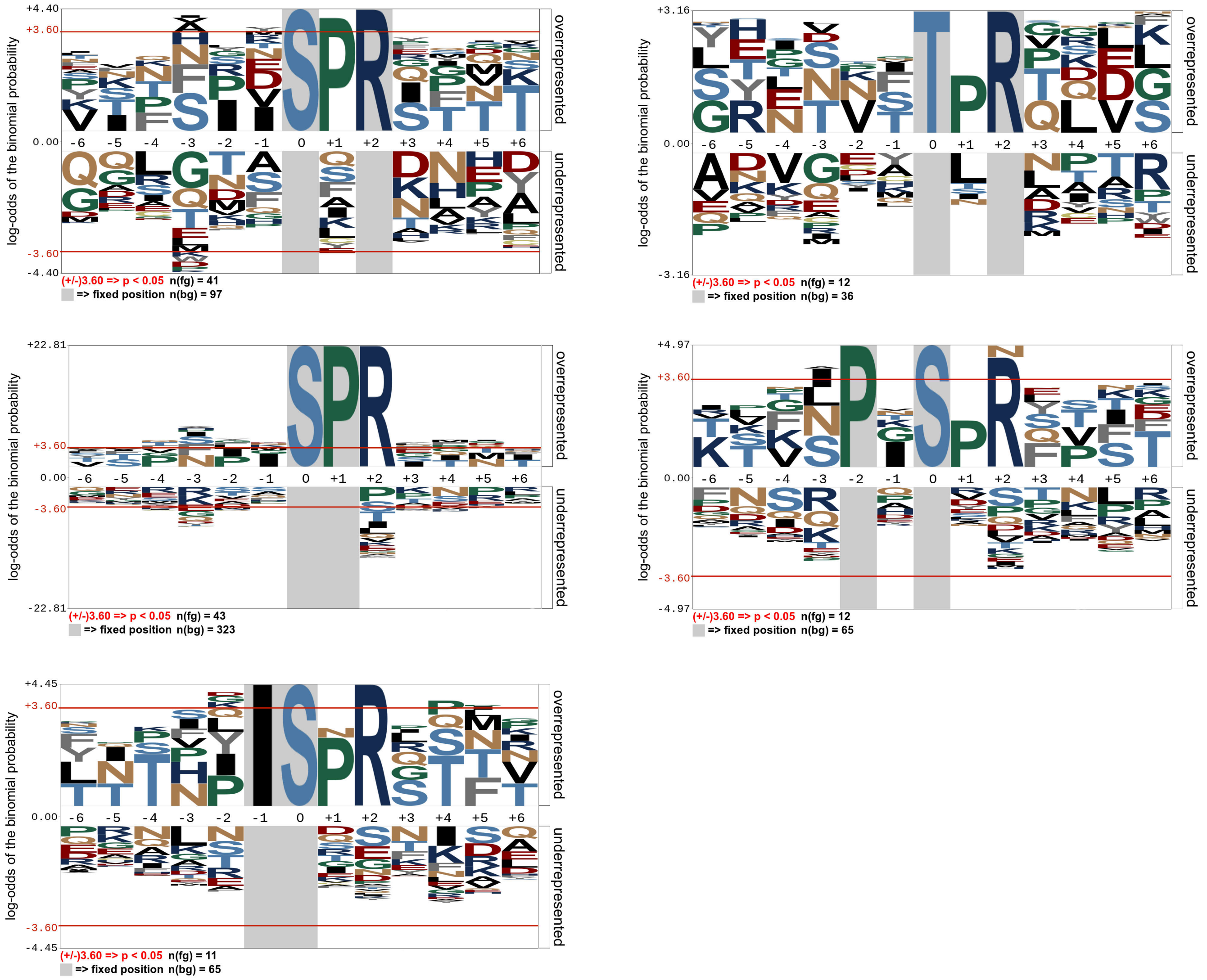

B

Increased phosphorylation

motif	10s cAMP	45s cAMP	360s cAMP	45s folate	360s folate
[S/T]PR	1	$6.8x10^{-9}$	$5.3x10^{-27}$	$1.2x10^{-36}$	$3.03x10^{-77}$
PKB	$2.7x10^{-8}$	$6.0x10^{-10}$	$4.8x10^{-3}$	0.06	1
PKA	0.01	$3.1x10^{-8}$	$4.8x10^{-9}$	1	0.04
CAMK	$5.1x10^{-4}$	$1.1x10^{-12}$	1	1	1
MAPK	1	1	0.07	$2.3x10^{-6}$	$1.25x10^{-5}$
CK1	1	0.01	0.29	0.86	0.43

Decreased phosphorylation

motif	10s cAMP	45s cAMP	360s cAMP	45s folate	360s folate
CK2	$4.2x10^{-32}$	$2.0x10^{-56}$	$3.8x10^{-27}$	$1.3x10^{-51}$	$1.3x10^{-63}$

C

Name	Sequence
[S/T]PR	p[ST]PR
Proline directed	p[ST]P
MAPK	Pxp[ST]
CDK	p[ST]Px[KR]
GSK	p[ST]xxx[ST]
CK2	p[ST][DE]x[DE]
PKA	RRxp[ST]Φ
PKB	RxRxxp[ST]
Pak	[RK]R[RK]p[ST]
CAMK	Rxxp[ST]
Nek	[FL]xxp[ST]
CK1	p[ST]xx[ST]

Figure S2. Identification of novel and known kinase consensus motifs in phosphoproteomic data. Related to Figure 2.

(A) Extended sequence logos, generated using pLogo, showing coincidence of residues making up the p[S/T]PR motif. From the base logos shown in Figure 2, a statistically overrepresented residue can be 'fixed' and used as the basis for set reduction to investigate the conditional probabilities of correlations between residues; allowing extended logos to be built. For both phosphoserine and phosphothreonine sites, when statistically significant residues from the base logo are fixed (grey box), it is observed that proline at +1 and arginine at +2 are both consistently overrepresented in the extended logos, suggesting close association of these residues in cAMP and folate regulated phosphorylation sites.

(B) Results of statistical overrepresentation of different kinase consensus motifs amongst phosphorylation sites with chemoattractant responses. Overrepresentation was tested using Fisher's exact test and p-values are adjusted using the Benjamini-Hochberg method.

(C) Kinase substrate motif consensus sequences used for classification of phosphorylation sites in Figure 2B.

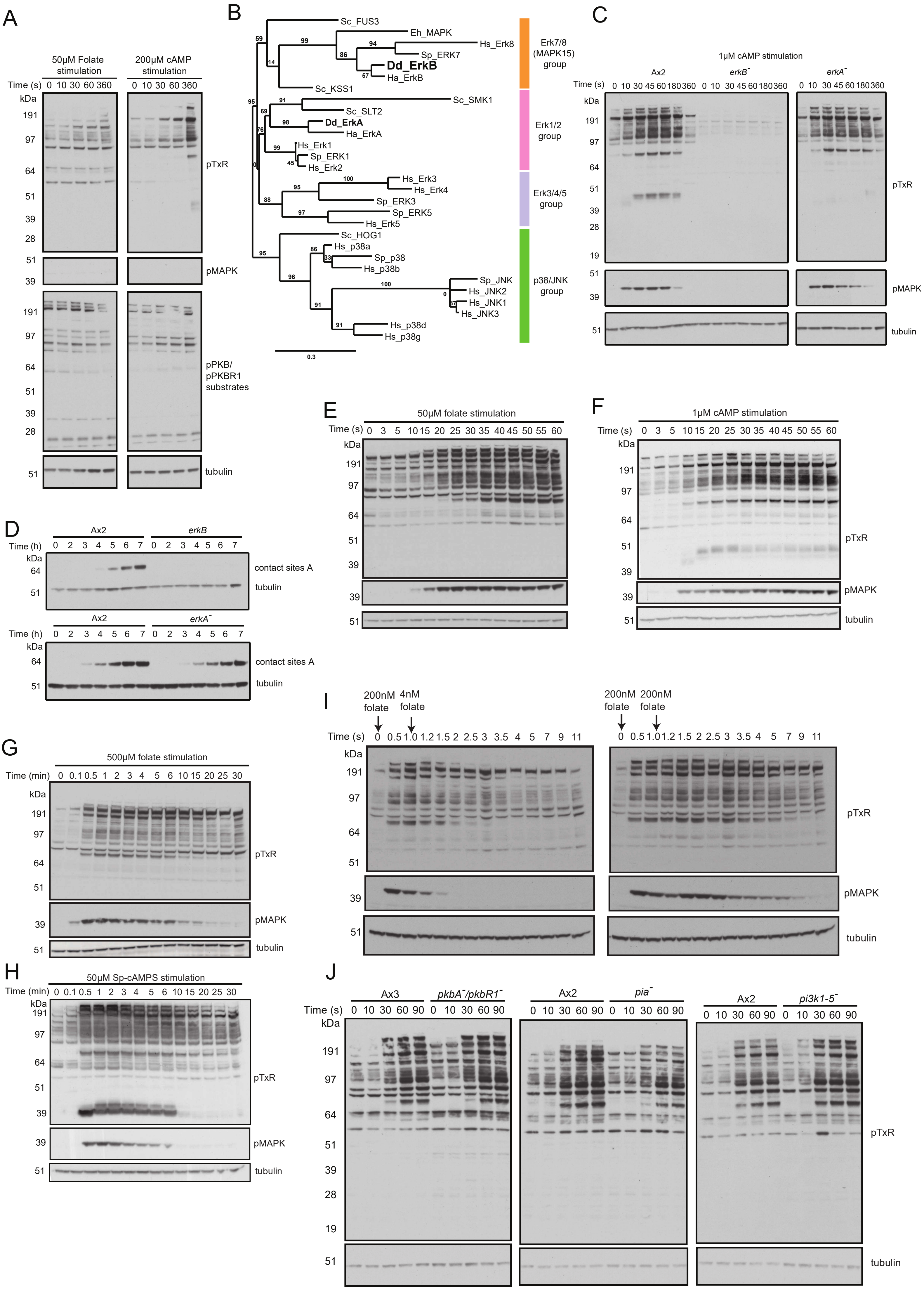
A
50µM Folate stimulation
200µM cAMP stimulation
Time (s) 0 10 30 60 360
kDa
191
97
64
51
39
28
pTxR
pMAPK
pPKB/
pPKBR1
substrates
tubulin
B
Sc_FUS3
Eh_MAPK
Hs_Erk8
Sp_ERK7
Dd_ErkB
Ha_ErkB
Sc_KSS1
Sc_SMK1
Sc_SLT2
Dd_ErkA
Ha_ErkA
Hs_Erk1
Sp_ERK1
Hs_Erk2
Hs_Erk3
Hs_Erk4
Sp_ERK3
Sp_ERK5
Hs_Erk5
Sc_HOG1
Hs_p38a
Sp_p38
Hs_p38b
Sp_JNK
Hs_JNK2
Hs_JNK1
Hs_JNK3
Hs_p38d
Hs_p38g
0.3
Erk7/8 (MAPK15) group
Erk1/2 group
Erk3/4/5 group
p38/JNK group
C
1µM cAMP stimulation
Ax2
erkB⁻
erkA⁻
Time (s) 0 10 30 45 60 180 360
kDa
191
97
64
51
39
28
19
pTxR
pMAPK
tubulin
D
Ax2
erkB
erkA⁻
Time (h) 0 2 3 4 5 6 7
kDa
64
51
contact sites A
tubulin
E
50µM folate stimulation
Time (s) 0 3 5 10 15 20 25 30 35 40 45 50 55 60
kDa
191
97
64
51
39
F
1µM cAMP stimulation
Time (s) 0 3 5 10 15 20 25 30 35 40 45 50 55 60
pTxR
pMAPK
tubulin
G
500µM folate stimulation
Time (min) 0 0.1 0.5 1 2 3 4 5 6 10 15 20 25 30
pTxR
pMAPK
tubulin
H
50µM Sp-cAMPS stimulation
Time (min) 0 0.1 0.5 1 2 3 4 5 6 10 15 20 25 30
pTxR
pMAPK
tubulin
I
200nM folate
4nM folate
200nM folate
200nM folate
Time (s) 0 0.5 1.0 1.2 1.5 2 2.5 3 3.5 4 5 7 9 11
pTxR
pMAPK
tubulin
J
Ax3
pkbA⁻/pkbR1⁻
Ax2
pia⁻
Ax2
pi3k1-5⁻
Time (s) 0 10 30 60 90
kDa
191
97
64
51
39
28
19
pTxR
tubulin

Figure S3. Investigating pTxR motif and pMAPK phosphorylation by immunoblotting. Related to Figure 3.

(A) Buffer control immunoblots for folate and cAMP treatments shown in Figure 3A. Phosphorylation in 360 s cAMP control sample likely to be caused by endogenous cAMP release from aggregation-competent amoebae.

(B) Maximum likelihood tree of *D. discoideum* ErkA and ErkB and MAPKs from other selected species: *Homo sapiens* (Hs), sea urchin (*Strongylocentrotus purpuratus*, Sp), *Saccharomyces cerevisiae* (Sc), *Dictyostelium discoideum* (Dd), *Heterostelium album* (Ha), *Entamoeba histolytica* (Ea). *Dictyostelium* ErkB aligns with metazoan Erk7/ERk8/MAPK15, while *Dictyostelium* ErkA is grouped with metazoan Erk1/2. Sequences were aligned using MUSCLE (v3.8.31). Ambiguous regions were removed using Gblocks (v0.91b), resulting in an alignment focused on the kinase domain of the proteins. Tree construction used the maximum likelihood method in PhyML and was rendered using TreeDyn.

(C) Immunoblotting of pTxR phosphorylation and pMAPK following 1 µM cAMP treatment of cAMP-pulsed wild-type, *erkB*$^-$ and *erkA*$^-$ amoebae. Cells were pulsed at 6 minute intervals with 90 nM cAMP for 5 hours before being washed and stimulated with 1 µM cAMP. Phosphorylation responses are seen in wild-type and *erkA*$^-$ cells but not observed in *erkB*$^-$ cells. Tubulin shown as a loading control.

(D) Immunoblots showing expression of the developmental marker CsaA following cAMP pulsing treatment of different durations. In wild-type and *erkA*$^-$ cells, expression of CsaA is observed after 3 hours of pulsing with cAMP. In *erkB*$^-$ cells, CsaA expression is not observed even after 7 hours of cAMP pulsing treatment. Tubulin shown as a loading control.

(E) Immunoblot showing onset of pTxR motif phosphorylation and ErkB phosphorylation (pMAPK) at high temporal resolution following stimulation of undifferentiated amoebae with 50 µM folate. Tubulin shown as loading control. Quantification is shown in Figure 3E. Representative of 2 independent experiments.

(F) Immunoblot showing onset of pTxR motif phosphorylation and ErkB phosphorylation (pMAPK) at high temporal resolution following stimulation of cAMP-pulsed amoebae with 1 µM cAMP. Tubulin shown as loading control. Quantification is shown in Figure 3E. Representative of 2 independent experiments.

(G) Immunoblot showing duration of pTxR motif phosphorylation and ErkB phosphorylation (pMAPK) during continuous saturating stimulation of undifferentiated amoebae with 500 µM folate. Tubulin shown as loading control. Quantification is shown in Figure 3F. Representative of 2 independent experiments.

(H) Immunoblot showing duration of pTxR motif phosphorylation and ErkB phosphorylation (pMAPK) during continuous saturating stimulation of cAMP-pulsed *acaA*$^-$ amoebae with 50 µM Sp-cAMPS. Tubulin shown as loading control. Quantification is shown in Figure 3F. Representative of 2 independent experiments.

(I) Immunoblot showing dephosphorylation of pTxR motif phosphoproteins and of ErkB following stimulation of wild-type undifferentiated amoebae with 200 nM folate and subsequent dilution after 60 s in buffer to reduce folate concentration to 4 nM. Dilution of cells to the same extent with buffer containing 200 nM folate leads to continued phosphorylation. Tubulin shown as loading control. Blots shown are representative of two independent experiments. Quantification shown in Figure 3G.

(J) pTxR phosphorylation response (upper panels) to 50 µM folate treatment in chemotaxis signalling mutants and parent wild-type strains. The pTxR response is intact in knockout cells lacking PKB and PKBR1, cells lacking the TORC2 component Pianissimo, and cells lacking PI3K1-5. Similarly, the response is intact in knockouts of the PI5K PikI, RasC/G (singly and double), GcA/SgaA double, SCAR, PLA2, PKAcat, and IPLA (data not shown). Lower panels show tubulin as a loading control.

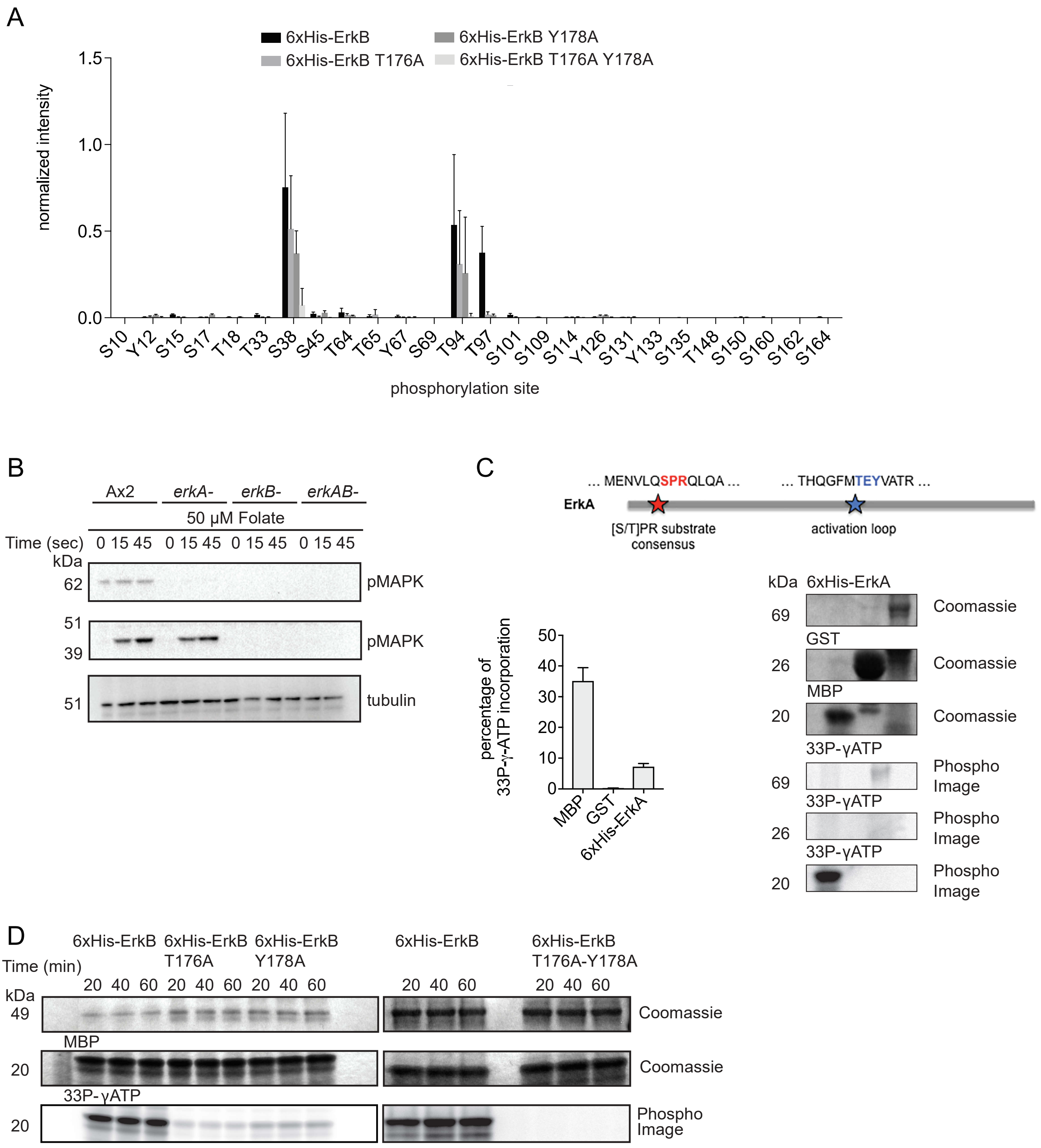
A
6xHis-ErkB
6xHis-ErkB T176A
6xHis-ErkB Y178A
6xHis-ErkB T176A Y178A
normalized intensity
1.5
1.0
0.5
0.0
S10 Y12 S15 S17 T18 T33 S38 S45 T64 T65 Y67 S69 T94 T97 S101 S109 S114 Y126 S131 Y133 S135 T148 S150 S160 S162 S164
phosphorylation site
B
Ax2
erkA-
erkB-
erkAB-
50 µM Folate
Time (sec) 0 15 45 0 15 45 0 15 45 0 15 45
kDa
62
51
39
51
pMAPK
pMAPK
tubulin
C
... MENVLQSPRQLQA ...
... THQGFMTEYVATR ...
ErkA
[S/T]PR substrate consensus
activation loop
percentage of 33P-γ-ATP incorporation
50
40
30
20
10
0
MBP
GST
6xHis-ErkA
kDa 6xHis-ErkA
69
GST
26
MBP
20
33P-γATP
69
33P-γATP
26
33P-γATP
20
Coomassie
Coomassie
Coomassie
Phospho Image
Phospho Image
Phospho Image
D
6xHis-ErkB
6xHis-ErkB T176A
6xHis-ErkB Y178A
6xHis-ErkB
6xHis-ErkB T176A-Y178A
Time (min)
20 40 60 20 40 60 20 40 60 20 40 60 20 40 60
kDa
49
MBP
20
33P-γATP
20
Coomassie
Coomassie
Phospho Image

Figure S4. Identification of ErkA as a ErkB substrate and analysis of ErkB activation loop and autophosphorylation. Related to Figure 5.

(A) Mass spectrometry analysis of phosphorylation sites on MBP substrate following the kinase assay with wild-type and activation loop point mutants of 6xHis-tagged ErkB. Phospho-site intensity is normalized such that the most abundant phospho-site signal in each experimental replicate = 1. Bar chart shows mean of N=3 experiments. Error bars indicate SD. No phosphorylation was detected in samples containing only MBP.

(B) Western blot of folate stimulated Ax2 wild-type and *erkA*$^-$, *erkB*$^-$ and *erkAB*$^-$ mutant cells. Activation loop phosphorylation was analysed using a pMAPK antibody. ErkA activation loop phosphorylation could only be detected in wild-type cells and was abolished in *erkA*$^-$, *erkB*$^-$ and *erkAB*$^-$ cells, indicating a dependency of ErkA phosphorylation on ErkB. Tubulin blot shown as loading control.

(C) Schematic overview of ErkA highlighting important structures present in the protein. The red star symbolises the position of the SPR motif close to the N-terminus of the protein. The blue star marks the position of the activation loop. Bacterial expressed 6xHis-ErkA was used for kinase assays. Both the paper-based assay and the gel assay show a direct phosphorylation of 6xHis-ErkA by 6xHis-ErkB. GST served as negative control while MBP was used as positive control. Coomassie gels are shown as loading controls. Graph shows the mean of three experiments. Error bars indicate SD.

(D) 6xHis tagged ErkB and activation loop mutants were incubated with Myelin Basic Protein (MBP) to assay their kinase activity by monitoring 33P-ATP incorporation. Different incubation times were used (20min, 30min and 40min) While the single mutants T176A and Y178A show a reduced activity independent of the incubation time, no substrate phosphorylation was detected in the T176A Y178A double mutant. Coomassie gels are shown to confirm equal sample loading. The phosphoimages illustrate phosphorylation of MBP.

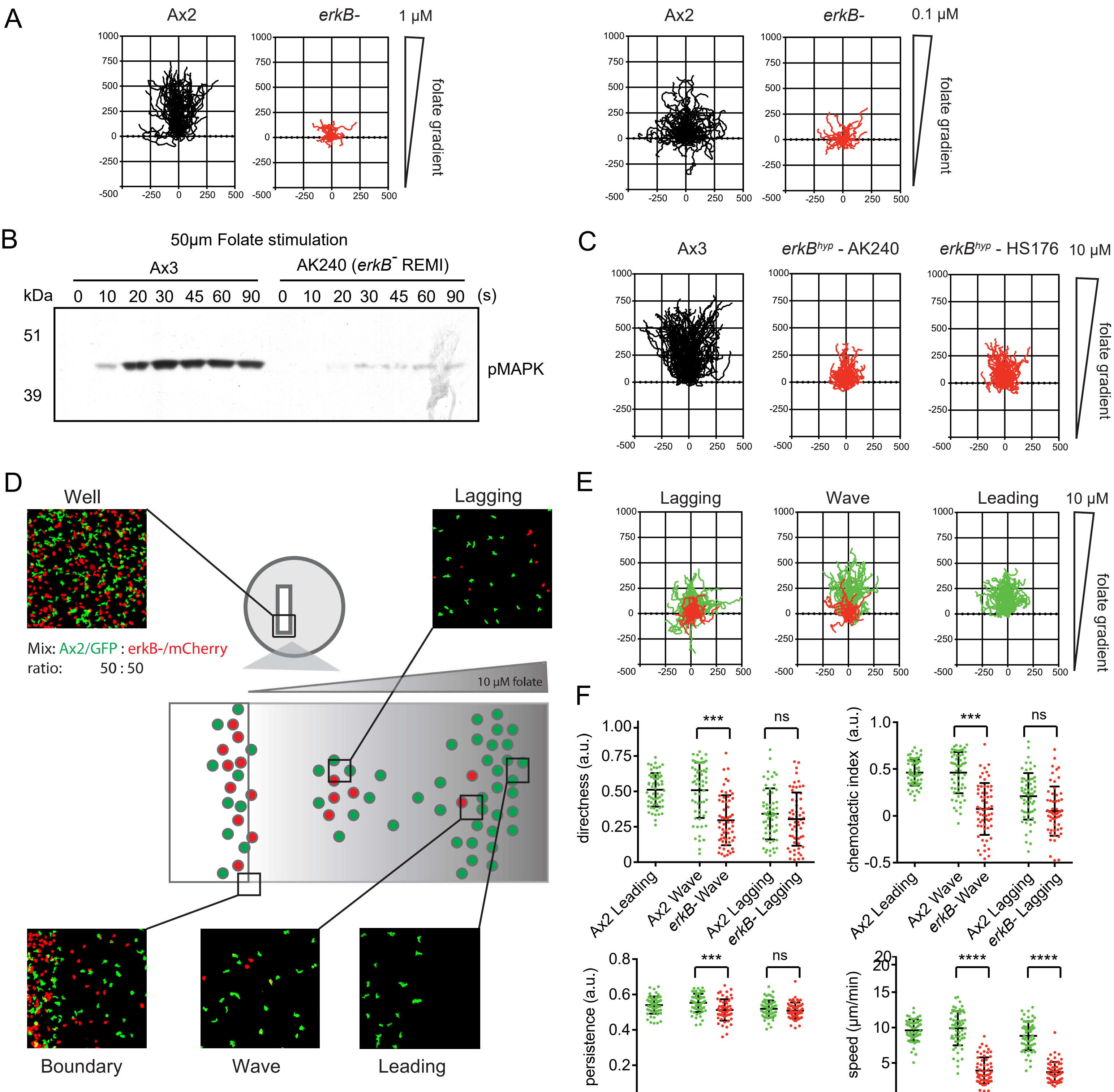

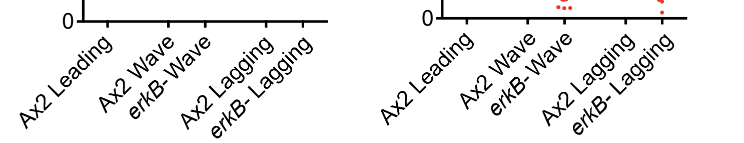

G

10 µM folate	Ax2 (wave)	erkB- (wave)	Ax2 (lagging)	erkB- (lagging)
Chemotactic Index (a.u.)	0.463<br>SD ± 0.219	0.074 ****<br>SD ± 0.275	0.210<br>SD ± 0.246	0.0506 ns<br>SD ± 0.260
Speed (µm/min)	9.902<br>SD ± 2.401	3.946 ****<br>SD ± 1.816	8.852<br>SD ± 3.240	3.879 ****<br>SD ± 1.567
Persistence (a.u.)	0.553<br>SD ± 0.051	0.512 ***<br>SD ± 0.060	0.518<br>SD ± 0.048	0.509 ns<br>SD ± 0.045
Directness (a.u.)	0.507<br>SD ± 0.194	0.300 ***<br>SD ± 0.174	0.341<br>SD ± 0.178	0.305 ns<br>SD ± 0.186

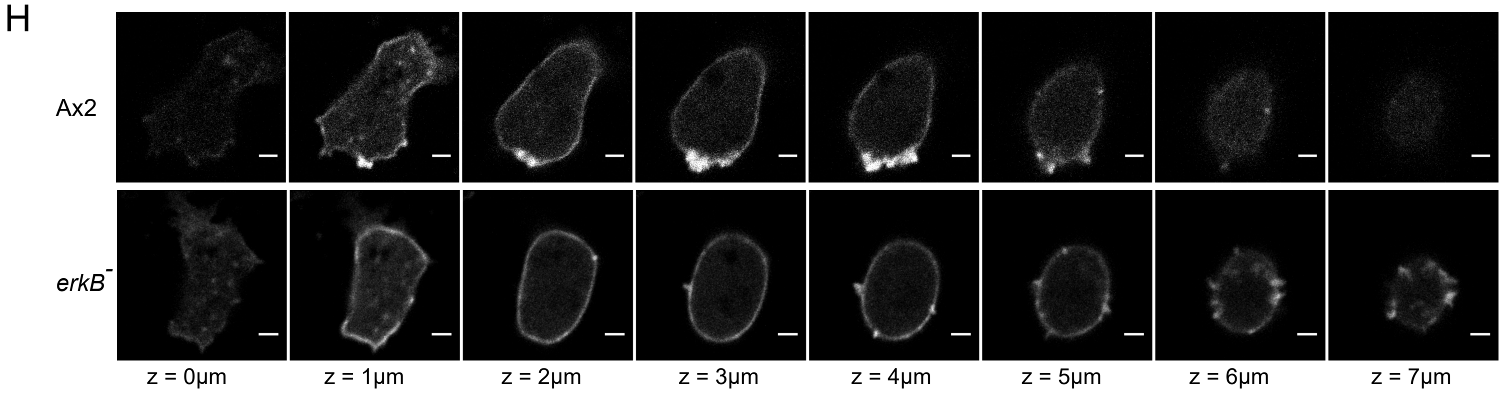

Figure S5. Chemotaxis and cytoskeletal phenotypes of *erkB⁻* mutants. Related to Figure 6.

(A) Individual cell tracks for undifferentiated wild-type (Ax2) and *erkB⁻* amoebae during chemotaxis under agarose containing 1 µM and 0.1 µM folate. N=60 cells. Scale in micrometres. See Figure 6F for chemotaxis parameters.

(B) Immunoblotting timecourse of ErkB phosphorylation following 50 µM folate stimulation of wild-type cells and of AK240 *erkB⁻* REMI mutant cells (hypomorph), showing residual ErkB activation in the AK240 insertional mutant cells.

(C) Individual cell tracks for undifferentiated wild-type (Ax3) and *erkB* hypomorph mutant amoebae during chemotaxis under agarose containing 10 µM folate. N=100 cells, ≥ 20 cells of three independent assays. Scale in micrometres. See Figure 6G for quantification of chemotaxis parameters.

(D) Scheme of under agarose chemotaxis cell mixing experiments. Chemotaxis in the under agarose assay results from amoebae degrading folate present in the agarose. This results in production of a self-generated chemoattractant gradient in which the cells chemotax as a wave. To test whether chemotaxis defects of *erkB⁻* cells in this assay are due to a defect in folate breakdown, wild-type and mutant amoebae expressing GFP or mCherry respectively were mixed in a 1:1 ratio. Both wild-type and *erkB⁻* cells were placed in a well cut in an agarose sheet containing 10 µM folate. Cells were able to move underneath the agarose layer (Boundary). Chemotaxis was filmed and cells tracked in the leading front of cells, in the main body of the cell wave, and in the region lagging behind the cell wave.

(E) Cell tracks of wild-type (green) and *erkB⁻* (red) cells in leading, wave and lagging regions. No *erkB⁻* cells were observed at the leading edge of the cell wave. N=60 cells, 20 cells from each of three independent assays. Scale in micrometres.

(F) Chemotaxis parameters of cells in different regions. In the main cell wave, where wild-type amoebae are able to chemotax, *erkB⁻* amoebae show defective speed, directionality and chemotactic index in the same gradient. Bar indicates mean of all cells. Error bars show SD. Statistical analysis were performed using Welch's t-tests, ****$p < 0.0001$, ***$p < 0.001$.

(G) Direct comparison of speed, chemotactic index, persistence and directness. Statistical analyses were performed using Welch's t-tests, ****$p < 0.0001$, ***$p < 0.001$.

(H) Images of F-actin localisation in wild-type and *erkB⁻* vegetative amoebae assayed by lifeact:mCherry fluorescence. Images are confocal z-planes at 1 µm intervals from the ventral cortex of cells upwards. F-actin puncta are observed within the ventral 2 µm of both wild-type and *erkB⁻* cells. Scale bar = 1µm

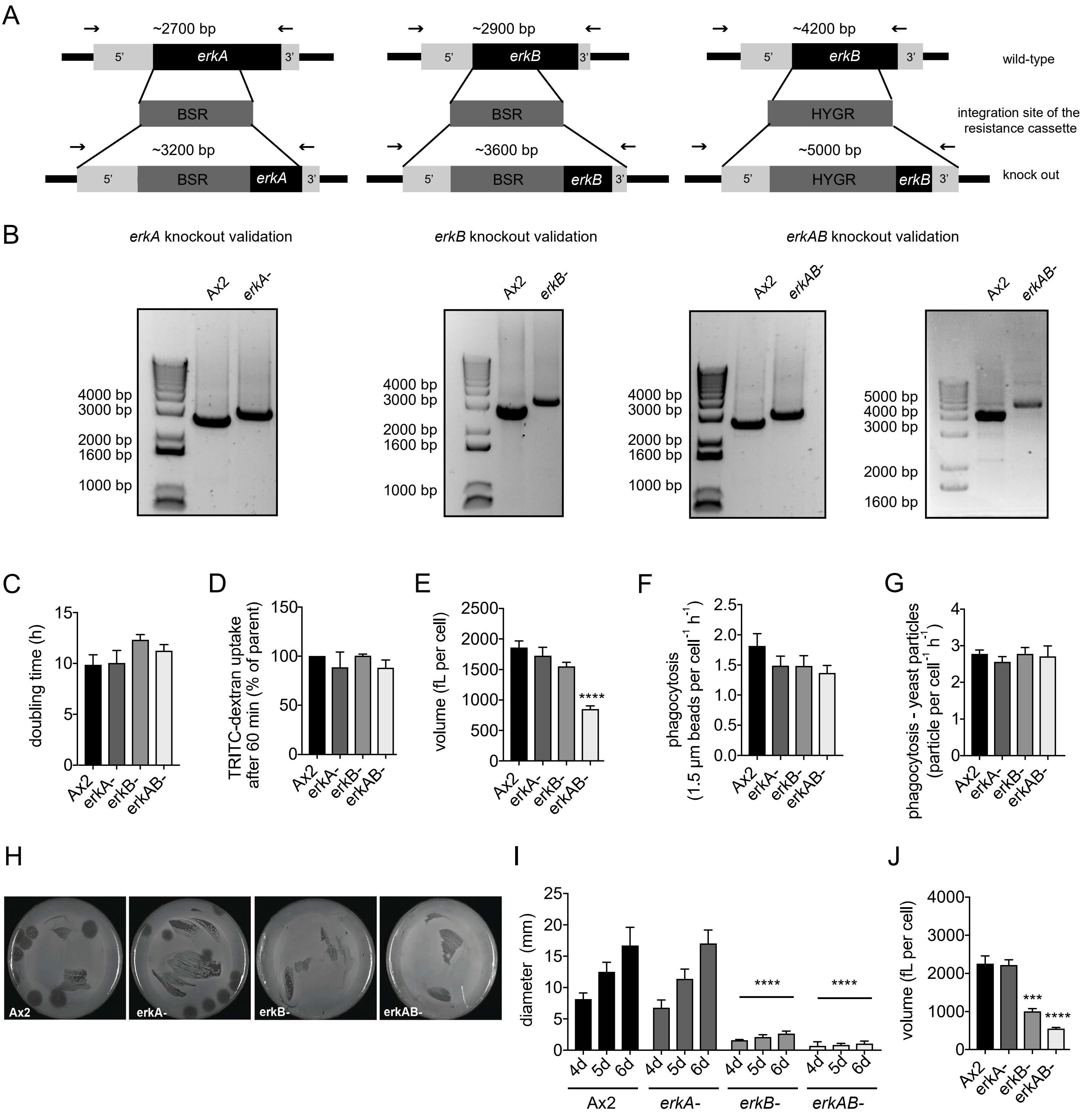
A
~2700 bp
5'
erkA
3'
BSR
~3200 bp
5'
BSR
erkA
3'
~2900 bp
5'
erkB
3'
BSR
~3600 bp
5'
BSR
erkB
3'
~4200 bp
5'
erkB
3'
HYGR
~5000 bp
5'
HYGR
erkB
3'
wild-type
integration site of the resistance cassette
knock out
B
erkA knockout validation
erkB knockout validation
erkAB knockout validation
Ax2
erkA-
erkB-
erkAB-
5000 bp
4000 bp
3000 bp
2000 bp
1600 bp
1000 bp
C
doubling time (h)
D
TRITC-dextran uptake after 60 min (% of parent)
E
volume (fL per cell)
****
F
phagocytosis (1.5 µm beads per cell⁻¹ h⁻¹)
G
phagocytosis - yeast particles (particle per cell⁻¹ h⁻¹)
Ax2
erkA-
erkB-
erkAB-
H
Ax2
erkA-
erkB-
erkAB-
I
diameter (mm)
****
****
4d
5d
6d
Ax2
erkA-
erkB-
erkAB-
J
volume (fL per cell)
***
****

Figure S6. Mutant validation and analysis of the physiological behavior of *erkA*⁻, *erkB*⁻ and *erkAB*⁻ cells. Related to Figure 3 and 6.

(A) Schematic overview of the strategy used to knock out *erkA* and *erkB*. The *erkA* gene was disrupted using a BS resistance cassette while two different constructs were created to interrupt the *erkB* gene locus, one using a BS and another one using a Hygromycin resistance cassette. Light grey bars indicate the cutoffs of the recombination arms used, dark grey bars show the resistance cassettes, while black bars indicate the gene coding sequence. Black lines represent regions down stream and up stream of the targeted gene, while arrows indicate primer binding regions used for the identification of knockout mutants. The site of insertion of the restriction cassette is indicated. The sizes of fragments expected in wild-type and knock out mutants is shown.

(B) PCRs confirming knockout mutants for *erkA*⁻, *erkB*⁻ and *erkAB*⁻ cell lines. The primer combinations indicated in (A) were used to confirm the accuracy of the three erk mutant strains.

(C) Axenic growth of *erkA*⁻, *erkB*⁻ and *erkAB*⁻ cells was measured in shaking suspension. Growth rates were compared. The doubling times of the mutant strains are shown. No significant differences were observed.Graphs show mean of three experiments. Error bars indicate the SEM.

(D) The ability to conduct bulk fluid uptake was assayed in same mutant strains. TRITC-dextran uptake was analysed after 60 min using flow cytometry. Results are plotted relative to Ax2. No fluid uptake defect was observed. Bar graphs show the mean of three independent measurements, error bars show the SEM.

(E) The cell volume of axenically grown mutant strains was measured. Cell volume was determined using an Eclipse flow cytometer. Bar graphs show the mean of three measurements. Significant decrease in cell volume was found for *erkB*⁻ and *erkAB*⁻ cells. While *erkB*⁻ show just a mild decrease in cell volume, *erkAB*⁻ are half the size of the parent strain Ax2. Statistical analyses were performed using a Welch's t-test (*$p<0.1$, ****$p<0.0001$). Error bars show the SEM.

(F) The phagocytosis of small particles was analysed in all mutant strains using 1.5 µm beads. Cells were incubated with fluorescent latex beads for 60 min, washed free of residual beads and uptake was analysed by flow cytometry. *erkA*⁻, *erkB*⁻ and *erkAB*⁻ cells showed a mild but non-significant defect in bead uptake. Plot shows mean bead uptake per cell per hour for five independent measurements. Statistics show a Welch's t-test (***$p<0.001$). Error bars show SEM. Values were compensated for cell size.

(G) Yeast particle uptake was performed using microscopy. Cells were fed for 60 min and quenched with trypan blue before analysis. 100 *Dictyostelium* cells were analysed per cell line and experiment. Bar graphs show the means of three experiments. Similarly to the 1.5 µm bead uptake assy, all knockout cell lines behave like the parent strain. Statistics were performed using a Welch's unpaired t-test (***$p<0.001$, ****$p<0.0001$). Error bars show the SEM. Values were compensated for cell size.

(H) Photographs of plaques of analysed mutant cell lines, illustrating the ability to grow on bacterial lawn. *erkB*⁻ and *erkAB*⁻ cells show a strong growth defect. Cells were grown in conjunction with *K. aerogenes* bacteria for 6 days before imaging.

(I) Quantification of bacterial growth assays on bacterial lawns. Parent and mutant strains were grown in conjunction with *K. aerogenes* bacteria. *Dictyostelium* cells were plated at high dilution to ensure single colonies were formed. The diameter of plaques was measured after 4d, 5d and 6d. At least 5 plaques were analysed per experiment. As shown in (H) *erkB*⁻ and *erkB*⁻ cells have severe growth defects on bacterial lawns, showing a 10-fold decreased plaque size. Bar graphs show the mean of the diameters from three experiments. Statistics were performed using a Welch's t-test (****$p<0.0001$). Error bars show the SEM.

(J) The cell volume of bacterial grown mutant cell strains was investigated. The volume was measured using an Eclipse flow cytometer. The mean of three independent measurements is shown as bar graphs. The *erkA*⁻ cells have a similar cell volume as the parent strain while *erkB*⁻ and *erkAB*⁻ cells show a 2 fold to 4 fold reduced cell volume. The volume is plotted as bar graphs in fL per cell. The mean of five experiments is shown. Statistics were performed using a Welch's t-test (***$p<0.001$, ****$p<0.0001$). Error bars show the SEM.

A

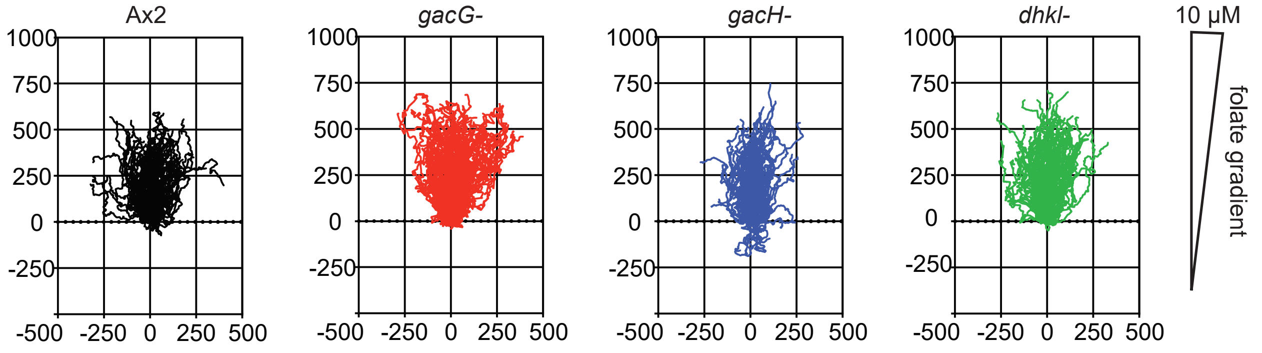

B

10 µM folate	Ax2	gacG-	gacH-	dhkl-
Chemotactic Index (a.u.)	0.487<br>SD ± 0.124	0.453 ns<br>SD ± 0.087	0.467 ns<br>SD ± 0.206	0.539 ns<br>SD ± 0.097
Speed (µm/min)	12.280<br>SD ± 2.008	20.140 ****<br>SD ± 5.102	11.460 ns<br>SD ± 2.073	15.060 ns<br>SD ± 2.452
Persistence (a.u.)	0.553<br>SD ± 0.033	0.545 ns<br>SD ± 0.047	0.564 ns<br>SD ± 0.054	0.570 ns<br>SD ± 0.038
Directness (a.u.)	0.515<br>SD ± 0.114	0.471 ns<br>SD ± 0.085	0.505 ns<br>SD ± 0.168	0.553 ns<br>SD ± 0.090

C

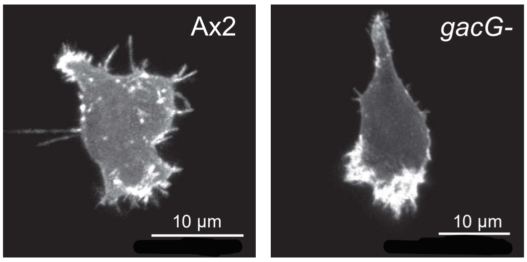

D

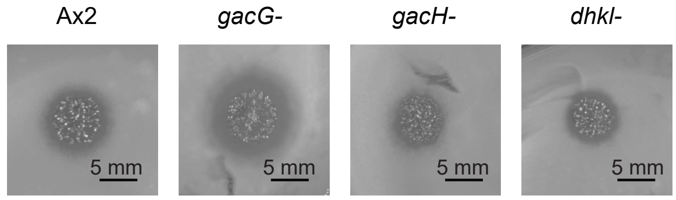

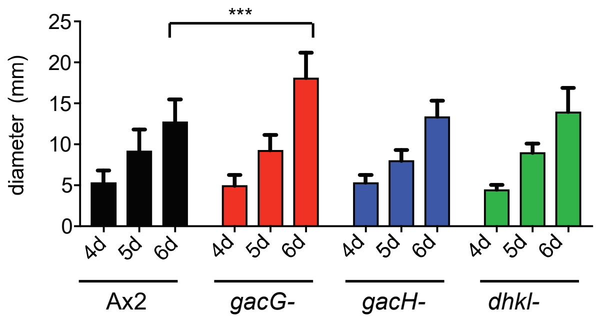

E

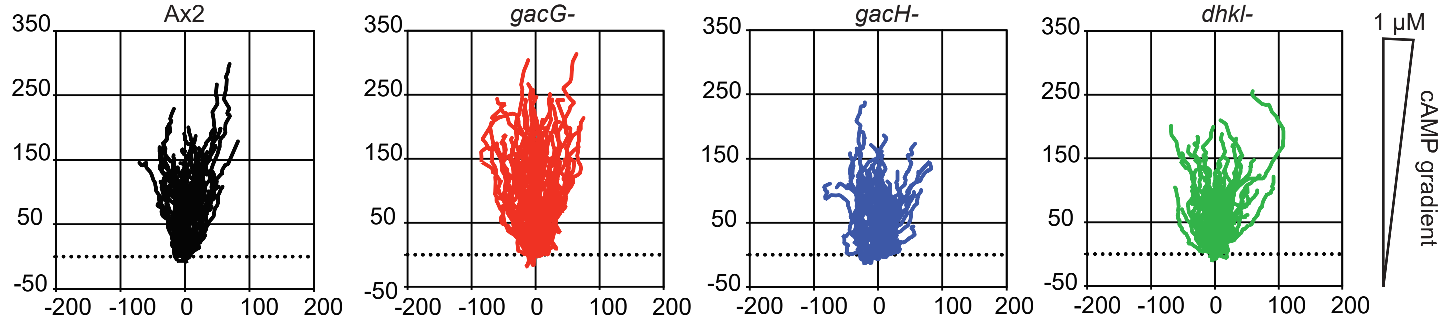

F

1 µM cAMP	Ax2	gacG-	gacH-	dhkl-
Chemotactic Index (a.u.)	0.805<br>SD ± 0.046	0.794 ns<br>SD ± 0.091	0.675 ***<br>SD ± 0.1069	0.728 *<br>SD ± 0.105
Speed (µm/min)	13.36<br>SD ± 3.752	17.76 ***<br>SD ± 3.864	12.44 ns<br>SD ± 3.601	12.34 ns<br>SD ± 4.224
Persistence (a.u)	0.854<br>SD ± 0.093	0.868 ns<br>SD ± 0.084	0.816 ns<br>SD ± 0.119	0.830 ns<br>SD ± 0.117
Directness (a.u.)	0.8216<br>SD ± 0.044	0.805 ns<br>SD ± 0.090	0.6945 **<br>SD ± 0.108	0.7588 *<br>SD ± 0.089

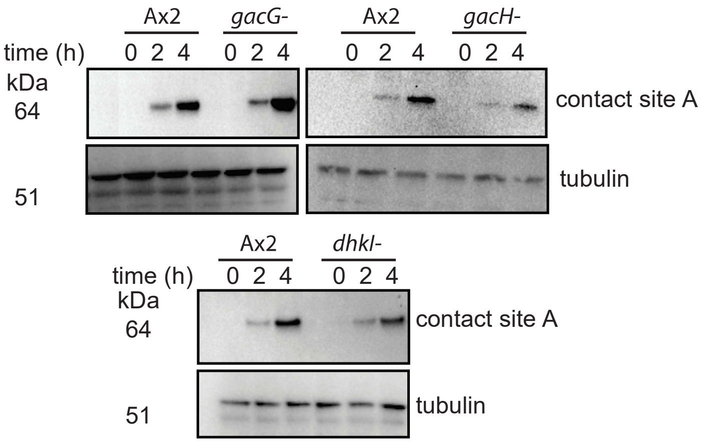

G

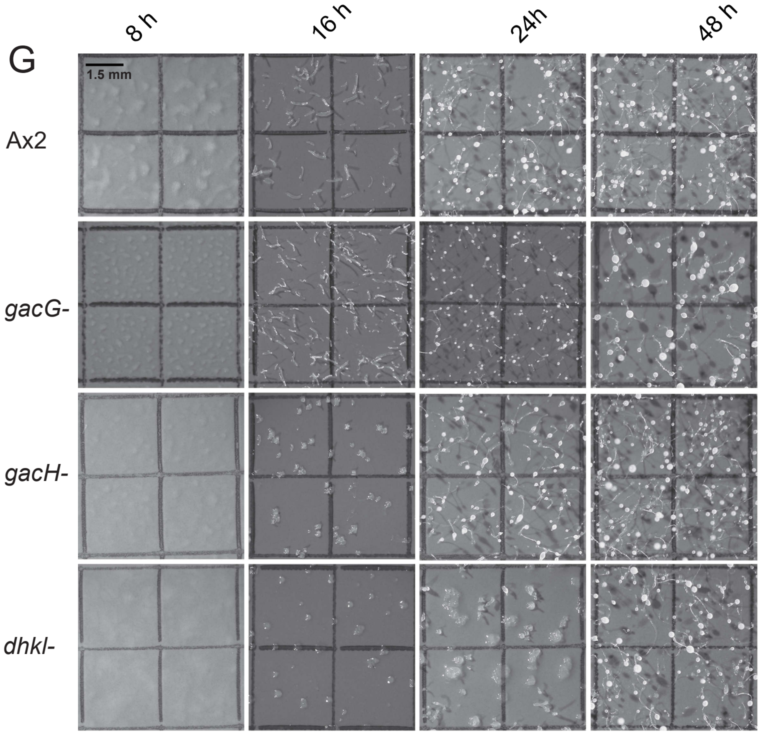

Figure S7. Core proteins show chemotaxis or motility phenotypes. Related to Table 1.

(A) Ax2, *gacG*$^-$, *gacH*$^-$ and *dhkI*$^-$ cells were allowed to chemotax to folate under agarose and examined by microscopy. The tracks of 100 cells per cell line were plotted (The triangle indicates the direction of the folate gradient; scale in micrometres. ≥ 20 cells of three independent assays were analysed).

(B) Quantification of chemotactic index, speed, persistence and directness of wild-type Ax2 and knockout cells plotted in (A). Only *gacG*$^-$ cells show an increase in cell speed. The chemotactic index and directionality are wild-type as in all assayed cell lines. Presented is the mean ± SD. Statistical analyses show Welch's t-tests, ****p < 0.0001, ns >0.1.

(C) Comparison of undifferentiated wild-type Ax2 and *gacG*$^-$ amoebae expressing the F-actin marker lifeact::mCherry. Cells were grown in bacterial suspension and washed free of bacteria before imaging. Shown are Z-projections of confocal sections. *gacG*$^-$ cells are more strongly polarized than Ax2 cells. Scale bar = 10 µm.

(D) Representative images and quantification of the plaque diameters of Ax2, *gacG*$^-$, *gacH*$^-$ and *dhkI*$^-$ cells. *Dictyostelium* amoebae were grown in conjunction with *K. aerogenes* bacteria on SM agar plates using high dilutitions to ensure the formation of single plaques. Diameters were quantified after 4d, 5d and 6d of growth. Per time point and cell line at least 5 single plaques were measured (N=3). Images display a single representative plaque for each cell line, photographed on day 6. *gacG*$^-$ cells exhibit an increase in plaque size after day 6. Graphs show the mean ± SD. Statistical analysis was performed using a Welch's t-test, ***p < 0.001.

(E) Ax2, *gacG*$^-$, *gacH*$^-$ and *dhkI*$^-$ cells were allowed to chemotax to cAMP in an Insall chamber examined by microscopy. The tracks of 60 cells per cell line were plotted to illustrate their chemotactic behavior. Triangle indicates direction of the cAMP gradient; scale in micrometres. 20 cells of three independent assays were analysed.

(F) The chemotactic index, speed, persistence and directness of wild-type Ax2 and knockout cells plotted in (E) were quantified. Displayed is the mean ± SD. Statistical analyses show Welch's unpaired t-tests, ****p < 0.00001, ns >0.1. Western blot analysis was used to check if developmental genes are expressed at correct time points. Samples of cAMP pulsed cells were taken at the indicated time points. The contact site A antibody confirms a proper developemental timing in all knockout mutants. Tubulin was used as loading control.

(G) Developmental time course of all four strains. Cells were developed on gridded filters. Images were captured with a low magnification microscope every 8 h with a final image after 48 h confirming all cell lines produce mature fruiting bodies. Scale bar = 1.5 mm.

Protein	Role	10s cAMP	45s cAMP	360s cAMP
cAR1	cAMP receptor	*S299**<br>**S302***<br>**S303***<br>*S324**<br>*S325**	*S324**	*S299**<br>*S325**
Gα2	Gα subunit			*S113**
RckA	RGS protein		**S83**	**S349**<br>**S351**
GefR	GEF for RasG	**S434**<br>**T860**	**S433**<br>**S468**<br>**S846**<br>**S858**<br>**T860**	**S310**
Sca1	Ras scaffold protein	**S359***<br>**S923**	**T357**<br>**S359***<br>**S372**<br>**S401**<br>**S920**<br>**S921**<br>**S923**<br>**S946**	**S355**<br>**S359***<br>**S920**<br>**S946**
RipA	TORC2 component	**S301**	**T300**<br>**S301**	**S301**<br>*S642*
PikI	PIP5K		**T262***<br>*T338*<br>*S585*	
SHAPS	PKB substrate	**T395**	**S258**	**S258**
PikA	PI3K	**S199**	**S199**	**S199**
PikB	PI3K	**S518**	**S518**<br>**S536**	**S518**<br>**S536**<br>**S542**
GbpC	cGMP binding protein	**S1566**<br>**S2322**	**S1566**<br>**S2262**<br>**T2312**<br>**S2322**<br>**T2329**	**S2322**
DocA	DOCK RacGEF		**S12**<br>**S480**<br>**S481**<br>**S486**<br>**S756**<br>**S762**	**T15**
ElmoE	Gβγ effector	*S994*	*S994*	*S925*<br>**S1675**
MyoE	Myosin I	**S334**	**S334**	
MyoG	MyTH/FERM myosin	**S1593**	**S1504**<br>**S1593**	**T1387**<br>**S1504**
RegA	PDE/response regulator		**S413**	**S413**
Tsunami	Protein kinase		**S579**	**S575**<br>**S579**

Table S1. cAMP responsive phosphorylation sites detected on proteins with known roles in chemotactic signal transduction. Related to Figure 1.

Selected known chemotaxis proteins observed to be phosphorylated in cAMP SILAC experiments are shown. Individual phosphorylation sites are listed. Sites with increased phosphorylation at a given timepoint are shown in bold. Sites with decreased phosphorylation at a given timepoint are shown in italics. Phosphorylation sites previously described in the literature are marked with *.

Site	Protein	Wild type ratio ($\log_2$)	*erkB*$^-$ KO ratio ($\log_2$)	Sequence window	Protein detected in *erkB*$^-$ KO
**Sites upregulated in wild type and not detected in *erkB*$^-$ KO**					
T1178	DhkI-1	5.7	ND	YSNLISTPRGGVG	●
S297	GacQ	4.6	ND	SSKNPISPRSPIG	●
T550	DDB_G0270072	3.9	ND	KHNNPITPRQRLN	●
T233	ForA	3.7	ND	GSLSPVTPRVDDG	●
S1093	DDB_G0288915	3.7	ND	TTTNPVSPRFVST	●
S293	GefM	3.6	ND	VKPPPPSPRFSTN	●
T254	MhkD	3.6	ND	LYIVPTTPRPSKS	●
S530	SepA	3.4	ND	NKKLPLSPRQPSS	●
S596	DDB_G0272006	3.2	ND	SGSPSNSPRLFTK	●
T405	DDB_G0268078	3.1	ND	NQKLDSTPRLSPR	●
T963	DDB_G0292746	3.0	ND	DSSIPLTPR____	●
T550	GacHH	3.0	ND	ASPSPLTPRSIAR	●
S536	PikB	3.0	ND	ESDISSSPRSIGS	●
S469	GacG	2.9	ND	LNIIGISPRGTNR	●
S1070	DDB_G0288915	2.8	ND	KSVNPSSPRYTST	●
S335	DDB_G0287765	2.6	ND	NTPLRTSGNNYKN	●
T291	DDB_G0269608	2.5	ND	PNSGISTPRAINR	●
S577	DDB_G0282105	2.4	ND	QSKMTISPRDKDR	●
S1504	MyoG	2.4	ND	ITGAPKSPRNSSE	●
S447	GacG	2.3	ND	SSSSTSSPRVNAP	●
T58	GacY	2.0	ND	APQRKVTFGSRVR	●
T60	DDB_G0273377	1.9	ND	TTPTSTTPRSKSS	●
S994	DDB_G0278311	1.8	ND	DEKKSSSRFFGKS	●
T984	DDB_G0268348	1.8	ND	SSTNTNTPRTIVL	●
S430	GacG	1.7	ND	SSSTSSSPRNNGN	●
S2141	DDB_G0292230	1.6	ND	GLENSGSIAVGRG	●
S752	DDB_G0283821	1.5	ND	SRKISNSDQFTPN	●
S1482	DhkI-1	1.4	ND	TSPPISSPRSNNN	●
S2846	DDB_G0285063	1.4	ND	SFNRIPSPRRILK	●
T573	GacF	1.4	ND	RPRKGSTVQYLNR	●
S30	Kif9	1.4	ND	LRSRSNSSPSTSS	●

S31	DDB_G0292322	1.3	ND	NKSRNNSRESGLS	●
S149	DDB_G0291205	1.3	ND	IQGPTISPRTLSI	●
T431	RapGAP3	1.3	ND	ILPRKSTIIGPNG	●
T262	DDB_G0267588	1.1	ND	NRVRLNTSQRLKM	●
S228	ForA	1.0	ND	SNASLGSLSPVTP	●
**Sites upregulated in wild type with no response in *erkB*$^-$ KO**					
T85	DstA	4.8	0.0	GRSNNLTPRTNQL	●
S390	DDB_G0281657	2.9	0.1	LSFLPNSPRNPIS	●
S833	DDB_G0278995	2.6	0.2	VRFSYLSPRGGNQ	●
S289	DDB_G0272638	2.1	0.2	LINFQRSPRGTLT	●
S251	KeaA	1.9	-0.1	NGSGQFSPRIHPS	●
S325	EppA	1.5	0.1	RNPFGGSPRNYDN	●
S367	GefS	1.5	-0.2	NIKTGSSSDSFLK	●
S1452	Roco9	1.4	-0.2	KLPSPISPRQSFI	●
S960	AbcC8	1.2	0.1	NKKLNNSGSGVSL	●
**Sites upregulated in both wild type and *erkB*$^-$ KO**					
S618	PikG	5.1	3.8	TNSKSSSSNNLFK	●
T627	GefL	5.0	3.1	LQQTVSTPRQQSF	●
S363	DDB_G0281653	2.2	3.2	NRYRSNSGRDNLP	●
T860	GefR	1.9	2.5	KKERSFTIGLVSG	●
S301	RipA	1.8	2.0	VTFTGTSVKLNSK	●
S572	GacF	1.7	2.1	TRPRKGSTVQYLN	●
S361	DDB_G0281653	1.6	3.0	ALNRYRSNSGRDN	●
S2322	GbpC	1.5	2.4	DGARSGSISYLGR	●
T50	DDB_G0275317	1.2	2.1	QRKRGATVIGTRE	●
S110	DDB_G0279731	1.1	1.7	GRSASLSINNRDA	●
T135	DDB_G0275345	1.1	2.0	DSFRRATINTGSI	●
S847	DDB_G0286003	1.1	1.6	QRSRSNSNSSTAS	●

Table S2. Comparison of phosphorylation changes in wild type and *erkB*$^-$ knockout cells following 45s folate treatment. Related to Figure 4

Phosphorylation sites with increased phosphorylation after 45s folate treatment in wild type cells are listed, grouped by phosphorylation response in wild type and *erkB*$^-$ experiments.

Sequence windows show amino acid sequence context for each phosphorylation site and are colour coded by kinase consensus motif; red for p[S/T]PR sites, yellow for Akt/PKB sites (RxRxxp[S/T]).

ND = site not detected in *erkB*$^-$ experiment. Green dots indicate that other peptides mapping to the same protein were observed in the *erkB*$^-$ experiment, confirming expression of the protein in *erkB*$^-$ cells. Red dots indicate that no other peptides mapping to the same protein were observed in the *erkB*$^-$ experiment. In most cases where a phosphorylation site was not detected in *erkB*$^-$ cells, expression of the protein was detected in the same sample.
