# Supplementary material for: The Atypical MAP Kinase ErkB Transmits Distinct Chemotactic Signals through a Core Signaling Module
Source: Dev Cell. 2019 Feb 25;48(4):491–505.e9. doi: 10.1016/j.devcel.2018.12.001 (PMC6397043; doi:10.1016/j.devcel.2018.12.001)
Supplement: Document S2. Article Plus Supplemental Information [file mmc7.pdf]

Developmental Cell

Article

The Atypical MAP Kinase ErkB Transmits Distinct Chemotactic Signals through a Core Signaling Module

Graphical Abstract

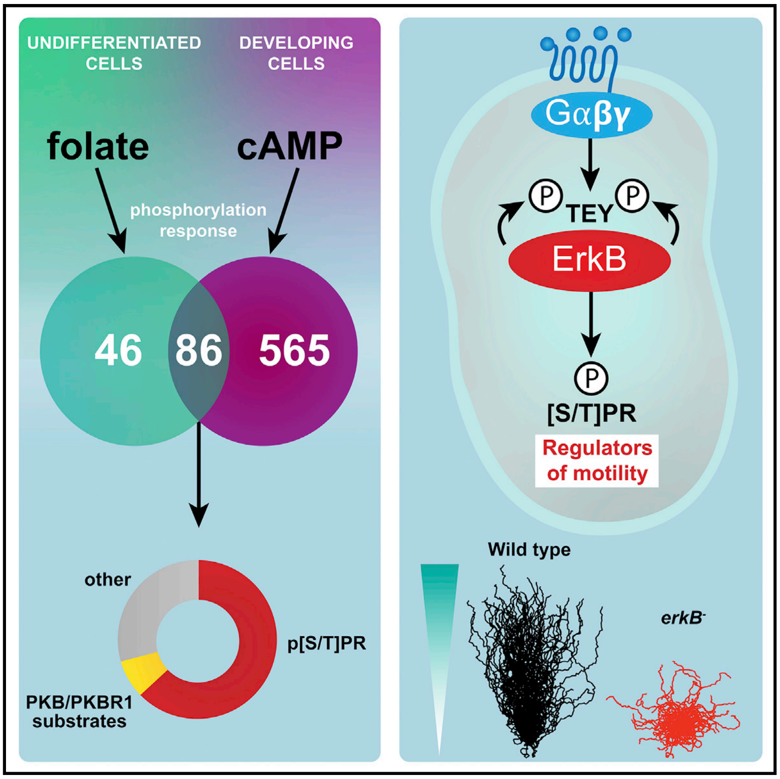

Authors

John M.E. Nichols, Peggy Paschke, Sew Peak-Chew, ..., Elaine Stephens, Jonathan R. Chubb, Robert R. Kay

Correspondence

j.nichols@ucl.ac.uk (J.M.E.N.),
ppaschke@mrc-lmb.cam.ac.uk (P.P.),
rrk@mrc-lmb.cam.ac.uk (R.R.K.)

In Brief

Signaling via chemoattractant receptors activates the cytoskeleton to drive chemotaxis. Nichols, Paschke et al. show that in *Dictyostelium* cells, the atypical MAP kinase ErkB mediates the response to multiple chemoattractants by directly phosphorylating a core set of signaling proteins. Mutants in ErkB and its substrates impair chemotaxis and motility.

Highlights

- Multiple chemoattractants trigger phosphorylation of a core set of proteins
- Most core proteins are phosphorylated by ErkB at a p[S/T] PR motif
- ErkB targets are almost all signaling proteins, not cytoskeletal effectors
- ErkB substrates identify regulators of chemotaxis and motility

Nichols et al., 2019, Developmental Cell *48*, 491–505
February 25, 2019 © 2018 MRC Laboratory of Molecular Biology.
Published by Elsevier Inc.
https://doi.org/10.1016/j.devcel.2018.12.001

Developmental Cell
Article

The Atypical MAP Kinase ErkB Transmits Distinct Chemotactic Signals through a Core Signaling Module

John M.E. Nichols,[1,2,6,7,*] Peggy Paschke,[1,3,6,*] Sew Peak-Chew,[1] Thomas D. Williams,[1] Luke Tweedy,[3] Mark Skehel,[1] Elaine Stephens,[1,4] Jonathan R. Chubb,[5] and Robert R. Kay[1,*]
[1]Cell Biology Division, MRC Laboratory of Molecular Biology, Francis Crick Avenue, Cambridge CB2 0QH, UK
[2]MRC Laboratory for Molecular Cell Biology, University College London, Gower St., London WC1E 6BT, UK
[3]Cancer Research UK (CRUK) Beatson Institute, University of Glasgow, Bearsden, Glasgow G61 1BD, UK
[4]Pfizer Inc, 1 Burtt Road, Andover, MA 01810, USA
[5]MRC Laboratory for Molecular Cell Biology and Department of Cell and Developmental Biology, University College London, Gower St., London WC1E 6BT, UK
[6]These authors contributed equally
[7]Lead Contact
*Correspondence: j.nichols@ucl.ac.uk (J.M.E.N.), ppaschke@mrc-lmb.cam.ac.uk (P.P.), rrk@mrc-lmb.cam.ac.uk (R.R.K.)
https://doi.org/10.1016/j.devcel.2018.12.001

SUMMARY

Signaling from chemoattractant receptors activates the cytoskeleton of crawling cells for chemotaxis. We show using phosphoproteomics that different chemoattractants cause phosphorylation of the same core set of around 80 proteins in *Dictyostelium* cells. Strikingly, the majority of these are phosphorylated at an [S/T]PR motif by the atypical MAP kinase ErkB. Unlike most chemotactic responses, ErkB phosphorylations are persistent and do not adapt to sustained stimulation with chemoattractant. ErkB integrates dynamic autophosphorylation with chemotactic signaling through G-protein-coupled receptors. Downstream, our phosphoproteomics data define a broad panel of regulators of chemotaxis. Surprisingly, targets are almost exclusively other signaling proteins, rather than cytoskeletal components, revealing ErkB as a regulator of regulators rather than acting directly on the motility machinery. ErkB null cells migrate slowly and orientate poorly over broad dynamic ranges of chemoattractant. Our data indicate a central role for ErkB and its substrates in directing chemotaxis.

INTRODUCTION

Migration and chemotaxis are essential functions in normal development and physiology for many cell types, and their corruption underlies devastating human diseases (Artemenko et al., 2014; Jin et al., 2008; Muinonen-Martin et al., 2014). Crawling cells move by projecting pseudopods or blebs at their front while gaining traction from the environment to propel their body forward (Zatulovskiy et al., 2014). In chemotaxing cells, this basic motility is steered by gradients of environmental signals. These signals are detected through surface receptors, which signal to the actin cytoskeleton, biasing projections toward the gradient.

Chemotactic signaling in *Dictyostelium* has been studied extensively and informs our understanding of chemotaxis in neutrophils and other cell types (Graziano and Weiner, 2014). Key regulators of *Dictyostelium* chemotactic signaling have been grouped into multiple pathways, of which Ras-PI3K-PKB, Ras-TORC2-PKB, and cGMP-myosinII have attracted the most attention. Yet with the exception of the cascade from Gβ, via RacB, to Arp2/3 (Yan et al., 2012), the path from upstream signaling events to effectors of motility remains unclear. The small GTPases Ras, Rap, and Rac are crucial, but control of their activity in time and space by large families of guanine nucleotide exchange factors (GEFs) and GTPase activating proteins (GAPs) is barely understood (Kortholt et al., 2013). As we do not know how much of the regulatory network has been identified, it is difficult to understand the global organization and flow of information from chemoattractant to motile behavior. For example, is the regulation distributed throughout the network, or focused through a limited number of nodes? To what extent are different chemotactic stimuli differentially processed by the cell? What types of signals are used at different levels of hierarchy in the network? These questions suggest that a global approach could yield important insights into chemotactic signaling.

To decipher organizational principles and dynamics of the signaling networks driving directed migration, we have used quantitative phosphoproteomics (Olsen et al., 2006) to identify proteins that become rapidly phosphorylated or dephosphorylated in response to different chemoattractants in *Dictyostelium* (Pan et al., 2016; Sugden et al., 2015). Our results demonstrate that a core set of regulatory proteins is shared among chemoattractants. Remarkably, more than half are phosphorylated at a consensus [S/T]PR motif by a single protein kinase, ErkB. Null mutants have defects in both speed of movement and gradient sensing, across a broad spectrum of concentrations and shapes of chemoattractant gradients. ErkB targets found in our data identify a diverse set of regulators of chemotaxis and motility. The extent of the target set implies that the chemotactic network

 © 2018 MRC Laboratory of Molecular Biology. Published by Elsevier Inc.
This is an open access article under the CC BY license (http://creativecommons.org/licenses/by/4.0/).

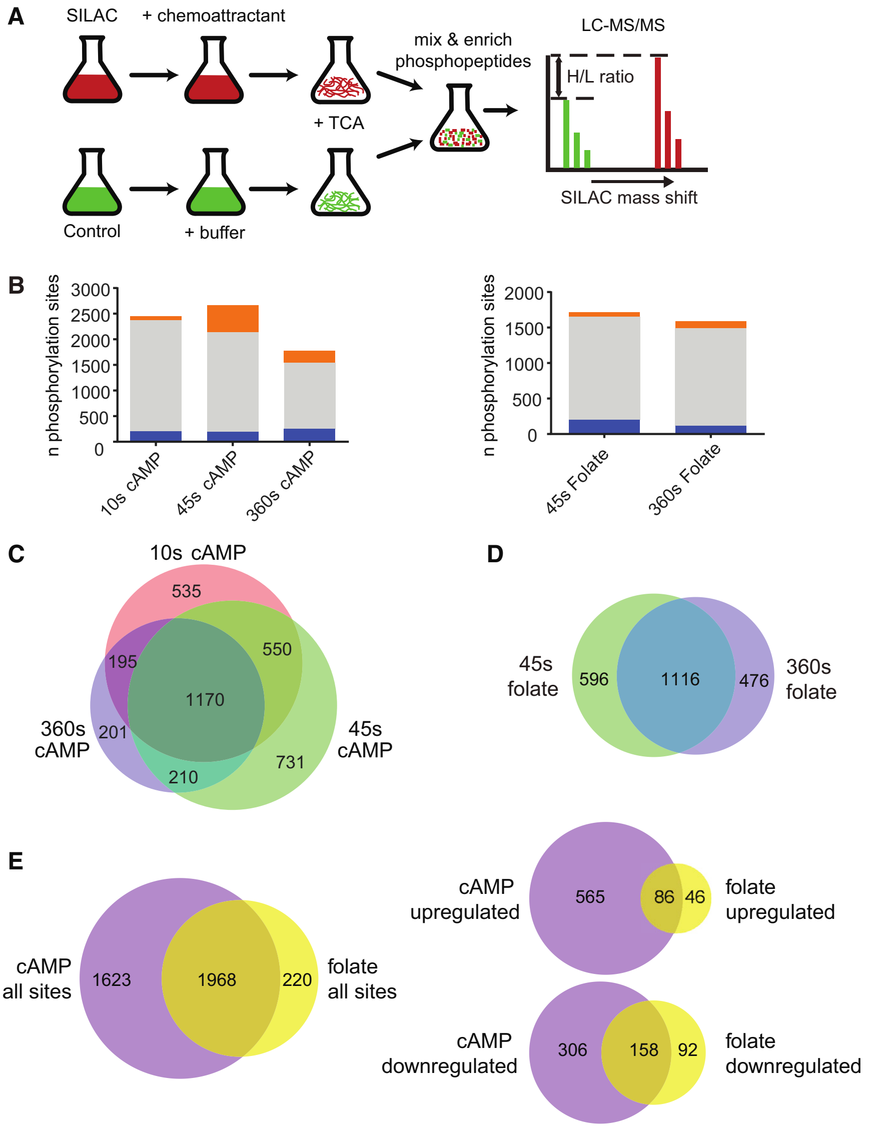

Figure 1. Phosphorylation Changes Following Chemoattractant Treatment

(A) SILAC experimental procedure. SILAC labeled cells were stimulated with chemoattractant. Unlabeled cells were treated with buffer. Lysis and protein denaturation by trichloroacetic acid (TCA) rapidly terminated signaling. Phosphopeptide enrichment was performed using TiO_2 and IMAC columns. SILAC mass shift in LC-MS/MS allowed identification and quantification of protein phosphorylation in stimulated and control cells.

(B) Number of phosphorylation sites identified, broken down into increased (orange), decreased (blue), and unchanged (gray) phosphorylation, using a 2-fold threshold. See Figure S1 and Tables S1 and S3 for details of chemoattractant-induced phosphorylation changes, phosphoproteins, and full phosphoproteomics data.

(C) Overlap in phosphorylation sites identified in cAMP experiments.

(D) Overlap in phosphorylation sites identified in folate experiments.

(E) Intersection of phosphorylation sites (left) and chemoattractant responses (right) observed in cAMP and folate experiments.

See Table S4 for phosphoproteomics data intersection.

has previously been substantially undersampled. Overall, this study reveals a central role for ErkB and its substrates in directing chemotaxis.

RESULTS

Identification of a Core Set of Chemotaxis Phosphoproteins

We used SILAC labeling and mass spectrometry to identify proteins whose phosphorylation changes in response to cAMP, the best-studied chemoattractant in *Dictyostelium*. Cells were grown for 5 generations with or without heavy isotopes and developed to aggregation competence, where they are responsive to cAMP. Heavy cells were uniformly stimulated with a saturating dose of cAMP for 10, 45, and 360 s at 15°C to sample across the timescale of acute responses (Sobczyk et al., 2014; Swaney et al., 2010), stopped with TCA, and mixed with similarly treated unstimulated light cells (Figure 1A). Liquid chromatography tandem mass spectrometry (LC-MS/MS) analysis of extracted phosphopeptides identified 18,756 individual phosphorylation sites.

Only phosphorylation sites of high technical quality were analyzed further (Figures 1B and 1C; Table S3). Of these 3,591 sites, we selected those with at least a 2-fold change in phosphorylation, giving 651 with increased and 464 with decreased phosphorylation (Figures 1B, 1C, and S1A). Including multiple events on the same protein, this gave 689 proteins whose phosphorylation changed in response to cAMP (Figure S1B). Gene ontology (GO) terms related to signal transduction, cytoskeleton, chemotaxis, and cAMP relay were enriched in the stimulated set (Figure S1C; Table S4). This set contained proteins known to be involved in cAMP signal transduction (Table S1), including the TORC2 component RipA (Lee et al., 1999), the PI3-kinases PikA and PikB (Hoeller and Kay, 2007), and the protein kinase Tsunami (Tang et al., 2008), with previously known phosphorylations also confirmed on the cAMP receptor, cAR1 (Caterina et al., 1995), the PIP5K PikI (Fets et al., 2014), and the Ras scaffold Sca1 (Charest et al., 2010).

In contrast, there was little GO enrichment among proteins with decreased phosphorylation (Figure S1C), implying that phosphorylation but not dephosphorylation is the primary functional response to cAMP. These dephosphorylated proteins were not studied further.

To examine responses to a second chemoattractant, undifferentiated cells were stimulated with a saturating dose of folate for either 45 or 360 s at 15°C, and 2,188 phosphorylation sites were identified (Table S3). Phosphorylation increased more

Table 1. Phosphoproteins in the Core Phosphoproteome

Gene	Protein Description	p[S/T]PR Motif	PKB Substrate Motif	Mutant	Mutant Phenotype Defect
GxcT	RhoGEF			●	cAMP chemotaxis
RckA	RGS domain kinase	●		●	cAMP chemotaxis
Rapgap3	RapGAP			●	cAMP chemotaxis
MkpA	MAPK phosphatase			●	Aggregation
ForA	Formin	●		●	Chemotaxis and motility
GefR	RasGEF			●	cAMP signal transduction
GefM	RasGEF	●		●	cAMP signal transduction and chemotaxis
EppA	Erk2-dependent phosphoprotein	●		●	cAMP and folate chemotaxis
DstA	STAT family protein	●		●	cAMP chemotaxis
RipA	TORC2 component			●	cAMP chemotaxis and signal transduction
MyoG	MyTH/FERM myosin	●		●	cAMP and folate chemotaxis
Sca1	Ras scaffold protein	●		●	Motility and cAMP chemotaxis. cAMP and folate signal transduction
Lmbd2B	LMBR1 family protein	●		●	cAMP and folate chemotaxis
GbpC	Cyclic GMP-binding protein	●		●	cAMP chemotaxis
DocA	DOCK family RhoGEF	●		●	cAMP chemotaxis
MhkD	MHC kinase	●		●	cAMP chemotaxis and motility
GefS	RasGEF, PKB substrate			●	cAMP chemotaxis (in *pten*$^{-}$ background)
GacG	RhoGAP, PKB substrate	●			
PikB	PI3K	●		●	cAMP chemotaxis (in multiple PI3K KO)
PikG	PI3K			●	cAMP chemotaxis (in multiple PI3K KO)
PikI	PIP5K		●	●	cAMP chemotaxis and signal transduction
DDB_G0293128	Dopey1 orthologue	●			
DDB_G0292746	Patatin family protein	●			
DDB_G0292230					
Set1	H3K4 methyltransferase	●		●	NCPR. Precocious aggregation
DDB_G0289027		●			
DDB_G0288915		●			
Ugt52	Sterol glucosyltransferase	●			
AccA	Acetyl-CoA carboxylase	●			
Roco9	Roco family kinase	●		●	NCPR. No development defect
DDB_G0288121		●			
DDB_G0287961	WD40 repeat, DENN domain				
Vps13A	Vps13 family protein			●	NCPR. Growth defect on *M. luteus*
DDB_G0286003	LRR-containing protein		●		
DDB_G0285063	E3 ubiquitin ligase	●			
DDB_G0283821	STE group protein kinase			●	NCPR. cAMP wave formation
DDB_G0283347	FGF binding protein-like	●			
DDB_G0282895	Morn Tyrosine kinase-like	●			
GacQ	RhoGAP, PKB substrate	●			
GacY	RhoGAP				
DDB_G0281657	E3 ubiquitin ligase	●			
DDB_G0280777	Bromodomain protein	●			
GxcS	RhoGEF				
CtrB	Cationic a.a transporter	●			
DDB_G0279765		●			
DDB_G0279653			●		
DDB_G0278995		●			

(*Continued on next page*)

Table 1. *Continued*

Gene	Protein Description	p[S/T]PR Motif	PKB Substrate Motif	Mutant	Mutant Phenotype Defect
MscS	Mechano-sensitive ion channel	●		●	NCPR. WT Rheotaxis. WT Ca2+ response to ATP/ADP
SepA	Septase	●		●	NCPR. Cytokinesis and delayed aggregation
GefL	RasGEF	●		●	NCPR. cAMP wave, slug taxis
DDB_G0275861		●		●	NCPR. Aggregation
DDB_G0275843	ArfGAP	●			
DDB_G0275345	RabGAP-like				
DDB_G0275317			●		
DDB_G0275315		●			
DDB_G0274847	Nucleotidyltransferase	●			
ArgB	N-acetylglutamate kinase				
DDB_G0274643		●			
UdkB	Uridine kinase				
DDB_G0274425	PP2C-related	●			
GacF	RhoGAP		●		
DhkC	Histidine kinase	●		●	cAMP chemotaxis. Precocious aggregation
DhkI	Histidine kinase	●		●	Suppresses *amiB*$^{-}$ motility defect
DDB_G0273377		●			
GacH	RhoGAP		●		
DDB_G0272638	PIP5K	●			
SgkA	Sphingosine kinase			●	NCPR. Sensitivity to DNA-damage drugs
GacHH	RhoGAP	●			
DDB_G0272006		●			
DDB_G0271844	Vps9 domain protein				
DDB_G0270918	DENN domain protein	●			
DDB_G0270072	Coiled-coil domain	●			
DDB_G0269710		●			
DDB_G0268348		●			
DDB_G0268078	RCK family kinase				
DDB_G0268070		●			
GacO	RhoGAP				
Roco7	Roco family kinase	●		●	NCPR. No development defect

Proteins in the intersection of cAMP and folate phosphorylation responses. Annotations based on experimental evidence or homology. Known chemotaxis-related phenotypes and detail of phosphorylation motifs are listed. NCPR = no chemotaxis phenotype reported in published descriptions of mutant. See dictyBase (Basu et al., 2015) for detail of mutant strains.

than 2-fold at 132 of these sites, decreased more than 2-fold at 250 (Figure 1B), and around half were common to both time points (Figure 1D). GO analysis showed enrichment of cellular locomotion and signal transduction functions among proteins with increased phosphorylation (Figure S1D; Table S4).

To identify core components in chemotactic signaling, we intersected the cAMP and folate protein sets, finding 1,968 common phosphorylation sites, of which 86 showed a 2-fold or greater increase in phosphorylation (Figure 1E; Table S4). This set accounted for more than half of the sites upregulated by folate, but only 15% of those upregulated by cAMP, consistent with the additional roles of cAMP in development (Gerisch et al., 1975; Town et al., 1976) and cAMP relay (Shaffer, 1975), and potentially reflecting additional mechanisms for amplification of cAMP gradient sensing. Accounting for multiple phosphorylations on the same protein gave a core set of 78 proteins whose phosphorylation was stimulated at least 2-fold by both chemoattractants (Table 1). This number is likely to be an underestimate due to intrinsic limitations of the technique, such as the formation of unsuitable peptides by tryptic digestion and the stringent quality criteria.

This set of proteins was strongly enriched for GO terms associated with signal transduction and chemotaxis and includes 9 protein kinases, 9 GEFs, 10 GAPs, and 5 proteins of phosphoinositide metabolism, but only 2 cytoskeletal proteins—a myosin-I and a formin. Mutants have been described in 30 of the 78 core genes (Basu et al., 2015), of which 18 have a described movement or chemotaxis defect and another 6 have a phenotype suggestive of such a defect (for instance, a defect in aggregation) although chemotaxis was not assayed directly (Table 1).

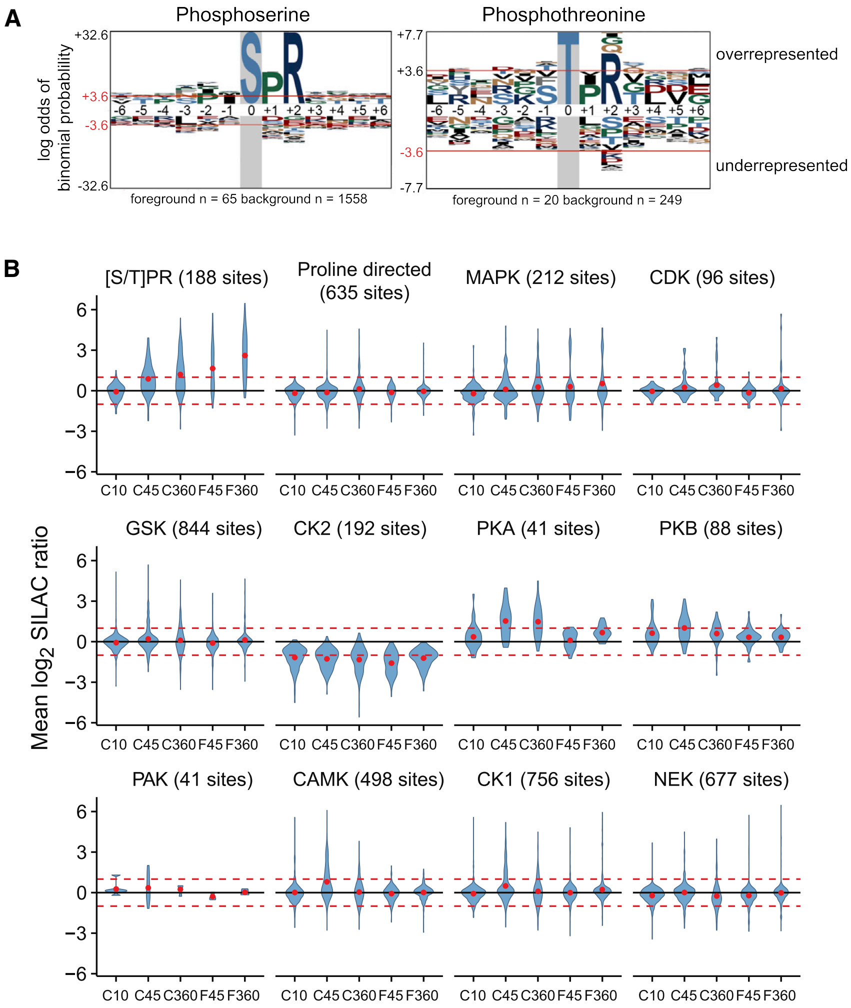

Figure 2. Identification of Chemoattractant-Responsive Phosphorylation Motifs

(A) *De novo* identification of phosphorylation motifs in chemoattractant-responsive phosphorylations. Sequence motifs were built around statistically over-represented residues flanking cAMP and folate responsive phosphorylation sites. The foreground set of 13 mer sequences centered on phosphorylated S or T residues that respond to chemoattractant was compared with a background set of 13 mer sequences for all phosphorylation sites identified in the same experiments. Residue letters scaled to the statistical significance of over- or under-representation. Red line shows p = 0.05. See also Figure S2.

(B) Distributions of SILAC ratios for phosphorylation sites following chemoattractant stimulation, assigned to different known kinase substrate motifs, and the p[S/T]PR motif identified in (A). N of sites matching each motif is shown. See Figure S2 for motif sequences and Table S4 for phosphorylation sites matching the p[S/T]PR motif. The proline-directed category does not include those sites that also match the p[S/T]PR and pMAPK motifs.

This represents significant enrichment of movement and chemotaxis phenotypes among mutants of the core phosphoproteome compared to all the phosphoproteins we identified (p = 0.0002, Fisher's exact test). Despite this enrichment, 48 of the core proteins have no described mutant phenotype to date, implying that the signaling space of chemotaxis has previously been greatly undersampled.

Phosphorylation of the Core Phosphoproteome at a Single Consensus

We searched for motifs in sequences flanking phosphorylation sites of the core proteins, comparing them to all other sites in our data. We found strong overrepresentation of arginine at the +2 position (Figure 2A) associated with proline at +1 (Figure S2A), giving a p[S/T]PR motif. This motif accounted for 54 of the 86 core phosphoproteome sites (48 of 78 proteins; Tables 1 and S4). The combination of proline at +1 and arginine at +2 is not a well-characterized kinase substrate motif, suggesting a limited range of proline-directed kinases might be responsible for phosphorylating most of the core phosphoproteome.

Analysis for over-representation of known kinase motifs in the core phosphoproteome (Figures S2B and S2C) showed that PKB, PKA, and a broad CAMK consensus (Rxxp[S/T]) are over-represented among cAMP stimulated sites. However, only p[S/T]PR showed significant phosphorylation with both chemoattractants. We found enrichment of CK2 motifs among dephosphorylated sites, suggesting that activity of this kinase (or a counteracting phosphatase) is also modulated by chemoattractants.

p[S/T]PR sites were phosphorylated with a characteristic delay after stimulating cells with cAMP, with little increase before 10 s but sustained phosphorylation at 360 s, which was also apparent after folate stimulation (Figure 2B). This sets it apart from for instance PKB sites, where phosphorylation is increased within 10 s but not sustained at 360 s.

Phosphorylation of p[S/T]PR Sites Is Mediated by the MAP Kinase ErkB

Having identified p[S/T]PR as the major phospho-motif in the chemotactic proteome, we sought to identify the kinase responsible for its phosphorylation. We took a genetic approach to test candidate kinases and delineate control of phosphorylation at this site.

We identified an antibody against pTxR (a subset of p[S/T]PR sites), which recognized proteins whose phosphorylation is stimulated by cAMP and folate with similar delayed kinetics to the [S/T]PR sites assayed by mass spectrometry, and of molecular weights and temporal profiles distinct from substrates of the kinases PKB and PKBR1 (Figures 3A, 3B, and S3A).

Using this antibody, we screened mutants in chemotactic signal transduction for defects in phosphorylation response to folate. The response was lost in mutants of the heterotrimeric G-protein subunit Gα4, which is specific to folate (Hadwiger et al., 1994) and Gβ, showing that it is mediated through a

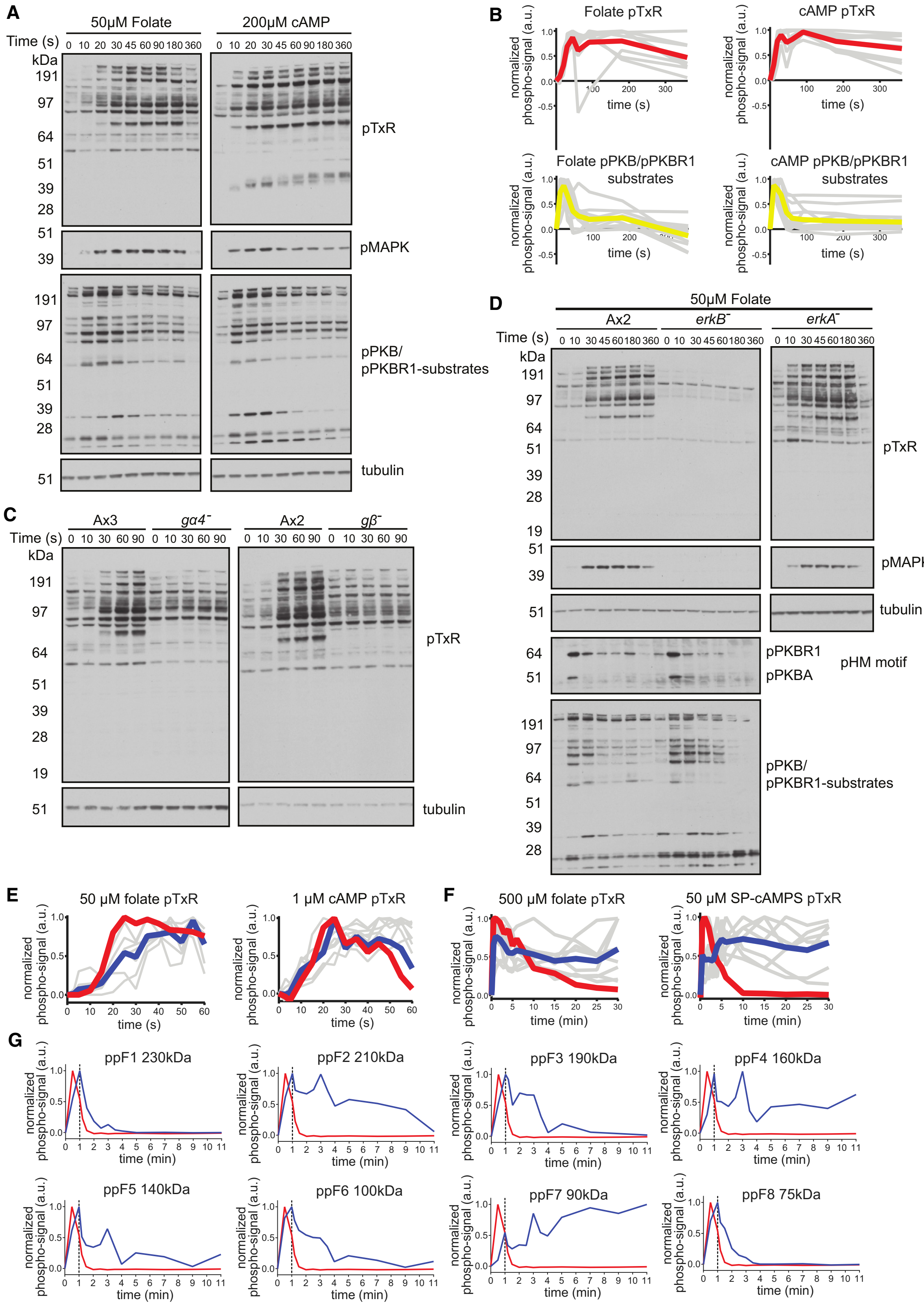

(legend on next page)

GPCR and its coupled heterotrimeric subunit (Figure 3C). Resting levels of phosphorylation were higher in these mutants, suggesting that heterotrimeric G-proteins may mediate both activation and inhibition of phosphorylation in parallel.

To identify additional upstream regulators, we screened a panel of signaling, chemotaxis, and cytoskeleton mutants for aberrant phosphorylation dynamics at [S/T]PR. There was no clear effect on phosphorylation in PKB⁻/PKBR1⁻ double (*pkbA⁻/pkgB⁻*), PI3K1-5⁻ quintuple (*pikA⁻/pikB⁻/pikC⁻/pikF⁻/pikG⁻*) or Rictor⁻ (TORC2) knockout mutants (*piaA⁻*) (Figure S3J), suggesting signaling through these proteins is not required for pTxR phosphorylation. The phosphorylation response was also intact in knockouts of RasC⁻/G⁻, the PI4P5-kinase PikI (that makes most cellular $PI4,5P_2$), GcA⁻/SgaA⁻ (cGMP production), IplA (IP3-receptor required for the calcium response to chemoattractants), the catalytic subunit of PKA, the phospholipase PLA_2, and SCAR/WAVE (data not shown). In short, deletion of components of the pathways most commonly considered to control chemotaxis did not affect phosphorylation of the pTxR sites.

An antibody against the phosphorylated activation loop of MAPK detected a band with kinetics strongly resembling pTxR phosphorylations and of the same molecular weight as the *Dictyostelium* MAPK, ErkB, (Figures 3A and S3; Segall et al., 1995). Following folate stimulation, pTxR phosphorylation lagged behind ErkB phosphorylation (Figures 3E and S3E) but appeared simultaneously with it following cAMP stimulation (Figures 3E and S3F).

ErkB is an atypical MAPK related to mammalian ERK7 and ERK8 (Figure S3B). To test whether ErkB is responsible for pTxR phosphorylations, we created null mutants in ErkB, the classical MAP kinase ErkA (Figure S3B), and double null mutants of both ErkA and ErkB (Figures S6A and S6B). In ErkA knockout mutants, phosphorylation was induced normally by either folate or cAMP, showing ErkA is not responsible for pTxR phosphorylations (Figures 3D and S3C).

We then tested the phosphorylation response of ErkB null mutants to chemotactic stimulation. After 7 hr of development induced by pulses of cAMP, *erkB⁻* cells did not express the developmental marker contact sites A (CsA), indicating they did not develop to a cAMP-sensitive, aggregation competent state, thus confounding experiments with cAMP (Figure S3D). We therefore stimulated undeveloped cells with folate. The results are clear: basal pTxR phosphorylation was almost eliminated in ErkB null mutants and folate no longer stimulated phosphorylation at this site (Figure 3D). In contrast, phosphorylation of the HM motifs of PKB and PKBR1, as well as PKB/PKBR1 substrates, were at wild-type levels (Figure 3D). This shows that phosphorylation of the pTxR motif depends on ErkB, while other folate-stimulated phosphorylation responses do not.

Phosphorylations mediated by ErkB were long-lived compared to known signaling, cytoskeletal, and cell-level responses, most of which adapt (downregulate) within seconds to minutes of stimulation. To better define the timescale of this extended activity, we used conditions to maintain prolonged stimulation of cells, either with high folate or non-hydrolyzable cAMP (Sp-cAMPS). An adenylyl-cyclase mutant was used to avoid complications from endogenous cAMP signaling. Strikingly, with both folate and Sp-cAMPS stimulation, many ErkB target proteins remained persistently phosphorylated for the full 30 min of the time course (Figures 3F, S3G, and S3H). In contrast, phosphorylation of PKB/PKBR1 substrates rapidly adapted over a much shorter timescale (Figure 3B). For both folate and Sp-cAMPS, the activating phosphorylation of ErkB was shorter lived than phosphorylation of many of its targets, with little signal remaining 10 min after stimulation (Figures 3F, S3G, and S3H). After this time, phosphorylation of some targets also decayed, while others remained stable.

To investigate dephosphorylation kinetics after stimulus withdrawal, a dilution protocol was used: cells were stimulated with 200 nM folate and diluted 50-fold after 1 min. ErkB target phosphorylations then disappeared at characteristic rates over the next 10 min at half-lives from 30 s to several minutes, with the most stable bands corresponding to those persisting under continuous stimulation. Control cells diluted into 200 nM folate behaved similarly to undiluted cells (Figures 3G and S3I). These results show that ErkB activity adapts much more slowly than many other responses to chemoattractant and that some ErkB

Figure 3. Phosphorylation of [S/T]PR Sites in Response to cAMP and Folate Depends on ErkB and Has Unusually Prolonged Kinetics

(A) Phospho-motif western of pTxR motif sequences following stimulation of undifferentiated Ax2 amoebae with 50 μM folate and aggregation competent Ax2 amoebae with 200 μM cAMP. See Figure S3 for buffer control. pTxR motif phosphorylation coincides with phosphorylation of ErkB (pMAPK). Tubulin shown as loading control.

(B) Temporal profiles of phosphorylation for individual substrate bands with chemoattractant-stimulated phosphorylation in (A). Gray lines show profile of individual bands, red and yellow lines show averages. Signal for individual bands normalized so that $T_0 = 0$, maximum phospho-signal = 1.

(C) TxR phosphorylation response (upper panels) to 50 μM folate treatment in chemotaxis signaling mutants and parent strains. Induction of TxR phosphorylation is lost in Gβ and Gα4 knockout cells, but background phosphorylation remains.

(D) Chemoattractant stimulation of pTxR motif phosphorylation is lost in *erkB⁻* but intact in *erkA⁻* mutants. Undifferentiated wild-type (Ax2), *erkB⁻*, and *erkA⁻* amoebae were stimulated with 50 μM folate. pTxR motif phosphorylation correlates with activity of ErkB (anti-pMAPK). Phosphorylation at HM motifs of PKB and PKBR1 and at a PKB substrate motif, is intact in *erkB⁻*. Tubulin loading control. See Figure S3 for cAMP stimulation experiments with *erkA⁻* and *erkB⁻* cells.

(E) High temporal-resolution quantification of induction of pTxR and ErkB (pMAPK) phosphorylation following chemoattractant stimulation. Left, undifferentiated amoebae stimulated with 50 μM folate. Right, cAMP-pulsed amoebae stimulated with 1 μM cAMP. Individual pTxR substrate bands shown in gray, average shown in blue. Quantification of ErkB phosphorylation (pMAPK) shown in red. Signals normalized as in (B). See Figure S3 for example blots.

(F) Phosphorylation profiles of pTxR phosphoproteins and of ErkB (pMAPK) during continuous saturating stimulation with chemoattractant. Left, undifferentiated amoebae stimulated with 500 μM folate. Right, cAMP-pulsed *acaA⁻* amoebae stimulated with 50 μM Sp-cAMPS. Gray lines show individual pTxR bands, blue shows average of pTxR band signals, and red shows pErkB signal. Signals normalized as in (B). See Figure S3 for example blots.

(G) Dephosphorylation profiles for individual pTxR phosphoproteins and for ErkB following stimulation of undifferentiated amoebae with 200 nM folate and subsequent dilution after 60 s to reduce folate concentration to 4 nM. Blue line shows normalized intensity of pTxR phospho-signal and red line shows pErkB. Vertical line shows time of folate dilution. Signal normalized as in (B).

See Figure S3 for example blot and constant folate control.

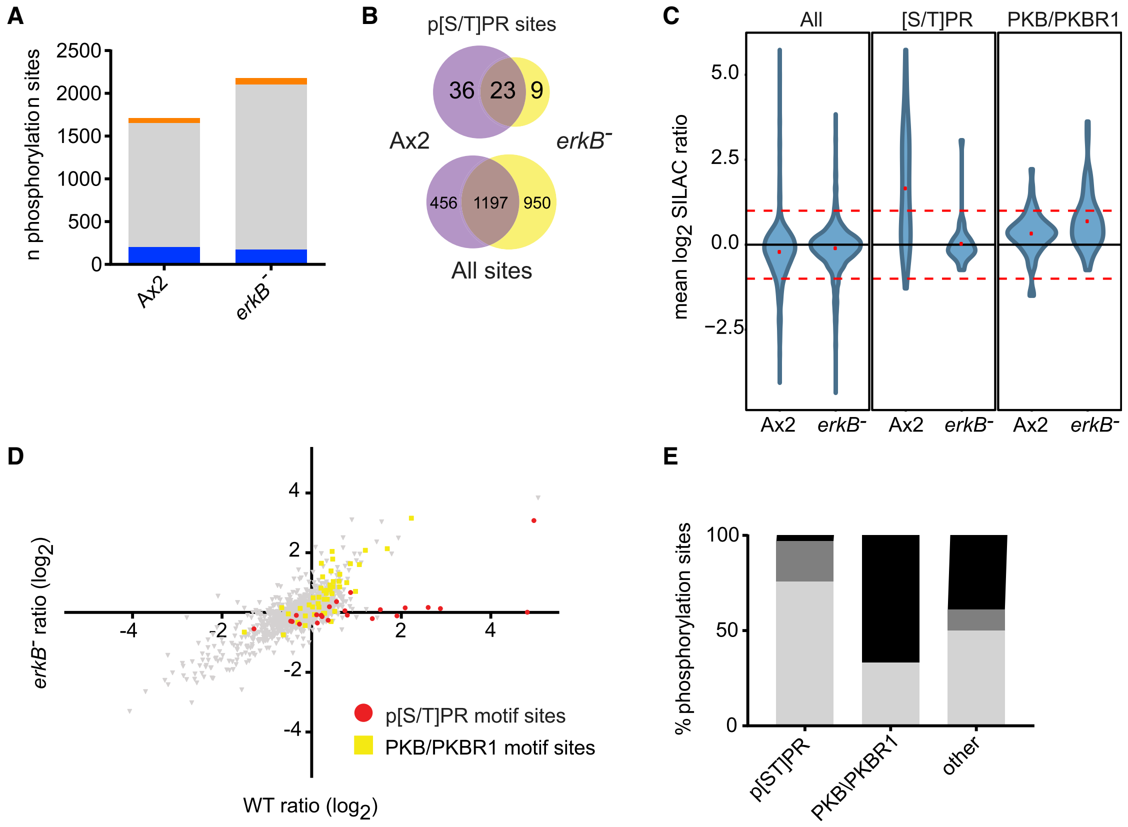

Figure 4. Phosphoproteomic Characterization of *erkB*⁻ Cells Confirms Link to p[S/T]PR Sites

(A) Comparison of phosphorylation sites identified in wild-type and *erkB*⁻ SILAC experiments either upregulated (>2-fold increase, orange), downregulated (>2-fold decrease, blue), or unchanged (gray), in response to 45 s folate stimulus. See Table S5 for data.

(B) Numbers of p[ST]PR phosphorylation sites identified in wild-type and *erkB*⁻ experiments. Fewer p[ST]PR sites are identified in *erkB*⁻ cells than in wild-type, a bias unexpected by chance based on the intersection of all sites identified in the two experiments.

(C) Distribution of SILAC ratios in wild-type and *erkB*⁻ cells following 45 s folate stimulation, for all sites, p[ST]PR sites, and PKB substrate sites.

(D) Scatterplot of all sites quantified in both wild-type and *erkB*⁻ experiments. PKB/PKBR1 substrate motif sites (yellow) and p[ST]PR motif sites (red) are highlighted. Most p[ST]PR sites with increased phosphorylation in wild-type show no phosphorylation response in *erkB*⁻ cells.

(E) Response in *erkB*⁻mutant of phosphorylation sites known to have increased phosphorylation after 45 s folate treatment in wild-type cells. Almost all sites matching the p[S/T]PR motif are either not detected (light gray bars) or do not respond (mid gray bars) to folate in the *erkB*⁻ mutant. By contrast other sites, including those matching the PKB/PKBR1 substrate motif, have largely intact responses to folate (black bars).

See Table S2 for details.

targets remain phosphorylated for prolonged periods provided chemoattractant is present. Differential phosphatase activity presumably accounts for the different rates of target dephosphorylation, providing an additional level of regulation.

To definitively test that [S/T]PR phosphorylations depend on ErkB, we employed SILAC. After stimulating *erkB*⁻ cells with folate for 45 s, 2,179 phosphorylation sites were identified (Figures 4A and 4B; Table S5). With only one exception, phosphorylation of the p[S/T]PR sites was not stimulated by folate in *erkB*⁻ mutants: either the phosphopeptide was not detected at all, or if it was, phosphorylation was not stimulated by folate (Figures 4C–4E). The exception, T627 of GefL, is presumably phosphorylated by a different kinase. We confirmed through the presence of other peptides that many of the ErkB target proteins are still expressed in *erkB*⁻ cells, showing loss of p[S/T]PR phosphorylation is not due to loss of the target protein (25/36 phosphorylations; Table S2). The overall stimulation for non [S/T]PR sites in wild-type and mutant experiments was similar. For example, PKB substrates show little difference between wild-type and mutants. Only p[S/T]PR sites showed a decreased response in *erkB*⁻ mutants (Figure 4D).

The p[S/T]PR Motif Is the Preferred Substrate for Direct Phosphorylation by ErkB

Phosphorylation of the p[S/T]PR motif clearly depends on ErkB, but it remained possible that phosphorylation is mediated by an intermediary kinase. To examine this possibility, we asked whether ErkB could directly phosphorylate such sites. ErkB was purified as a 6xHis-tagged fusion from *Escherichia coli*, giving a protein of the expected molecular weight but already phosphorylated, as indicated by an anti-phospho MAPK antibody and treatment with λ-phosphatase (Figure 5A). Purified ErkB was active in kinase assays against the myelin basic protein (MBP), a known MAPK substrate, as previously reported (Segall et al., 1995) but not against a GST control (Figures 5B and 5C).

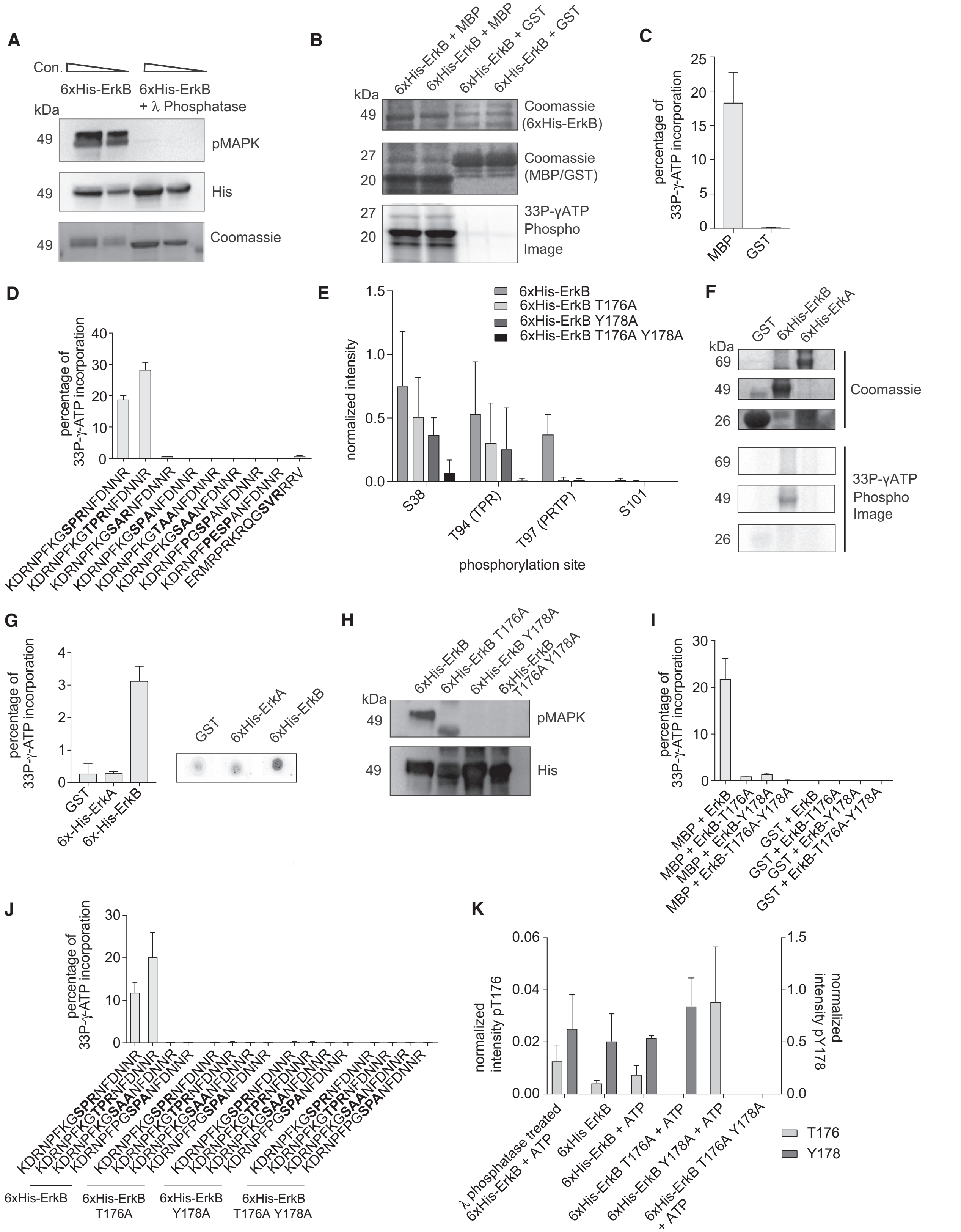

(legend on next page)

To what extent is ErkB kinase activity specific to the [S/T]PR motif? We examined ErkB specificity using a library of peptides based on a 17-amino acid peptide flanking S325 of EppA, a p[S/T]PR site identified previously (Chen and Segall, 2006) and in our SILAC experiments (Table S4). ErkB efficiently phosphorylated the wild-type peptide sequence and a peptide with the phospho-acceptor serine substituted to threonine (Figure 5D). Substitution of P+1 or R+2 with alanine effectively abolished phosphorylation, as did double replacement of P+1 and R+2 with alanine. A PKC-ε substrate peptide with arginine in the +2 position gave a trace of activity. Strikingly, two sequence variants matching the classical MAPK substrate consensus PXpSP were not phosphorylated by ErkB (Figure 5D).

To test ErkB activity on an intact protein, we determined the sites phosphorylated on MBP in our kinase assays, using mass spectrometry. MBP has 25 S and T residues available for phosphorylation, and the input protein was essentially un-phosphorylated. We observed strong phosphorylation at T94, which lies within a p[S/T]PR motif in MBP (Figure 5E). The S38 residue, in a different region of the protein, outside an identifiable consensus, was also phosphorylated, as was T97, which is within a classical MAPK motif and adjacent to T94. The T97 residue was only used when T94 was also phosphorylated (Figures 5E and S4A).

We noticed that the ErkA activation loop is not phosphorylated in *erkB*$^-$ mutants (Figure S4B) and that ErkA has an SPR motif close to its N terminus (Figure S4C), thus raising the possibility of cross-phosphorylation of ErkA by ErkB. The phosphorylated site was not detected *in vivo* by mass spectrometry—there is not sufficient lysine or arginine density to yield a suitable tryptic peptide. However, testing this idea *in vitro*, we found that His-tagged ErkA is clearly phosphorylated at this site by ErkB in kinase assays and not at the activation loop itself (Table S5). This experiment confirms, with a second protein, that the [S/T]PR motif is a preferred substrate for ErkB.

ErkB Activity Depends on Activation Loop Residues, which Are Targets for Autophosphorylation

Since ErkB expressed in *E. coli* is phosphorylated and active in kinase assays, it is possible that ErkB can autophosphorylate, as described for the mammalian atypical MAPK ERK8 (Klevernic et al., 2006). To test this directly, we dephosphorylated purified ErkB and incubated it with ATP to allow self-modification. As controls, we used GST and non-phosphorylated ErkA. ErkA is a classical Erk1/2 type MAPK (Figure S3B) and requires activation by the MAPKK MekA (Sobko et al., 2002), not present in the assay. Only ErkB could autophosphorylate. No phosphorylation of GST or ErkA was detected (Figures 5F and 5G).

To explore the mechanism of ErkB autophosphorylation, we mutated the activation loop residues (T176 and Y178) to alanine singly and in combination. Immunoblotting confirmed loss of pMAPK signal for all versions (Figure 5H). In kinase assays with MBP substrate, phosphorylation by single mutants of ErkB was reduced to 5%–7% of that by wild-type (Figure 5I and S4D). This residual activity was lost in the double phospho-null. Mass spectrometry showed that phosphorylation of MBP at T94 and T97 was at least 500-fold decreased in the double phospho-null mutant (Figure S4A). When tested against peptides, phosphorylation by single mutants was 1%–3% and by the double phospho-null mutant was 0.1%–0.5% of wild-type (Figure 5J). These results show that ErkB kinase activity depends on phosphorylation of the activation loop, with phosphorylation of both T176 and Y178 required for full activity.

Is autophosphorylation of T176 and Y178 sequential or independent? Mass spectrometry showed both T176 and Y178 were phosphorylated on ErkB expressed in bacteria (Figure 5K). Similarly, both sites were phosphorylated on ErkB after dephosphorylation followed by auto-rephosphorylation (Figure 5K). When we tested purified T176A and Y178A phospho-null mutants in the same analysis, we found the remaining

Figure 5. ErkB Directly and Specifically Phosphorylates p[S/T]PR

(A) 6xHis-tagged ErkB expressed and purified from *E. coli* is recognized by anti-pMAPK antibody on a western blot. Phosphatase-treatment results in loss of detection.

(B) 6xHis-ErkB is active in an *in vitro* P^{33}-incorporation kinase assay with myelin basic protein (MBP) as substrate. Proteins were separated on a gel. No kinase activity was observed with GST substrate.

(C) Quantification of 6xHis-ErkB kinase activity with MBP and GST substrates. P^{33}-incorporation was quantified with a scintillation counter. Error bars show SD, N = 3.

(D) Quantification of His-tagged ErkB kinase activity against peptide substrates. Sequence was modified to alter the phospho-acceptor residue, to substitute P+1 and R+2 residues to alanine, and to modify the flanking sequence to match classical MAPK substrate motifs. A known PKC-ε substrate peptide was also tested. P^{33}-incorporation was quantified using a scintillation counter. Error bars indicate SD, N = 3.

(E) Mass spectrometric analysis of MBP phosphorylated *in vitro* by wild-type and mutant His-tagged ErkB variants. The three most abundant phosphorylation sites are shown, along with S101, which is detected on the same peptide as T94 and T97. Phospho-site intensity was normalized such that the intensity of the most abundant phospho-site in each experimental replicate = 1. Bar chart shows mean of N = 3 experiments. Error bars indicate SD. See also Figures S4A and S4D.

(F) The ability of ErkB and ErkA to autophosphorylate was tested in an *in vitro* P^{33}-incorporation kinase assay. Proteins were separated on a gel for visualization. Autophosphorylation was only observed for ErkB, not ErkA. GST was used as a control.

(G) Quantification of autophosphorylation assay shown in (F). P^{33}-incorporation was quantified using a scintillation counter. Error bars indicate SD, N = 3. Samples of a representative assay were spotted on paper and developed for visualization.

(H) T176 and Y178 residues of the activation loop of His-tagged ErkB were mutated to alanine (T176A, Y178A, or both) and analyzed with a pMAPK antibody. In both single and double mutants phosphorylation is abolished.

(I) Kinase assay of wild-type and point mutant ErkB variants where activation loop threonine and tyrosine residues are mutated to alanine. P^{33}-incorporation was quantified using a scintillation counter. Error bars show SD, N = 3. See Figure S4D.

(J) Kinase assay of wild-type and point mutant His-tagged ErkB variants against peptide substrates. P^{33}-incorporation was quantified using a scintillation counter. Error bars show SD, N = 3.

(K) Mass spectrometric analysis of ErkB activation loop phosphorylation. Wild-type and activation loop variants were analyzed alongside wild-type ErkB in the absence of ATP, and phosphatase-treated wild-type ErkB subsequently re-phosphorylated post-purification. Phospho-site intensity was normalized such that the intensity of the most abundant phospho-site in each experimental replicate = 1. Bar chart shows mean of N = 3 experiments. Error bars indicate SD.

A

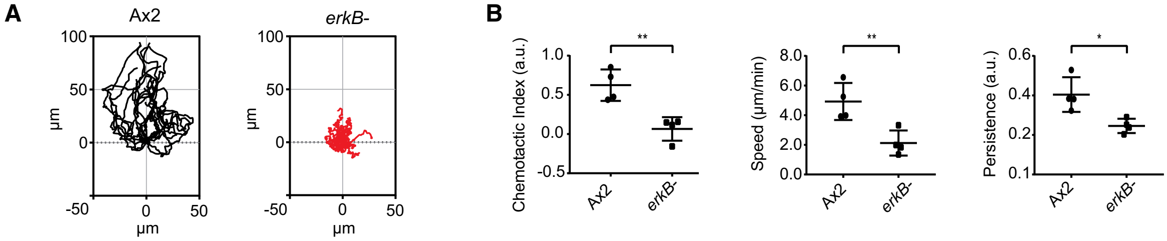

C

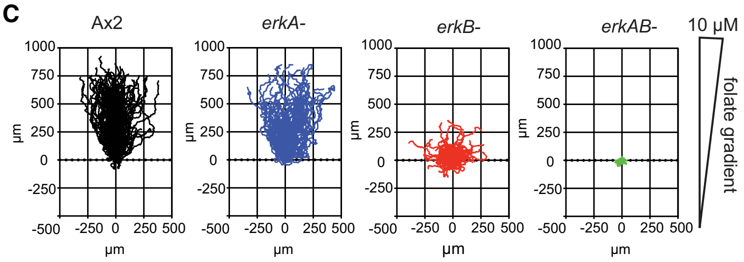

D

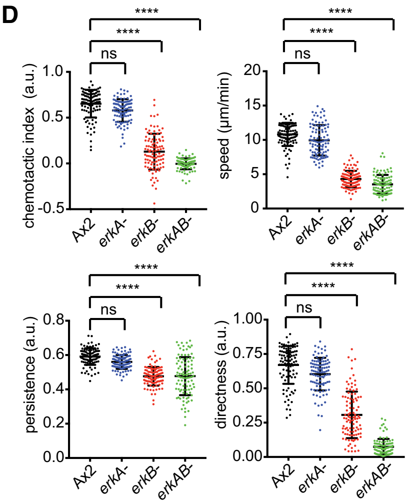

E

10 µM folate	Ax2	*erkA-*	*erkB-*	*erkAB-*
Chemotactic Index (a.u.)	0.652<br>SD ± 0.150	0.579 ns<br>SD ± 0.123	0.129 ****<br>SD ± 0.195	-0.004 ****<br>SD ± 0.058
Speed (µm/min)	10.800<br>SD ± 1.678	9.936 ns<br>SD ± 2.205	4.295 ****<br>SD ± 1.184	3.532 ****<br>SD ± 1.360
Persistence (a.u.)	0.591<br>SD ± 0.048	0.560 ns<br>SD ± 0.040	0.476 ****<br>SD ± 0.054	0.477 ****<br>SD ± 0.110
Directness (a.u.)	0.670<br>SD ± 0.137	0.603 ns<br>SD ± 0.118	0.307 ****<br>SD ± 0.169	0.074 ****<br>SD ± 0.057

F

folate	Ax2 (1µm)	*erkB-* (1µm)	Ax2 (0.1µm)	*erkB-* (0.1µm)
Chemotactic Index (a.u.)	0.658<br>SD ± 0.200	0.109 ****<br>SD ± 0.242	0.181<br>SD ± 0.222	0.080 ns<br>SD ± 0.245
Speed (µm/min)	12.426<br>SD ± 2.372	3.057 ****<br>SD ± 1.328	11.822<br>SD ± 3.240	3.879 ****<br>SD ± 1.567
Persistence (a.u.)	0.609<br>SD ± 0.058	0.454 ***<br>SD ± 0.143	0.505<br>SD ± 0.083	0.510 ns<br>SD ± 0.090
Directness (a.u.)	0.689<br>SD ± 0.186	0.305 ****<br>SD ± 0.182	0.310<br>SD ± 0.160	0.305 ns<br>SD ± 0.213

G

10 µM folate	Ax3	*erkB^{hyp}* AK240	*erkB^{hyp}* HS176
Chemotactic Index (a.u.)	0.622<br>SD ± 0.121	0.167 ****<br>SD ± 0.200	0.219 ****<br>SD ± 0.168
Speed (µm/min)	11.037<br>SD ± 1.370	6.199 ****<br>SD ± 2.212	6.461 ****<br>SD ± 1.634
Persistence (a.u.)	0.572<br>SD ± 0.045	0.526 *<br>SD ± 0.053	0.479 ***<br>SD ± 0.071
Directness (a.u.)	0.659<br>SD ± 0.122	0.240 ****<br>SD ± 0.172	0.265 ****<br>SD ± 0.145

H

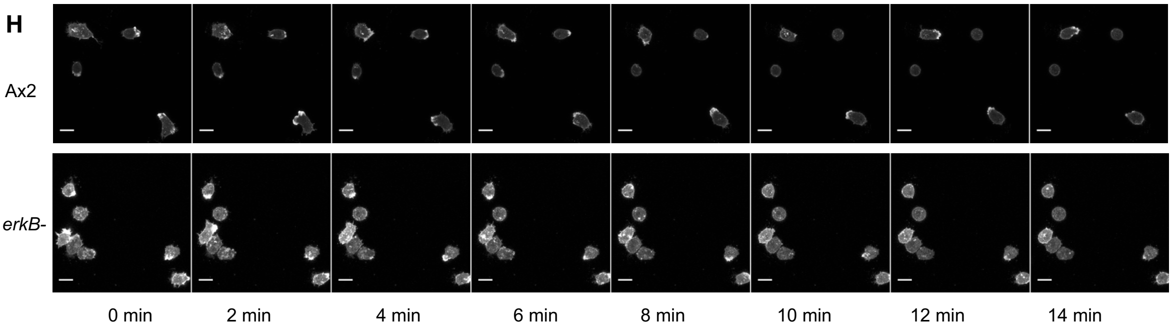

I

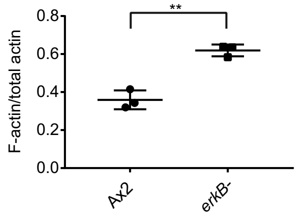

J

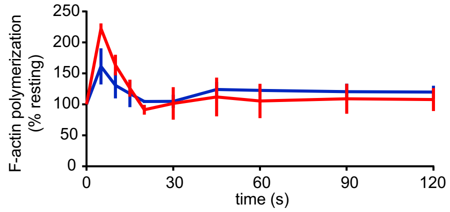

(legend on next page)

phosphorylatable residue was phosphorylated to a similar extent as the wild-type protein, suggesting phosphorylation of the activation loop residues is independent.

ErkB Is Essential for Chemotaxis and Efficient Movement

The large-scale modification of proteins by ErkB in response to chemoattractant implies it has an important role in chemotaxis. Chemotaxis to cAMP could not be tested because *erkB*$^-$ cells do not develop to the cAMP-responsive stage (Figure S3D). We therefore examined chemotaxis to folate by undifferentiated cells. Wild-type cells (grown on bacteria as a food source) chemotax well to a needle releasing folate. In contrast, ErkB null cells are strongly defective, moving much more slowly, with a chemotactic index close to zero (Figures 6A and 6B). For a more quantitative long-term analysis of chemotaxis in the ErkB mutant, we used an under-agarose chemotaxis assay using cells grown in liquid media. Amoebae under a layer of agarose containing a uniform concentration of folate locally degrade the folate to create a gradient of chemoattractant. This self-generated gradient moves with the wave of chemotaxing cells as they traverse long distances (Tweedy et al., 2016). *erkB*$^-$ cells had strong defects in cell speed, directness, and chemotactic index, as well as a minor defect in persistence (Figures 6C–6E). While these chemotaxis defects were not observed in *erkA*$^-$ mutants, *erkA*$^-$/*erkB*$^-$double mutants were even slower with reduced directness (Figures 6C–6E). *erkB*$^-$ mutants were strongly defective over a broad dynamic range of chemoattractant concentration (0.1 μM to 10 μM folate) (Figures 6F and S5A). Mixing fluorescently labeled wild-type and *erkB*$^-$cells in the same assay showed that chemotaxis defects of *erkB*$^-$ cells remained when in the presence of a folate gradient generated by wild-type cells, so they cannot be attributed to an inability of *erkB*$^-$ cells to degrade folate and establish a gradient (Figures S5D–S5G).

Chemotaxis defects are often inferred when cells are merely slow. To test whether slow movement was the root cause of the *erkB*$^-$ chemotaxis defects, we used *erkB* hypomorph strains (Segall et al., 1995) where residual pErkB could be detected, though at a much lower level than wild-type (Figure S5B). Hypomorph cells moved with nearly double the speed of *erkB*$^-$ cells but had directness, persistence, chemotaxis index, and developmental defects similar to those of the knockout mutant (Figures 6G, S5C, and S6H–S6I). Taken together, these data indicate that ErkB regulates both cell motility and chemotaxis over a large dynamic range of chemoattractant concentrations.

Do the defects in chemotaxis in the *erkB*$^-$ mutants reflect a specific chemotaxis defect, or a general inability of sick cells to perform complex functions? To evaluate this, we examined other cellular phenotypes. Both *erkB*$^-$ and *erkA*$^-$/*erkB*$^-$ cells grew robustly in liquid media with no defects in particle or fluid uptake (Figures S6C and S6D). Liquid-grown *erkB*$^-$ cells were of normal size, although double mutants were smaller (Figure S6E). Under agar chemotaxis experiments used cells grown in liquid culture, indicating a general cell welfare issue could not explain the strong chemotaxis defects of the *erkB*$^-$ mutants. Further supporting this conclusion, although *erkB*$^-$ cells form very small plaques on bacterial lawns, they do not have a generalized problem with actin-based protrusions, as they phagocytosed a range of different types and sizes of particles at wild-type rates (Figures S6F and S6G), despite reduced cell size (Figure S6J).

What cellular aspects of the response to chemoattractant are perturbed in ErkB nulls? Confocal imaging showed *erkB*$^-$ cells expressing an F-actin reporter make dynamic F-actin projections, but these are generally ineffective at moving the cell: cells formed local projections, but the rear remained anchored (Figure 6H; Video S1). *ErkB*$^-$ cells were also characterized by an abundance of transient F-actin accumulations on their basal surface. These are rare in wild-type cells (Figure S5H).

Polymerization assays showed actin is significantly over-assembled in ErkB null cells as measured by the proportion of F-actin to total actin (Figure 6I). Dynamic actin polymerization in response to chemotactic stimulation occurred with similar kinetics to wild-type, although the relative magnitude of the response is reduced in *erkB*$^-$ cells, reflecting higher resting levels of F-actin (Figures 6I and 6J).

Figure 6. *erkB*$^-$Mutant Cells Have Severe Defects in Motility and Chemotaxis

(A) Chemotaxis of undifferentiated amoebae toward a micropipette containing 100 μM folate. Cell tracks from a representative day's assay shown. Cells were filmed for 40 min and tracked until cells either left the field of view or stayed in close proximity to the micropipette for more than 4 consecutive frames. Cell tracks of at least 35 frames duration are shown.

(B) Chemotaxis parameters for the micropipette assay. Cell tracks used in analysis were ≥35 frames duration. 16–20 cells were tracked per strain per day. Points show mean values for all cells on each day, and lines show grand mean of N = 4 days. Error bars show SD. Statistics show Student's t test using N = 4, *$p \leq 0.05$, **$p \leq 0.01$.

(C) Chemotaxis of undifferentiated amoebae under agarose containing 10 μM folate. Individual cell tracks for Ax2, *erkB*$^-$, *erkA*$^-$, and *erkA*$^-$/*erkB*$^-$ are shown. Cells were tracked for 90 min. Of three experiments, N = 100 cells per cell line were analyzed.

(D) Chemotaxis parameters for under agarose assay shown in (C). Lines indicate means for all cell tracks. Error bars show SD. Statistics show Welch's t test (****$p \leq 0.0001$).

(E) Summary of chemotaxis parameters shown in (D). Values of mean and SD are shown. Statistics show Welch's t test (****$p \leq 0.0001$).

(F) Chemotaxis parameters for undifferentiated Ax2 and *erkB*$^-$ cells chemotaxing under agarose containing either 1 μM or 0.1 μM folate. Three experiments with N = 60 cells. Statistics show Welch's t tests (****$p \leq 0.0001$, ***$p \leq 0.001$). See also Figure S5.

(G) Chemotaxis parameters for undifferentiated Ax3 and *erkB*$^-$ hypomorphs AK240 and HS176 under agarose containing 10 μM folate. N = 100 cells from three experiments. See also Figure S5. Statistics show Welch's t tests (****$p \leq 0.0001$, *$p \leq 0.1$).

(H) Confocal timelapse of F-actin localization in Ax2 and *erkB*$^-$ undifferentiated amoebae assayed by lifeact:mCherry (see also Video S1). Images are maximum intensity projections. Scale bar, 10 μm. Images represent N = 2 independent imaging sessions. See also Figure S5.

(I) Resting F-actin levels in undifferentiated wild-type and *erkB*$^-$ cells expressed as fraction of total actin, as assayed by anti-actin immunoblot of extracted cytoskeletons and whole cell extracts. Mean of three experiments shown. Error bars show SD. Statistics show unpaired, two-tailed Student's t test **$p \leq 0.01$.

(J) Actin polymerization in undifferentiated Ax2 (red) and *erkB*$^-$ cells (blue) following uniform stimulation with 50 μM folate, assayed by binding of TRITC-phalloidin. F-actin levels expressed as percentage of resting levels for each strain. Error bars show SD; N = 3.

Candidate Regulators in the Core Chemotaxis Phosphoproteome Have Roles in Chemotaxis and Cell Motility

Our phosphoproteomics screen reveals many potential regulators of chemotaxis. We evaluated three of these: the RhoGAPs GacG and GacH and the histidine kinase DhkI (Table S4). *GacG*$^{-}$ cells formed expanded colony-feeding fronts on bacterial lawns (Figure S7D), usually indicative of aberrant motility. In folate chemotaxis assays, these cells moved much faster than wild-type, though with wild-type persistence and chemotactic index (Figures S7A and S7B). Undifferentiated *gac*G^{-} cells were more polarized than wild-type and accumulated F-actin at the leading edge (Figure S7C). Developed *gacH*$^{-}$ and *dhkI*$^{-}$ cells had defects in persistence in a 1 μM cAMP gradient. CsaA expression indicated wild-type developmental timing of *gacH*$^{-}$ and *dhkI*$^{-}$ cells showing that the chemotaxis defects observed are not the result of delayed development (Figures S7E and S7F). As might be expected for cells with defective cAMP chemotaxis, formation of multicellular aggregates was delayed in *gacH*$^{-}$ and *dhkI*$^{-}$ cells (Figure S7G). Thus, all tested candidates have a role in chemotaxis or cell movement.

DISCUSSION

We have identified a core set of 78 proteins that are rapidly phosphorylated in response to two different chemoattractants. Strikingly, the majority are phosphorylated at an [S/T]PR site by a single kinase, the MAP kinase, ErkB. This is shown both by the loss of these phosphorylations when ErkB is ablated and the specificity of ErkB in kinase assays. This places ErkB as the dominant protein kinase regulating the core chemotactic phosphoproteome, phosphorylating 52 of the 78 proteins, with the much-studied kinases PKB and PKBR1 accounting for only six.

This core phosphoproteome is highly enriched in proteins already implicated in cell movement and chemotaxis but contains a greater number of candidates whose role is not yet explored. All candidates evaluated showed significant roles in chemotaxis or motility, so we expect the remainder of the cohort will yield many more. The core set is enriched in signaling proteins including GEFs and GAPs for small G-proteins, protein kinases, and phosphatases. Surprisingly, there were only two clear cytoskeletal proteins, implying ErkB is a regulator of regulators in the signaling network, rather than a direct regulator of effectors. This ErkB target specificity potentially suggests that chemoattractants regulate the cytoskeleton by routes other than protein phosphorylation, perhaps through small G-proteins, whose regulators are recruited to the cytoskeleton by chemotactic stimulation (Sobczyk et al., 2014).

ErkB is a member of the conserved yet relatively unstudied MAPK15 clade of MAP kinases, along with mammalian ERK7 and ERK8 (Figure S3B). Neither the substrate specificity nor regulation of this clade is well understood. Our results, both *in vivo* and *in vitro*, show that ErkB strongly phosphorylates substrates at a [S/T]PR motif. This preference has not been described before for any kinase to our knowledge, but it is notable that ERK8 also phosphorylates a TPR site in MBP (Klevernic et al., 2006), suggesting a [S/T]PR substrate is a conserved feature of this group of MAP kinases. However, with over 1,000 [S/T]PR sequences in the *Dictyostelium* proteome, other interactions may also contribute to ErkB specificity.

Full activation of ErkB requires dual phosphorylation of the TXY motif in the activation loop. This phosphorylation is stimulated by signaling through the GPCRs for folate or cAMP. We have demonstrated that stimulation depends on both Gα and Gβ subunits of heterotrimeric G-proteins. We found ErkB strongly activates itself by autophosphorylation. Human ERK8 is proposed to self-activate by autophosphorylation without an upstream kinase (Klevernic et al., 2006), and we suggest a similar scenario for ErkB. If so, there may be no need for upstream activating kinases, with pathway regulation achieved by autophosphorylation and phosphatase activity (Brzostowski and Kimmel, 2006).

ErkB may control its own activity through feedback, as targets include the putative MAPK phosphatases MkpA and Mpl1 (Rodriguez et al., 2008), which are phosphorylated in response to cAMP and folate, respectively. There may also be cross-talk between ErkA and ErkB, since phosphorylation of the activation loop of ErkA in cells depends on ErkB, and ErkB phosphorylates ErkA at a remote [S/T]PR site *in vitro*. The consequences and functions of this phosphorylation remain to be explored.

ErkB null cells are defective in both speed of movement and chemotactic orientation in a variety of folate gradients (Schwebs et al., 2018), presumably reflecting cumulative effects of misregulation of target proteins. Null mutants in these targets present diverse movement phenotypes. Mutants of the RhoGAP, GacG, described here, chemotax nearly twice as fast as wild-type to folate but are almost incapable of fluid uptake by macropinocytosis (Williams et al., 2018). GacG is phosphorylated by PKB as well as ErkB (Tang et al., 2011) and could therefore integrate signaling via both pathways. The RhoGAP, GacH, and histidine kinase, DhkI, have subtle defects in cAMP chemotaxis, with DhkI possibly regulating intracellular cAMP levels through a phospho-relay to the cAMP phosphodiesterase, RegA (Thomason and Kay, 2000).

The common feature of regulation by ErkB is that phosphorylation of its target proteins is delayed after chemotactic stimulation, but prolonged in continuously stimulated cells, up to 30 min. This sets ErkB phosphorylations apart from other well-described signaling events, such as PIP3 or cGMP production, or activation of PKB and PKBR1 protein kinases, which are more rapid and adapt in the continued presence of ligand, returning to basal levels within minutes (Swaney et al., 2010).

This difference suggests that ErkB plays a distinct role in chemotaxis. We speculate ErkB may be required for slow adaptive responses to ambient levels of chemoattractant and for cellular memory of past levels. Adaptation extends the dynamic range of chemotaxis and is proposed to act at multiple points in chemoattractant signaling (Brzostowski et al., 2013; Liao et al., 2013). Memory allows aggregating cells, which experience waves of chemoattractant, to move in the front of the wave, but not reverse in the back (Skoge et al., 2014). Both require cells to stably modify signaling components when signal is present and for this modification to decay once signal is withdrawn, as is observed for ErkB phosphorylations.

In conclusion, this work changes our perception of chemotactic signal transduction in *Dictyostelium*, placing ErkB as a

dominant protein kinase, which acts on a core set of chemoattractant-stimulated targets. ErkB is regulated by heterotrimeric G-proteins and autophosphorylation and triggers persistent, non-adapting phosphorylations. ErkB is crucial for cell motility and chemotaxis, and its targets include regulators of chemotaxis and motility. Globally, our analysis implies the complexity of the chemotactic signaling network was greatly undersampled by traditional methods. We propose ErkB regulates multiple activities relating to the physical response of the cell, not by direct action on the cytoskeleton, but by coordinating a large panel of regulators of cytoskeletal dynamics.

STAR★METHODS

Detailed methods are provided in the online version of this paper and include the following:

- KEY RESOURCES TABLE
- CONTACT FOR REAGENT AND RESOURCE SHARING
- EXPERIMENTAL MODEL AND SUBJECT DETAILS
  - Growth of *Dictyostelium Discoideum*
  - SILAC Labeling of *Dictyostelium Discoideum*
  - SILAC Labeling of *E. coli*
- METHOD DETAILS
  - Stimulation of SILAC Labeled *D. Discoideum*
  - Sample Digest and Phosphopeptide Enrichment
  - Sample Digest for MS with Non-SILAC Samples
  - Mass Spectrometry Data Acquisition
  - Mass Spectrometry Data Processing
  - Classification of Phosphoproteomic Data
  - Testing Heavy Isotope Incorporation
  - Chemoattractant Treatment for Immunoblotting
  - Immunoblot Assays of Protein Phosphorylation
  - Expression of 6xHis Tagged Erk Proteins
  - *In Vitro* Kinase Assay
  - Generation of *Dictyostelium* Knockout Mutants
  - Growth on Bacterial Lawn
  - Uptake Assays
  - Cell Size
  - Developmental Time Course
  - Micropipette Chemotaxis Assay
  - Under Agarose Folate Chemotaxis Assay
  - Insall Chamber Chemotaxis Assay
  - F-Actin Polymerization Assays
  - Live-Cell Imaging of Actin Cytoskeleton
  - Phylogenetic Analysis of MAPK Sequences
- QUANTIFICATION AND STATISTICAL ANALYSIS
  - Gene Ontology Enrichment Analysis
  - Mutant Phenotype *In Silico* Analysis
  - Motif Discovery and Enrichment Analysis
  - Chemotaxis Assays
  - F-Actin Polymerization Quantification
- DATA AND SOFTWARE AVAILABILITY

SUPPLEMENTAL INFORMATION

Supplemental Information includes seven figures, six tables, and one video and can be found with this article online at https://doi.org/10.1016/j.devcel.2018.12.001.

ACKNOWLEDGMENTS

We thank Douwe Veltman and Emmanuel Derivery for plasmids; Peter Devreotes and lab for the gift of the *gpbA*$^{-}$ strain; the wider *Dictyostelium* community and Dicty Stock centre for strains; and members of the Kay laboratory for helpful discussion and strains. We are grateful to Clara Sidor and Roger Williams for help with *in vitro* kinase assays, and the light microscopy and flow cytometry facilities of the LMB for support. We thank Leo Hillier of the LMB Visual Aids department for graphic design work. We acknowledge the Medical Research Council for core funding: grant MC_U105115237 to R.R.K., and the Karn Fund for additional funding (J.M.E.N.). Work was supported by Wellcome Trust Senior Fellowship 202867/Z/16/Z to JRC, and MRC funding (MC_U12266B) to the MRC LMCB University Unit at UCL.

AUTHOR CONTRIBUTIONS

Conceptualization, J.M.E.N., P.P., E.S., and R.R.K.; Methodology, J.M.E.N., S.P.-C., P.P., L.T., and E.S.; Investigation, J.M.E.N., P.P., S.P.-C., and E.S.; Resources, J.M.E.N., P.P., T.D.W., L.T., and M.S.; Writing, J.M.E.N., P.P., J.R.C., and R.R.K.; Formal Analysis, J.M.E.N., P.P., and L.T.; Visualization, J.M.E.N. and P.P.; Supervision, M.S., E.S., J.R.C., and R.R.K.

DECLARATION OF INTERESTS

The authors declare no competing interests.

Received: August 1, 2017
Revised: October 15, 2018
Accepted: December 3, 2018
Published: January 3, 2019

REFERENCES

Artemenko, Y., Lampert, T.J., and Devreotes, P.N. (2014). Moving towards a paradigm: common mechanisms of chemotactic signaling in *Dictyostelium* and mammalian leukocytes. Cell. Mol. Life Sci. *71*, 3711–3747.

Basu, S., Fey, P., Jimenez-Morales, D., Dodson, R.J., and Chisholm, R.L. (2015). DictyBase 2015: expanding data and annotations in a new software environment. Genesis *53*, 523–534.

Brzostowski, J.A., and Kimmel, A.R. (2006). Nonadaptive regulation of ERK2 in *Dictyostelium*: implications for mechanisms of cAMP relay. Mol. Biol. Cell *17*, 4220–4227.

Brzostowski, J.A., Sawai, S., Rozov, O., Liao, X.-H., Imoto, D., Parent, C.A., and Kimmel, A.R. (2013). Phosphorylation of chemoattractant receptors regulates chemotaxis, actin reorganization and signal relay. J. Cell Sci. *126*, 4614–4626.

Caterina, M.J., Devreotes, P.N., Borleis, J., and Hereld, D. (1995). Agonist-induced loss of ligand binding is correlated with phosphorylation of cAR1, a G protein-coupled chemoattractant receptor from *Dictyostelium*. J. Biol. Chem. *270*, 8667–8672.

Charest, P.G., Shen, Z., Lakoduk, A., Sasaki, A.T., Briggs, S.P., and Firtel, R.A. (2010). A Ras signaling complex controls the RasC-TORC2 pathway and directed cell migration. Dev. Cell *18*, 737–749.

Chen, S., and Segall, J.E. (2006). EppA, a putative substrate of DdERK2, regulates cyclic AMP relay and chemotaxis in *Dictyostelium discoideum*. Eukaryot. Cell *5*, 1136–1146.

Cox, J., and Mann, M. (2008). MaxQuant enables high peptide identification rates, individualized p.p.b.-range mass accuracies and proteome-wide protein quantification. Nat. Biotechnol. *26*, 1367–1372.

Demšar, J., Curk, T., Erjavec, A., Gorup, C., Hocevar, T., Milutinovic, M., Možina, M., Polajnar, M., Toplak, M., Staric, A., et al. (2013). Orange: data mining toolbox in Python. J. Mach. Learn. Res. *14*, 2349–2353.

Dephoure, N., Zhou, C., Villén, J., Beausoleil, S.A., Bakalarski, C.E., Elledge, S.J., and Gygi, S.P. (2008). A quantitative atlas of mitotic phosphorylation. Proc. Natl. Acad. Sci. U S A *105*, 10762–10767.

Dereeper, A., Guignon, V., Blanc, G., Audic, S., Buffet, S., Chevenet, F., Dufayard, J.-F., Guindon, S., Lefort, V., Lescot, M., et al. (2008). Phylogeny.fr: robust phylogenetic analysis for the non-specialist. Nucleic Acids Res. *36*, W465–W469.

Fets, L., Nichols, J.E., and Kay, R.R. (2014). A PIP5 kinase essential for efficient chemotactic signaling. Curr. Biol. *24*, 415–421.

Gerisch, G., Fromm, H., Huesgen, A., and Wick, U. (1975). Control of cell-contact sites by cyclic AMP pulses in differentiating *Dictyostelium* cells. Nature *255*, 547–549.

Gnad, F., Gunawardena, J., and Mann, M. (2011). PHOSIDA 2011: the post-translational modification database. Nucleic Acids Res. *39*, D253–D260.

Graziano, B.R., and Weiner, O.D. (2014). Self-organization of protrusions and polarity during eukaryotic chemotaxis. Curr. Opin. Cell Biol. *30*, 60–67.

Hadwiger, J.A., Lee, S., and Firtel, R.A. (1994). The G alpha subunit G alpha 4 couples to pterin receptors and identifies a signaling pathway that is essential for multicellular development in *Dictyostelium*. Proc. Natl. Acad. Sci. U S A *91*, 10566–10570.

Hanke, S., Besir, H., Oesterhelt, D., and Mann, M. (2008). Absolute SILAC for accurate quantitation of proteins in complex mixtures down to the attomole level. J. Proteome Res. *7*, 1118–1130.

Hoeller, O., and Kay, R.R. (2007). Chemotaxis in the absence of PIP3 gradients. Curr. Biol. *17*, 813–817.

Jin, T., Xu, X., and Hereld, D. (2008). Chemotaxis, chemokine receptors and human disease. Cytokine *44*, 1–8.

Kamimura, Y., Xiong, Y., Iglesias, P.A., Hoeller, O., Bolourani, P., and Devreotes, P.N. (2008). PIP3-independent activation of TorC2 and PKB at the cell's leading edge mediates chemotaxis. Curr. Biol. *18*, 1034–1043.

Klevernic, I.V., Stafford, M.J., Morrice, N., Peggie, M., Morton, S., and Cohen, P. (2006). Characterization of the reversible phosphorylation and activation of ERK8. Biochem. J. *394*, 365–373.

Kortholt, A., Keizer-Gunnink, I., Kataria, R., and Van Haastert, P.J.M.V. (2013). Ras activation and symmetry breaking during *Dictyostelium* chemotaxis. J. Cell Sci. *126*, 4502–4513.

Lee, S., Parent, C.A., Insall, R., and Firtel, R.A. (1999). A novel Ras-interacting protein required for chemotaxis and cyclic adenosine monophosphate signal relay in *Dictyostelium*. Mol. Biol. Cell *10*, 2829–2845.

Liao, X.-H., Buggey, J., Lee, Y.K., and Kimmel, A.R. (2013). Chemoattractant stimulation of TORC2 is regulated by receptor/G protein-targeted inhibitory mechanisms that function upstream and independently of an essential GEF/Ras activation pathway in *Dictyostelium*. Mol. Biol. Cell *24*, 2146–2155.

Muinonen-Martin, A.J., Susanto, O., Zhang, Q., Smethurst, E., Faller, W.J., Veltman, D.M., Kalna, G., Lindsay, C., Bennett, D.C., Sansom, O.J., et al. (2014). Melanoma cells break down LPA to establish local gradients that drive chemotactic dispersal. PLoS Biol. *12*, e1001966.

O'Shea, J.P., Chou, M.F., Quader, S.A., Ryan, J.K., Church, G.M., and Schwartz, D. (2013). pLogo: a probabilistic approach to visualizing sequence motifs. Nat. Methods *10*, 1211–1212.

Olsen, J.V., Blagoev, B., Gnad, F., Macek, B., Kumar, C., Mortensen, P., and Mann, M. (2006). Global, in vivo, and site-specific phosphorylation dynamics in signaling networks. Cell *127*, 635–648.

Pan, M., Xu, X., Chen, Y., and Jin, T. (2016). Identification of a chemoattractant G-protein-coupled receptor for folic acid that controls both chemotaxis and phagocytosis. Dev. Cell *36*, 428–439.

Rodriguez, M., Kim, B., Lee, N.-S., Veeranki, S., and Kim, L. (2008). MPL1, a novel phosphatase with leucine-rich repeats, is essential for proper ERK2 phosphorylation and cell motility. Eukaryot. Cell *7*, 958–966.

Schindelin, J., Arganda-Carreras, I., Frise, E., Kaynig, V., Longair, M., Pietzsch, T., Preibisch, S., Rueden, C., Saalfeld, S., Schmid, B., et al. (2012). Fiji: an open-source platform for biological-image analysis. Nat. Methods *9*, 676–682.

Schwebs, D.J., Pan, M., Adhikari, N., Kuburich, N.A., Jin, T., and Hadwiger, J.A. (2018). *Dictyostelium* Erk2 is an atypical MAPK required for chemotaxis. Cell. Signal. *46*, 154–165.

Segall, J.E., Kuspa, A., Shaulsky, G., Ecke, M., Maeda, M., Gaskins, C., Firtel, R.A., and Loomis, W.F. (1995). A MAP kinase necessary for receptor-mediated activation of adenylyl cyclase in *Dictyostelium*. J. Cell Biol. *128*, 405–413.

Shaffer, B.M. (1975). Secretion of cyclic AMP induced by cyclic AMP in the cellular slime mould *Dictyostelium discoideum*. Nature *255*, 549–552.

Skoge, M., Yue, H., Erickstad, M., Bae, A., Levine, H., Groisman, A., Loomis, W.F., and Rappel, W.-J. (2014). Cellular memory in eukaryotic chemotaxis. Proc. Natl. Acad. Sci. U S A *111*, 14448–14453.

Sobczyk, G.J., Wang, J., and Weijer, C.J. (2014). SILAC-based proteomic quantification of chemoattractant-induced cytoskeleton dynamics on a second to minute timescale. Nat. Commun. *5*, 3319.

Sobko, A., Ma, H., and Firtel, R.A. (2002). Regulated SUMOylation and ubiquitination of DdMEK1 is required for proper chemotaxis. Dev. Cell *2*, 745–756.

Sugden, C., Urbaniak, M.D., Araki, T., and Williams, J.G. (2015). The *Dictyostelium* prestalk inducer differentiation inducing factor-1 (DIF-1) triggers unexpectedly complex global phosphorylation changes. Mol. Biol. Cell *26*, 805–820.

Swaney, K.F., Huang, C.-H., and Devreotes, P.N. (2010). Eukaryotic chemotaxis: a network of signaling pathways controls motility, directional sensing, and polarity. Annu. Rev. Biophys. *39*, 265–289.

Tang, L., Franca-Koh, J., Xiong, Y., Chen, M.-Y., Long, Y., Bickford, R.M., Knecht, D.A., Iglesias, P.A., and Devreotes, P.N. (2008). Tsunami, the *Dictyostelium* homolog of the Fused kinase, is required for polarization and chemotaxis. Genes Dev. *22*, 2278–2290.

Tang, M., Iijima, M., Kamimura, Y., Chen, L., Long, Y., and Devreotes, P. (2011). Disruption of PKB signaling restores polarity to cells lacking tumor suppressor PTEN. Mol. Biol. Cell *22*, 437–447.

Thomason, P., and Kay, R. (2000). Eukaryotic signal transduction via histidine-aspartate phosphorelay. J. Cell Sci. *113*, 3141–3150.

Town, C.D., Gross, J.D., and Kay, R.R. (1976). Cell differentiation without morphogenesis in *Dictyostelium discoideum*. Nature *262*, 717–719.

Tweedy, L., Knecht, D.A., Mackay, G.M., and Insall, R.H. (2016). Self-generated chemoattractant gradients: attractant depletion extends the range and robustness of chemotaxis. PLoS Biol. *14*, e1002404.

Ubersax, J.A., and Ferrell, J.E., Jr. (2007). Mechanisms of specificity in protein phosphorylation. Nat. Rev. Mol. Cell Biol. *8*, 530–541.

Williams, T.D., Paschke, P., Peak-Chew, S.-Y., and Kay, R.R. (2018). Akt kinases are required for efficient feeding by macropinocytosis in *Dictyostelium*. bioRxiv. https://doi.org/10.1101/417923.

Yan, J., Mihaylov, V., Xu, X., Brzostowski, J.A., Li, H., Liu, L., Veenstra, T.D., Parent, C.A., and Jin, T. (2012). A $G\beta\gamma$ effector, ElmoE, transduces GPCR signaling to the actin network during chemotaxis. Dev. Cell *22*, 92–103.

Zatulovskiy, E., Tyson, R., Bretschneider, T., and Kay, R.R. (2014). Bleb-driven chemotaxis of *Dictyostelium* cells. J. Cell Biol. *204*, 1027–1044.

STAR★METHODS

KEY RESOURCES TABLE

REAGENT or RESOURCE	SOURCE	IDENTIFIER
Antibodies		
anti phospho Thr-x-Arg	Cell Signaling Technology	Cat#2351; RRID: AB_331807
anti phospho p44/42 MAPK (Thr202/Tyr204)	Cell Signaling Technology	Cat#9101; RRID: AB_331646
anti phospho (S/T) Akt substrates (110B7E)	Cell Signaling Technology	Cat#9614; RRID: AB_331810
anti phospho p70 S6 kinase Thr389 (1A5)	Cell Signaling Technology	Cat#9206; RRID: AB_331790
anti CsA (123-353-1)	Developmental Studies Hybridoma Bank	Cat#123-353-1; RRID: AB_1553681
anti alpha tubulin (DM1A)	Cell Signaling Technology	Cat#3873; RRID: AB_1904178
anti actin	Sigma	Cat#A2066; RRID: AB_476693
anti-polyHistidine–Peroxidase	Sigma	Cat#A7058; RRID: AB_258326
anti rabbit IgG HRP conjugated	Bio-Rad	Cat#172-1019; RRID: AB_11125143
anti mouse IgG HRP conjugated	Bio-Rad	Cat#172-1011; RRID: AB_11125936
Bacterial and Virus Strains		
Klebsiella aerogenes		N/A
Escherichia coli AT713	Coli Genetic Stock Center	CGSC: #4529
Escherichia coli BL21-CodonPlus (DE3)-RIL competent cells	Agilent	Cat#230245
Escherichia coli xl10 gold	Agilent	Cat#200314
Chemicals, Peptides, and Recombinant Proteins		
Sp-cAMPS	Sigma	Cat#A166
TRITC-Phalloidin	Sigma	Cat#P1951
ATP, 10 mM	NEB	Cat#P0756S
SIH medium without Arg without Lys	Formedium	Cat#SIH1001
L-Arginine:HCl U-^{13}C6 99%, U-^{15}N4 99%	Cambridge Isotope Laboratories	Cat#CNLM-539-H
L-Lysine:2HCl U-^{13}C6 99% U-^{15}N2 99%	Cambridge Isotope Laboratories	Cat#CNLM-291-H
L-Arginine:HCl	Sigma	Cat#A6969
L-Lysine:HCl	Sigma	Cat#L8662
[γ-P33] Adenosine 5'-triphosphate (ATP)	Hartmann Analytic	Cat#SRF-301
Peptides	Biomatik	Custom made
MBP, bovine, purified, dephosphorylated	Merck	Cat#13-110
Experimental Models: Organisms/Strains		
Dictyostelium discoideum Ax2	Kay lab	Dictybase: DBS0235521
Dictyostelium discoideum Ax2 *act5*::GFP	Kay lab	HM1932
Dictyostelium discoideum Ax3	Dicty stock center	Dictybase: DBS0235542
Dictyostelium discoideum Ax4	Dicty stock center	Dictybase: DBS0235551
Dictyostelium discoideum JH10	Dicty stock center	Dictybase: DBS0236446
Dictyostelium discoideum erkB$^-$ (Ax2)	This paper	HM1734
Dictyostelium discoideum erkB$^-$- (Ax2) *act5*::mCherry	This paper	HM1995
Dictyostelium discoideum erkA$^-$ (Ax2)	This paper	HM1733
Dictyostelium discoideum erkAB$^-$ (Ax2)	This paper	HM1940
Dictyostelium discoideum gacG$^-$ (Ax2)	Williams et al. (2018)	HM1944
Dictyostelium discoideum gacH$^-$ (Ax2)	Williams et al. (2018)	HM1956
Dictyostelium discoideum dhkI$^-$ (Ax2)	This paper	HM1696
Dictyostelium discoideum pkbA$^-$*/pkgB*$^-$ (Ax3)	Peter Devreotes (Kamimura et al., 2008)	Dictybase: DBS0304640
Dictyostelium discoideum piaA$^-$ (Ax2)	Kay lab (Zatulovskiy et al., 2014)	HM1461

(Continued on next page)

Continued

REAGENT or RESOURCE	SOURCE	IDENTIFIER
Dictyostelium discoideum pikA⁻/pikB⁻/pikC⁻/pikF⁻ /pikG⁻ (Ax2)	Kay lab (Hoeller and Kay, 2007)	Dictybase: DBS0252652
Dictyostelium discoideum gpaD⁻ (JH8)	Dicty stock center	Dictybase: DBS0235984
Dictyostelium discoideum gpbA⁻ (Ax2)	Peter Devreotes	N/A
Dictyostelium discoideum erkB⁻ REMI AK240 (HL330)	Dicty stock center	Dictybase: DBS0235464
Dictyostelium discoideum erkB⁻ recapitulated REMI HS176 (HL330)	Dicty stock center	Dictybase: DBS0236385
Dictyostelium discoideum acaA⁻ (Ax2)	Kay lab (Zatulovskiy et al., 2014)	HM1366
Oligonucleotides		
See Table S6 for details of oligonucleotides used in this study		N/A
Recombinant DNA		
pDM1209 expression vector	Douwe Veltman	Addgene: #108986
pDM344 shuttle vector	Douwe Veltman	Dictybase: plasmid Cat#551
pDM1081	Douwe Veltman	Addgene: #108981
pDM1488	Douwe Veltman	Addgene: #108995
pDM1514	Douwe Veltman	Addgene: #108999
pDM1398 expression vector containing PH domain of PkgE	Douwe Veltman	N/A
pPI161: pDM344 shuttle vector containing lifeact:mCherry	This paper	N/A
pPI304: expression vector containing Lifeact:mCherry and PH-PkgE:GFP	Peggy Paschke	Addgene: #113232
pPI214: erkB KO plasmid	This paper	N/A
pKOSG-IBA-Dicty1 stargate acceptor KO plasmid	IBA life sciences	pKOSG-IBA-Dicty1
pGex2T	Sigma	GE28-9546-53
pRSET His SS TEV FA blue	Emmanuel Derivery	N/A
pPI486: bacterial expression plasmid 6xHis-ErkB	This paper	N/A
pPI528: bacterial expression plasmid 6xHis-ErkB T176A-Y178A	This paper	N/A
pPI544: bacterial expression plasmid 6xHis-ErkB T176A	This paper	N/A
pPI560: bacterial expression plasmid 6xHis-ErkB Y178A	This paper	N/A
pPI580: bacterial expression plasmid 6xHis-ErkA	This paper	N/A
Software and Algorithms		
Maxquant v1.0.13.13	Cox and Mann (2008)	http://www.biochem.mpg.de/5111795/maxquant
MASCOT v2.2	Matrix Science	http://www.matrixscience.com/
Scaffold 4	Proteome Software	http://www.proteomesoftware.com/
Strawberry Perl v5.22	Open source	http://strawberryperl.com/
PHOSIDA	Gnad et al. (2011)	http://www.phosida.com/
pLogo	O'Shea et al. (2013)	https://plogo.uconn.edu/
FIJI ImageJ	Schindelin et al. (2012)	https://fiji.sc/
Chemotaxis and Migration tool	Ibidi	http://ibidi.com/
Orange Data-mining Toolbox	Demšar et al. (2013)	https://orange.biolab.si/
Graphpad Prism v6 and v7	Graphpad Software	https://www.graphpad.com/
FlowJo	FlowJo LLC	https://www.flowjo.com/
Phylogeny.fr	Dereeper et al. (2008)	http://www.phylogeny.fr/

CONTACT FOR REAGENT AND RESOURCE SHARING

Further information and requests for resources and reagents should be directed to and will be fulfilled by the Lead Contact, John Nichols (j.nichols@ucl.ac.uk).

EXPERIMENTAL MODEL AND SUBJECT DETAILS

Growth of *Dictyostelium Discoideum*

Wild-type and mutant *D. discoideum* amoebae (all mating type 1) were grown at 22°C, either on a surface or in shaken suspension (180 rpm) in HL5 (Formedium) with 100 μg/ml dihydrostreptomycin, or in association with *Klebsiella aerogenes* on SM agar. For chemoattractant-stimulation immunoblot experiments cells were grown either in shaken suspension with HL5, or on clearing plates with *K. aerogenes* on SM agar. For needle chemotaxis assays, F-actin polymerization assays and immunoblot assays of resting F-actin, cells were grown on clearing plates with *K. aerogenes* on SM agar. Under agar chemotaxis experiments were performed with cells grown on petri dishes supplemented with HL5. For confocal imaging experiments, cells were grown in SorMC buffer (2 mM Na_2HPO_4, 15 mM KH_2PO_4, 50 μM $CaCl_2$, 50 μM $MgCl_2$) with *K. aerogenes* at OD_{600} = 2, supplemented with Geneticin (20 μg/ml).

SILAC Labeling of *Dictyostelium Discoideum*

Wild-type or *erkB*$^-$ amoebae were SILAC-labelled by shaken suspension growth (180rpm, 22°C) for 5-6 generations in either defined liquid medium (SIH minus arginine and lysine [Formedium, SIH1002], 3 mM heavy arginine [L-Arginine:HCl U-^{13}C6 99%, U-^{15}N4 99% Cambridge Isotope Laboratories], 4.5mM heavy lysine [L-Lysine:2HCl U-^{13}C6 99% U-^{15}N2 99% Cambridge Isotope Laboratories], 100 μg/ml dihydrostreptomycin), or in heat-killed SILAC-labelled *E. coli* (strain AT713; arginine and lysine auxotroph) in KK_2-MC (KK_2 [16.5 mM KH_2PO_4, 3.9 mM K_2HPO_4], 2 mM $MgSO_4$, 0.1 mM $CaCl_2$) supplemented with 60 mM proline, 100 μg/ml dihydrostreptomycin, 100μg/ml ampicillin. For unlabelled amoebae, cells were grown for the same period by the same methods, with heavy isotope arginine and lysine substituted with standard L-arginine (Sigma A6969) and L-lysine (Sigma L8662) at the same concentrations.

SILAC Labeling of *E. coli*

E. coli strain AT713 (Coli Genetic Stock Center #4529: *glnX44*(AS), λ$^-$, *cysJ43*, *argA21*, *lysA22*, *rpsL104*, *malT1*(λ^R), *xyl-7*, *mtlA2*, *thiE1*), an arginine and lysine auxotroph used previously for SILAC labelling (Hanke et al., 2008), was used as a food source to SILAC label *Dictyostelium* amoebae. For SILAC-labelling of *E.coli*, an M9 medium base was supplemented with glucose and amino acids (M9 minimal salts base, 2% glucose, 2 mM $MgSO_4$, 0.1 mM $CaCl_2$, 100 μg/ml each amino acids except arginine, lysine and proline, 200 μg/ml proline, 3 mM arginine, 4.5 mM lysine, 100 μg/ml, 100 μg/ml dihydrostreptomycin). Cultures of both unlabelled and labelled cells were prepared (see *SILAC labelling and chemoattractant stimulation of amoebae* for details of unlabelled and labelled arginine and lysine). An LB starter culture of AT713 was washed in M9 minimal salts, before being used to inoculate a 5 ml SILAC starter culture (both light and heavy forms). Parallel cultures lacking either arginine, lysine or both were used to check auxotrophic requirement of the strain. After overnight growth, the starter cultures were used 1 in 1000 to inoculate larger SILAC growth cultures which were grown to OD 1.8 before harvesting by centrifugation and four washes with KK_2. Aliquots of cell pellets were frozen on dry ice and stored at -20°C until use.

METHOD DETAILS

Stimulation of SILAC Labeled *D. Discoideum*

For cAMP stimulation, labelled and unlabelled cells were washed in KK_2-MC and resuspended at 2x10^7 cells/ml. Cells were starved in shaken suspension for 1 hour then pulsed with 90 nM cAMP every 6 minutes for 5 hours. Cells were then washed 3x in KK_2-MC and resuspended in KK_2 + 5 mM caffeine for 30 minutes to basalate cAMP responses in shaken suspension. Cells were washed twice in KK_2-MC at 4°C before being resuspended in 15°C KK_2-MC at 1.5x10^7 cells/ml. At 15°C, cells were added to 1/40th volume of either concentrated cAMP in KK_2-MC (heavy-labelled cells) or KK_2-MC alone (unlabelled cells). Final concentration of cAMP for 10 s and 45 s stimulation was 1 μM, for 360 s stimulation 200 μM. Following the desired stimulation time, an equal volume of TCA buffer (20% TCA + 5 mM sodium pyrophosphate + 2 mM β-glycerophosphate) was added to lyse cells and denature protein. Protein was pelleted by centrifugation at 4°C and washed twice in ice-cold acetone before being resuspended in urea buffer (8 M urea, 20 mM Hepes pH 8, 1 mM β-glycerophosphate, 2.5 mM sodium pyrophosphate, 1 mM sodium orthovanadate). Resuspended pellets from labelled and unlabelled samples were combined.

For folate stimulation, labelled cells and unlabelled cells were washed in KK_2-MC, before being resuspended at 2x10^{7}cells/ml in KK_2-MC in shaken suspension for 20 minutes before being washed twice in KK_2-MC and resuspended at 1.5x10^7 cells/ml in 15°C KK_2-MC. At 15°C cells were then added to 1/40th volume of either concentrated folate in KK_2-MC (heavy-labelled cells) or KK_2-MC alone (unlabelled cells). Final concentration of folate was 50 μM for 45 s stimulation, 200 μM for 360 s stimulation. Following desired stimulation time, an equal volume of TCA buffer was added and samples handled as for cAMP treatments.

Sample Digest and Phosphopeptide Enrichment

Protein samples were digested with trypsin as described previously (Dephoure et al., 2008) with some modifications. Briefly, protein samples in urea buffer (see above) were reduced with 5 mM DTT at 56°C for 30 min and alkylated with 10 mM iodoacetamide in the

dark at room temperature for 30 min. Then, the samples were digested with 400:1ratio (w/w) of Lys-C (Mass spectrometry grade, Promega) for 4hr at 25°C. Next, the samples were diluted from 8M to 2 M urea with 20 mM Hepes (pH 8.0) and were digested with 100:1 ratio (w/w) of trypsin (Promega) at 25°C for 17 hr. Digestion was stopped by the addition of trifluoroacetic acid (TFA) to a final concentration of 1%. Any precipitates were removed by centrifugation at 4200 rpm for 20 min. The supernatants were desalted using Sep-Pak Plus Short tC18 cartridges (Waters). Bound peptides were eluted with 60% acetonitrile in 0.5% acetic acid and lyophilized.

Strong cation exchange (SCX) chromatography was performed using an Ultimate 3000 Nano/Capillary LC System (Dionex). Dried peptides were dissolved in 200 μL of solvent A (5 mM $KHPO_4$ pH 2.65, 30% MeCN) and loaded onto a PolySULFOETHYL A column, 4.6 mm x 204 mm (PolyLC). The peptides were separated using a 45 min linear gradient of 0-23% solvent B (solvent A + 350 mM KCl) at a flow rate of 1 ml/min. Fifteen fractions, in 3 min intervals, were collected and lyophilized. Each fraction was resuspended in 0.1% TFA and desalted using home-made Poros R3 reversed phase (Applied Biosystems) column. The eluted peptides were lyophilized prior to TiO2 enrichment.

Phosphopeptides were enriched using TiO_2 titansphere-chromatography (GL Science Inc. Japan). Lyophilized peptides were resuspended in a solution which contained 2, 4-dihydroxybenzoic acid in 80% MeCN, 2% TFA (loading buffer) and incubated for 15 min with TiO_2 beads that were prewashed in the same solution. Beads were packed on top of a C8 stage tip (P10) and washed sequentially with loading buffer and 80 % MeCN, 2%TFA. Phosphopeptides were eluted with 40ul ammonia solution, pH 10.5, followed by 20 μl 50% MeCN, 0.05% TFA, and partially dried down using a SpeedVac. Samples were then acidified by addition of 4% formic acid and desalted with a C18 Stage tip that contained 1.5 μl of Poros R3 (Applied Biosystems) resin. Bound peptides were eluted with 30% MeCN, 4% FA and 80% MeCN, 4% FA. This solution was then partially dried down with SpeedVac and ready for mass spectrometry analysis.

In parallel, phosphopeptides were enriched using Phos-Select IMAC resin (Sigma). Desalted peptides were resuspended in 30% MeCN, 0.25 M acetic acid (loading solution) and 15-30 μl of IMAC beads, previously equilibrated with the loading solution was added. After 45 min incubation at room temperature, beads were transferred to C8 stage tip and washed 3 times with loading solution. Phosphopeptides were eluted sequentially with 40 μl of 0.4 M NH_3 and 20 μl of 50% MeCN, 4% FA. Phosphopeptides solution was acidified, partially dried, and desalted with a C18 Stage tip that contained 1.5 μl of Poros R3 resin. This solution was then partially dried and ready for mass spectrometry analysis.

Sample Digest for MS with Non-SILAC Samples

Protein samples, 5 μg in 20 mM HEPES, were reduced and alkylated as above. They were then digested with either trypsin, Lys-C or elastase (Promega). A small aliquot of each desalted sample was used for LC MS MS protein identification and the rest of the samples were used for IMAC phosphopeptide enrichment, as detailed above.

Mass Spectrometry Data Acquisition

Liquid chromatography was performed on a fully automated Ultimate U3000 Nano LC System (Dionex) fitted with a 100 μm x 2 cm PepMap100 C_{18} nano trap column and a 75 μm×25 cm reverse phase PepMap100 C_{18} nano column (Dionex).

Samples were separated using a binary gradient consisting of buffer A (2% MeCN, 0.1% formic acid) and buffer B (90% MeCN, 0.1% formic acid). Peptides were eluted with a linear gradient from 3 to 30% buffer B with a flow rate 250 nl/min. The outlet of the nano column was directly interfaced via a nanospray ion source to a LTQ Orbitrap Velos (all SILAC samples) or a Q Exactive Plus (QE, only the non-SILAC labelled samples) mass spectrometer (Thermo Scientific). The mass spectrometers were operated in standard data dependent mode, performed survey full-scan (m/z = 350-1600) in the Orbitrap analyzer, with a resolution of 60,000 at m/z =400 for Velos (70,000 at m/z=200 for QE), followed by MS2 acquisitions of the 20 most intense ions in the LTQ ion trap (15 for QE in orbitrap). FTMS target values of 1e6 and ion trap MSn target values of 1e4 (1e5 for QE) were used. Maximum FTMS scan accumulation times were set at 250 ms (50 ms for QE) and maximum ion trap MSn scan accumulation times were set at 200 ms (100 ms for QE in orbitrap). The Orbitrap measurements were internally calibrated using the lock mass of polydimethylcyclosiloxane at m/z 445.120025. Dynamic exclusion was enabled with a repeat count of 1, exclusion list of 500 and exclusion duration of 30 s.

Mass Spectrometry Data Processing

Raw files were processed using MaxQuant version 1.0.13.13 (http://www.biochem.mpg.de/5111795/maxquant) with Mascot (version 2.2, Matrix Science) as search engine. For all data sets, the default parameters in MaxQuant were used, except MS/MS tolerance at 0.6 Da and SILAC ratios calculations were performed on razor and unique peptides with a ratio count of one. Carbamidomethylation of cysteine was set as a fixed modification and oxidation of methionines, N-terminal protein acetylation, N-pyroglutamate and phosphorylation of STY as variable modifications. MaxQuant processed data was searched against the *Dictyostelium discoideum* proteome FASTA database ((Basu et al., 2015); downloaded from dictyBase April 2013). For ErkA *in vitro* phosphorylation MS experiment, data was analysed in Scaffold (Proteome Software).

Classification of Phosphoproteomic Data

Phosphorylation sites were classified by data quality and replication. Class I phosphorylation sites have a localisation probability ≥0.75 in at least one biological replicate and a H/L count of ≥2 in ≥2 biological replicates. Class II sites have a localisation probability ≥0.75 in at least one biological replicate and a H/L count ≥2 in only one biological replicate. Class III sites

have a localisation probability of ≥0.75 in at least one biological replicate and H/L counts all <2. Class IV sites have a localisation probability of ≥ 0.75 in at least one biological replicate but were not assigned any H/L ratio. Class V sites have localisation probability of <0.75 and cannot confidently be assigned to a single amino acid. Only Class I sites were used for downstream analysis. The H/L ratio reported for individual biological replicates is the median value of all H/L ratios measured. The mean value of this reported H/L ratio for all biological replicates was used in further analyses. Biological replicate n for SILAC experiments: 10s cAMP n=3, 45 s cAMP n=3, 360 s cAMP n=2, 45 s folate n=3, 360 s folate n=2, *erkB*⁻ 45 s folate n=2.

Testing Heavy Isotope Incorporation

To determine the incorporation efficiency of heavy amino acids in SILAC-labelled *E. coli* and *D. discoideum*, lysates from heavy labelled cells were separated on 1D-SDS PAGE gel, digested with trypsin and analyzed by LC-MS/MS. Raw files were processed using MaxQuant (version 1.0.13.13) with the re-quantify function not selected. The output peptides.txt files were used to calculate the rate of K and R incorporations. The incorporation efficiency for both lysine and arginine was >95%.

Chemoattractant Treatment for Immunoblotting

Cells were grown either in axenic shaken suspension in HL5 (Formedium) with 100 μg/ml dihydrostreptomycin, or as clearing plates with *Klebsiella aerogenes* on SM agar. Cells were harvested and washed in KK_2-MC.

For cAMP and Sp-cAMPS stimulations, cells were prepared as for cAMP stimulation in SILAC experiments, with cAMP pulsing for 4 hours duration. In Sp-cAMPS stimulations, *acaA*⁻ cells were used to avoid the effects of endogenous cAMP release. For folate stimulations, cells were prepared as for folate stimulation in SILAC experiments.

Cells were resuspended at $2x10^7$ cells/ml with aliquots added to $1/40^{th}$ volume of concentrated chemoattractant in KK_2-MC or, for T_0 and buffer control samples, KK_2-MC. After desired stimulation time, cells were lysed by addition of ½ volume of 3x LDS sample buffer (Invitrogen) supplemented with 2-mercaptoethanol, protease inhibitors (Roche complete) and phosphatase inhibitors (β-glycerophosphate, sodium pyrophosphate, sodium orthovanadate) and vortexing. For long-term chemoattractant stimulations, cells at $2x10^7$ cells/ml were shaken during stimulation with chemoattractant, with aliquots taken for lysis at different time points. For chemoattractant dilution experiments, shaken cells were stimulated with chemoattractant, with aliquots taken at different time-points pre– and post-dilution with either KK_2-MC or KK_2-MC containing the original concentration of chemoattractant. Samples of equal cell number were lysed by addition of equal volume 20% TCA and precipitated protein collected by centrifugation before resuspension in 1x LDS sample buffer with 2-mercaptoethanol, protease inhibitors and phosphatase inhibitors. For CsaA expression experiments, cells were prepared and pulsed with cAMP as for cAMP SILAC experiments, with samples taken at hourly intervals over a total of 4 or 7 hours pulsing.

For assaying regulation of pTxR phosphorylation and ErkB phosphorylation following folate stimulation; *erkB*⁻, *erkA*⁻, *erkAB*⁻, *piaA*⁻, *gpbA*⁻ and *pikA*⁻/*pikB*⁻/*pikC*⁻/*pikF*⁻/*pikG*⁻ cells were compared with the parental strain Ax2. *pkbA*⁻/*pkgB*⁻ cells were compared with the parental strain Ax3. *gpaD*⁻ cells were compared with JH10 wild-type cells. Stimulation of ErkB phosphorylation in the *erkB*⁻ REMI mutant strain AK240 was compared with Ax3 wild-type cells (from which the parental strain HL330 was previously derived).

Immunoblot Assays of Protein Phosphorylation

Cell lysate samples were boiled and separated by SDS-PAGE on 4-12% Bis-Tris gels before blotting onto PVDF membrane. Immunoblotting was carried out by standard procedures: membranes were blocked with 5% (w/v) BSA in TBS-T (50 mM Tris-Cl, 150 mM NaCl, 0.1% (v/v) Tween-20) for 1 hour at room temperature, antibodies were diluted in TBS-T + 5% BSA (w/v), wash steps were conducted with TBS-T. Primary antibodies were incubated overnight at 4°C Secondary antibodies (anti-rabbit IgG HRP/anti-mouse IgG HRP) were incubated for 1 hour at room temperature. Chemiluminescent detection was conducted using ECL (GE healthcare). Quantification of western blots was conducted using LI-COR Image Studio Lite v5.2.5. Band intensity for individual phosphoproteins was normalised to zero in t = 0 sample and 100 in sample with maximum band intensity.

To detect a subset of p[S/T]PR motif phosphorylations, an anti-pTxR primary antibody was used. Activation loop phosphorylation of ErkB was detected with anti phospho p44/42 MAPK (Thr202/Tyr204). Phospho-substrates of PKB and PKBR1 were detected using anti-phospho-(S/T) Akt substrates. HM motif phosphorylation of PKB and PKBR1 was detected using anti phospho p70 S6 kinase Thr389. Anti alpha tubulin was used to detect tubulin as a loading control. To test the developmental marker protein CsA, anti-CsA (123-353-1) primary antibody was used.

Expression of 6xHis Tagged Erk Proteins

For bacterial overexpression the coding sequences of ErkA and ErkB were amplified from cDNA using the primer pairs oPI390/oPI391 (ErkA) and oPI341/oPI343 (ErkB). For the cloning of the resulting PCR products into the pRSET-6xHis expression, plasmid DNA was cut with *Fse*I and *Acs*I respectively. Point mutagenesis for ErkB was performed using the primer pairs oPI377/oPI378 (T176A), oPI373/oPI374 (Y178A) and oPI367/oPI368 (T176A Y178A) using pPI486 (pRSET-6xHis-ErkB) as template. See Table S6 for details of oligonucleotide sequences.

E. coli cells (BL21-CodonPlus (DE3)-RIL) were transformed with the desired bacterial expression plasmid: pPI486 (pRSET-6xHis-ErkB), pPI528 (pRSET-6xHis-ErkB T176A Y178A), pPI544 (pRSET-6xHis-ErkB T176A), pPI560 (pRSET-6xHis-ErkB Y178A) or pPI580 (pRSET-6xHis-ErkA).

For overexpression cells were grown to an optical density (OD_{600}) of 0.6 at 37°C and chilled down to 16°C before expression was induced with 0.5 mM IPTG. Cells were grown over night at 16°C for protein overexpression. Cultures were harvested at 5000 x g. The collected cells pellets were frozen down in liquid N_2 in presence of 4 mg chicken egg lysozyme and stored at -20°C overnight.

Cell pellets were resuspended in IMAC buffer (50 mM HEPES, pH 8.0, 150 mM NaCl, 10% Glycerol, 1 mM Imidazole, 1 mM DTT) containing phosphatase inhibitors (PhosSTOP, Roche) and protease inhibitors (Roche complete EDTA free). The final cell lysis was done by sonication (amplitude 40%, 10 pulses 5 times with 1 min break between every 10 pulses). The resulting cell lysate was spun down at 22 000 x g for 30 min and 4°C. The collected supernatant was bound to a NiNTA column. The column was washed three times with IMAC buffer with increasing concentration of imidazole supplemented with 5 mM ATP and 5 mM $MgCl_2$. For protein elution IMAC buffer with 150 mM imidazole was used.

The collected elutions were dialysed overnight in a 50 mM Tris pH 8.0, 10% glycerol containing buffer. The purified protein was concentrated using Vivaspin 20 columns, aliquoted and stored at -80°C.

In Vitro Kinase Assay

Phosphorylation of substrate proteins or peptides was performed for 30 min at 30°C in kinase buffer (20 mM HEPES, 5 mM, 0.03 % TritonX, 100 μM ATP, 0.002 μCi/ml ^{33}P- γ ATP) using 100 ng purified 6xHis-ErkB (or point mutants) and 10 μg protein or peptide substrate in a 30 μl reaction volume. Protease and phosphatase inhibitors were added before starting the reaction. For the auto-phosphorylation assay 10 μg of kinase was used.

20 μl samples were spotted on P81-Whatman-paper (GE Healthcare 3698+915) squares (2 cm x 2 cm) and washed three times with 1% phosphoric acid for 10 min, followed by a final 5 min wash in acetone. The papers were dried and transferred into scintillation vials containing 20 ml LSC cocktail (ULTIMA GOLD) and ^{33}P incorporation was measured in a scintillation analyser (PerkinElmer).

Where substrate proteins were analysed, the remaining 10 μl of each reaction was mixed with 4x SDS-sample buffer and separated on a gel. The dried gel was exposed to an imaging plate and developed using a phospho-imager (Typhoon FLA 700).

For mass spectrometry analysis 10 μg 6xHis-ErkB was used and the kinase buffer used was as above, but without TritonX and 0.002 μCi/ml ^{33}P-γATP.

Generation of *Dictyostelium* Knockout Mutants

Knockout mutants of *ErkA* (dictyBase: DDB_G0286353), *ErkB* (dictyBase: DDB_G0283903) and *DhkI* (DDB_G0273475) were made in the Ax2 wild-type background by homologous recombination. Regions flanking *ErkB* were amplified by PCR using primer pairs SK420/SK421 and SK416/SK417. Regions flanking *ErkA* were amplified using primer pairs SK410/SK411 and SK412/SK413, while regions flanking *DhkI* were amplified using the primer pairs SK328/SK329 and SK330/331. These fragments were recombined into the plasmid pKOSG-IBA-Dicty1 using the Stargate cloning system (IBA GmbH) and the resulting plasmids cloned into *E. coli* and validated by Sanger sequencing. Plasmid DNA was digested with *Eco*RI (*ErkB*) or PstI (*ErkA, DhkI*) and transformed into Ax2 amoebae by electroporation. Briefly: amoebae were harvested then washed and resuspended in cold H50 buffer (20mM HEPES, 50mM KCl, 10mM NaCl, 1mM Mg_2SO_4, 5mM $NaHCO_3$, 1mM Na_2HPO_4). 4x10^6 cells in a volume of 100μl were mixed with 15μg digested plasmid DNA in a 1mm gap cuvette, electroporated (750 V, 25 μF, 2 pulses 5 s apart), and recovered into HL5. Ax2 transformants were selected by growth in HL5 containing 10 μg/ml Blasticidin in 24-well plates. Resistant cells were screened for by PCR using primers flanking the homologous recombination site (*erkB*$^-$: SK422, SK425. *erkA*$^-$: SK426, SK429, *dhkI*$^-$: SK349 and SK352). Wells yielding PCR products indicating knockout cells were harvested and plated with *K. aerogenes* on SM agar to isolate clonal populations of cells that were subsequently re-screened by PCR to confirm gene disruption. For the ErkA/ErkB double knockout a second construct for the disruption of *ErkB* was generated using a hygromycin resistance cassette. The flanking regions were amplified using the primer pairs oPI172/oPI173 and oPI174/oPI175. The first fragment was cloned via its *Ngo*MIV sites into pDM1081. The second fragment was integrated into the vectors *Bgl*II/*Spe*I sites. The fidelity of the recombination arms was confirmed with Sanger sequencing. The plasmid was linearised with *Spe*I/*Fse*I and transformed into *erkA*$^-$ cells. 60 μg/ml Hygromycin in HL5 was used for used for selection of clones. Resistant cells were screened using the primer pairs SK426/SK429 and oPI196/oPI265. See Table S6 for details of oligonucleotide sequences.

Growth on Bacterial Lawn

Dictyostelium cells grown on bacterial plates or in axenic shaking suspension were washed once and resuspended in 1 ml KK_2 buffer. Cell concentration was determined and serial dilutions performed to bring cells to a final concentration of 20 cells/ml in a stationary *K. aerogenes* suspension. To guarantee single colony growth 1 ml of this suspension was spread on three SM-agar plates. Colony diameters were measured after 4,5 and 6 days.

Uptake Assays

Macropinocytosis and bead uptake assays were performed using flow cytometry. To ensure the use of healthy cells for the uptake assays and to avoid any influence on uptake caused by the bacterial growth defect of *erkB*$^-$ mutant cells, only cells grown in plates supplemented with HL5 were used. The day before the assay cells were seeded at a density of 2x10^5 cells/ml in 96-well plates, using a total volume of 50 μl. Uptake was measured the next day after 60 min of feeding. For macropinocytosis assay cells were fed with HL5 medium supplemented with TRITC-dextran at final concentration of 0.5 mg/ml. Bead uptake was performed with 1.5 μm YG beads (Poly Science) used at a final concentration of 10^8 beads/ml. Following incubation cells were washed twice in KK_2 and

resuspended in 100 μl KK_2 containing 5 mM sodium azide to detach cells and prevent exocytosis, Cells were analysed with a flow cytometer (LSR-II, BD Bioscience) using the high-throughput sampling attachment. Data analysis was performed with FlowJo software. For the yeast uptake assay, cells were fed for 60 min with TRITC-labelled yeast particles. After quenching with trypan blue, yeast particle uptake was quantified by counting particles internalized by 100 amoebae using a fluorescence microscope equipped with a 20x air objective.

Cell Size

The size of cells was determined using an Eclipse flow cytometer (Sony iCyt). Cells were taken from bacterial suspension or plates supplemented with HL5 medium and washed 2x before being resuspended in KK_2-MC. For measurement, samples were passed through a 20 μm filter unit to remove cell aggregates. Low-density samples of a concentration of about $1x10^5$ cells/ml were measured to avoid the quantification of doublets.

Developmental Time Course

All developmental time courses were performed on gridded filters. Cells were taken from clearing plates, harvested and washed 5x times in KK_2-MC buffer to remove bacteria and induce starvation. Cells were resuspended to a final concentration of $2.5x10^7$ cells/ml. Filters were soaked in KK_2-MC and placed on equally wet Whatman paper to ensure sufficient humidity. Cells were pipetted on the filters and allowed to settle for 15 min before residual buffer was removed. Images were taken at 4 h intervals using a low magnification microscope equipped with a camera.

Micropipette Chemotaxis Assay

Undifferentiated Ax2 and *erkB*$^-$ amoebae were harvested from *K. aerogenes* clearing plates and washed six times in KK_2-MC to remove bacteria. Cells were resuspended at $2x10^7$ cells/ml and shaken in suspension for 30 minutes before being washed again then diluted in KK_2-MC and placed in a modified 2-well chambered coverglass (Nunc Lab-tek) with cut-down sides. Cells were left to settle for 30 minutes before filming using a Zeiss Axiovert S100 inverted microscope with 20x phase contrast objective. A micropipette (Eppendorf Femtotip II) filled with 100 μM folate was placed in the centre of the field of view just above the plane of the coverglass using a micromanipulator (Eppendorf Injectman and Femtojet). Cell movement was imaged at 2 frames per minute for a 40 minutes period. Cells were tracked using the manual tracking plugin of FIJI ImageJ (https://fiji.sc/) and chemotaxis parameters extracted using the Ibidi chemotaxis tool software (Ibidi).

Under Agarose Folate Chemotaxis Assay

With the exception of *gacG*$^-$ cells, all cells used for the under agarose chemotaxis assay were grown in HL5. *gacG*$^-$ cells were taken from *K. aerogenes* clearing plates and compared to Ax2 cells grown under the same conditions. Cells were washed 5 times and resuspended to a final concentration of $2x10^6$ cells/ml in LoFlo medium. In parallel, 0.5% low melting point agarose (SeaKem) dissolved in LoFlo medium containing 10 μM (1 μM or 0.1 μM) was pipetted into 50 mm glass bottom dishes (MatTek). A well was cut into the agarose layer and $2x10^5$ cells were transferred into it. Cells were allowed to crawl under the agarose before starting imaging. Microscopy was performed on a Zeiss 710 confocal microscope equipped with a 10x air objective. Cells were filmed for 90-120 min with a frame rate of 2 frames per minute. For data analysis cells that could be filmed for at least 60 min were selected. Chemotaxis parameters were calculated as described for the micropipette assay.

Insall Chamber Chemotaxis Assay

Cells used for cAMP chemotaxis assays were grown on *K. aerogenes* clearing plates. To remove remaining bacteria, cells were washed 5x times with KK_2-MC. Cells were prepared as described for the cAMP stimulation in SILAC experiments, with cAMP pulsing for 4 hours duration. Cells were washed once in KK_2-MC to remove extracellular cAMP, resuspended in KK_2-MC and diluted down to a final concentration of $4x10^5$ cells/ml. $4x10^4$ cells were transferred into an Insall chamber with the concentration of the cAMP source at 1 μM. Microscopy was performed on a Zeiss 710 confocal microscope equipped with a 10x air objective. Cells were filmed for 1 h using a 20 sec frame rate. Data recording was started directly after assembling the chemotaxis chamber. Randomly selected cells that were tracked for at least 45 frames were used for data analysis. Chemotaxis parameters were calculated as described for the micropipette assay.

F-Actin Polymerization Assays

Cells were prepared for folate stimulation as described for immunoblot assays. F-actin content was determined after folate stimulation by extracting triton-insoluble cytoskeletons and staining by incubation with TRITC-labeled phalloidin (Sigma). Briefly; cells were fixed and stained in 5 volumes of Fix/Stain buffer (30 mM PIPES, 6 mM EGTA, 2.4 mM $MgCl_2$, 3% (v/v) formaldehyde, 0.24% (v/v) Triton X-100, 0.6 μM TRITC-phalloidin) for 10 minutes with end-over-end mixing. Triton-insoluble cytoskeletons were pelleted by centrifugation for 2 minutes at 13000 x g, and washed in 1.5 ml of Wash buffer (25 mM PIPES, 5 mM EGTA, 2 mM $MgCl_2$, 0.1% Saponin) for 30 minutes with end-over-end mixing. After pelleting by centrifugation again, incorporated TRITC-phalloidin was extracted in 1 ml of methanol. Fluorescence was measured using a Perkin Elmer LS50B fluorimeter (excitation 544 nm, emission 572 nm, 4 mm slit width).

F-actin/total actin measurements were made using undifferentiated amoebae harvested from clearing plates and washed five times in KK_2-MC. Cells were resuspended at $2x10^7$ cells/ml in KK_2-MC and shaken for 20 minutes, before being collected by centrifugation and resuspended at $1x10^8$ cells/ml. Cell samples were lysed by addition of an equal volume of 2x cytoskeleton buffer (2% Triton X-100, 20 mM KCl, 20 mM EDTA, 4 mM NaOAc) and incubation on ice for 10 minutes, followed by rotation at room temperature for 10 minutes. Cell cytoskeleton fractions were collected by centrifugation at 8000 x g for 4 minutes, washed in 1x cytoskeleton buffer, and centrifuged again at 8000 x g for 4 minutes before being resuspended in 1x LDS sample buffer with 2-mercaptoethanol, protease inhibitors and phosphatase inhibitors. In parallel, whole cell extracts were prepared by direct lysis of cell samples in 3x LDS sample buffer with 2-mercaptoethanol, protease inhibitors and phosphatase inhibitors. Dilutions of cytoskeleton and whole-cell samples were separated by SDS-PAGE and immunoblotted using anti-actin primary antibody (as above), Band intensity of diluted whole cell extracts were used to plot a reference curve of total actin against which F-actin content of cytoskeleton samples was assessed. Measurements of actin immunoblot band intensity were made using FIJI ImageJ.

Live-Cell Imaging of Actin Cytoskeleton

Actin structures were visualised by expressing a LifeAct:mCherry fusion in wild-type and *erkB*$^-$ mutant amoebae. To do this, cells were transformed with a dual expression plasmid (pPI304) containing LifeAct fused to mCherry and the PH domain of PkgE fused to GFP, both under the control of *Dictyostelium act15* promoters; as well as Geneticin resistance under the control of the *coaA* promoter. To construct pPI304, the PH-PkgE:GFP from pDM1398 was subcloned into the expression backbone plasmid pDM1209 to create pPI291. LifeAct:mCherry was cloned into the shuttle vector pDM344, before being transferred into pPI291 to create pPI304. pDM1398 and pDM1209 were a kind gift from Dr. Douwe Veltman.

Ax2 and *erkB*$^-$ amoebae for transformation were harvested from *K. aerogenes* clearing plates, washed and resuspended at $4x10^7$ cells/ml in cold H40 buffer (40 mM HEPES pH 7). 100 μl of cells were mixed with 1 μg of pPI304 plasmid and transferred to a pre-chilled 2mm gap electroporation cuvette. Electroporation was conducted at 350V, with two pulses of 8ms applied at a 1s interval. Cells were transferred to a petri dish containing SorMC buffer (2 mM Na_2HPO_4, 15 mM KH_2PO_4, 50 μM $CaCl_2$, 50 μM $MgCl_2$) with *K. aerogenes* (OD_{600} = 2). After 5 hours, selection was added (Geneticin 20 μg/ml). After 2 days selection, cells were harvested and washed free from bacteria then allowed to settle on a chambered coverglass (Nunc Lab-tek) for 30 minutes. Lifeact:mCherry was imaged using a Zeiss 780 confocal microscope. Dynamic F-actin structures were imaged over a 15-minute period at 2 frames per minute with confocal slices taken at 370nm intervals. Localization of F-actin puncta was imaged with confocal slices taken at 250nm intervals. Maximum intensity projections were generated using FIJI ImageJ.

Imaging of F-actin in *gacG*$^-$ cells was performed as above.

Phylogenetic Analysis of MAPK Sequences

Sequences were downloaded from Kinbase (http://kinase.com/kinbase/) and dictyBase (http://dictybase.org/). Sequences were aligned using MUSCLE (v3.8.31). Ambiguous regions were removed using Gblocks (v0.91b), resulting in an alignment focused on the kinase domain of the proteins. Tree construction used the maximum likelihood method in PhyML (v3.1/3.0 aLRT) and was rendered using TreeDyn(v198.3). The analysis was performed on the Phylogeny.fr platform.

QUANTIFICATION AND STATISTICAL ANALYSIS

Gene Ontology Enrichment Analysis

Gene ontology enrichment analysis was conducted using the Orange Data Mining Toolbox (https://orange.biolab.si/). Foreground sets of chemoattractant-regulated phosphorylation sites were compared against a background of all phosphorylation sites identified in experiments with the same chemoattractant. Only terms with 3 or more input genes assigned to them were tested. Significance of enrichment was tested with the binomial significance test setting.

Mutant Phenotype *In Silico* Analysis

Mutant phenotype information was downloaded from DictyBase (Basu et al., 2015) and cross-referenced against our phosphoproteome data. Over-representation of chemotaxis phenotypes was tested for using Fisher's exact test.

Motif Discovery and Enrichment Analysis

De novo motif discovery used pLogo (https://plogo.uconn.edu/). Chemoattractant responsive sites in the cAMP/folate intersection were used as the foreground set. All phosphorylation sites detected in cAMP and folate experiments were used as the background set. N for foreground and background sets are shown in Figure 2. Known kinase substrate consensus motifs taken from the literature (Gnad et al., 2011; Ubersax and Ferrell, 2007) were used in custom Perl script to extract matching phosphorylation sites using the MaxQuant sequence window. Where more complex motif sequences exist entirely as a subset of more simple motifs, sites matching the complex motif were removed from the set of sites matching the simple motif. In practice, this resulted in PKA, PKB and Pak sites being subtracted from the CAMK motif set; and p[S/T]PR, MAPK and CDK sites being subtracted from the proline-directed motif set. Statistical enrichment was conducted using the Fisher.test function in the R programming language with the *alternative* parameter set to 'greater'. P values were adjusted for multiple-testing by the Benjamini-Hochberg method. N values of sites matching different motifs are shown in Figure 2.

Chemotaxis Assays

Details for analysing chemotaxis data: In the micropipette assay, chemotactic index was defined as the cosine of the angle between the cell's vector of movement between frame 10 and frame 30 and a line between the cell's position in frame 10 and the micropipette. In under agarose and Insall chamber assays, chemotactic index was defined as the cosine of the angle between the cell's vector of movement over the duration of tracking and the direction of the chemoattractant gradient. Persistence was defined as Euclidean distance divided by accumulated distance. Speed defined as accumulated distance divided by time. In the micropipette assay, parameters for individual cells were averaged for each day's experiment (16-20 cells per day), with n = 4 day's experiments used for statistical test of difference between wild-type and mutant grand means. Statistical testing of chemotaxis parameters in the micropipette assay was conducted using unpaired two-tailed Student's t test, which assumes samples are drawn from a Gaussian distribution with both wild-type and mutant populations having equal standard deviations. Statistical testing was performed using Graphpad Prism. Under agarose and cAMP chemotaxis assays were analyzed in the same way, with statistical testing of chemotaxis parameters using Welch's t test. Details of graphical presentation and results of statistical testing for chemotaxis experiments are shown in Figures 6, S5, and S7, and their respective figure legends

F-Actin Polymerization Quantification

For time-course assay; n = 3 independent experiments conducted on different days. Mean values are plotted with error bars showing standard deviation. For quantification of resting F-actin; n = 3 independent experiments conducted on different days. Values are plotted with horizontal bar showing mean and error bars showing standard deviation. Statistical testing of difference between wild-type and mutant mean value was conducted by unpaired Student's t test, which assumes samples are drawn from a Gaussian distribution and that wild-type and mutant populations have equal standard deviation. P = 0.0015. Statistical testing was performed using Graphpad Prism.

DATA AND SOFTWARE AVAILABILITY

Phosphorylation site data including SILAC ratios is included as Tables S3 and S5. Complete intersection of cAMP and folate phosphorylation data is included in Table S4. Full results of Gene Ontology enrichment analysis are provided in Table S4.

Developmental Cell, Volume *48*

Supplemental Information

The Atypical MAP Kinase ErkB Transmits Distinct Chemotactic Signals through a Core Signaling Module

John M.E. Nichols, Peggy Paschke, Sew Peak-Chew, Thomas D. Williams, Luke Tweedy, Mark Skehel, Elaine Stephens, Jonathan R. Chubb, and Robert R. Kay

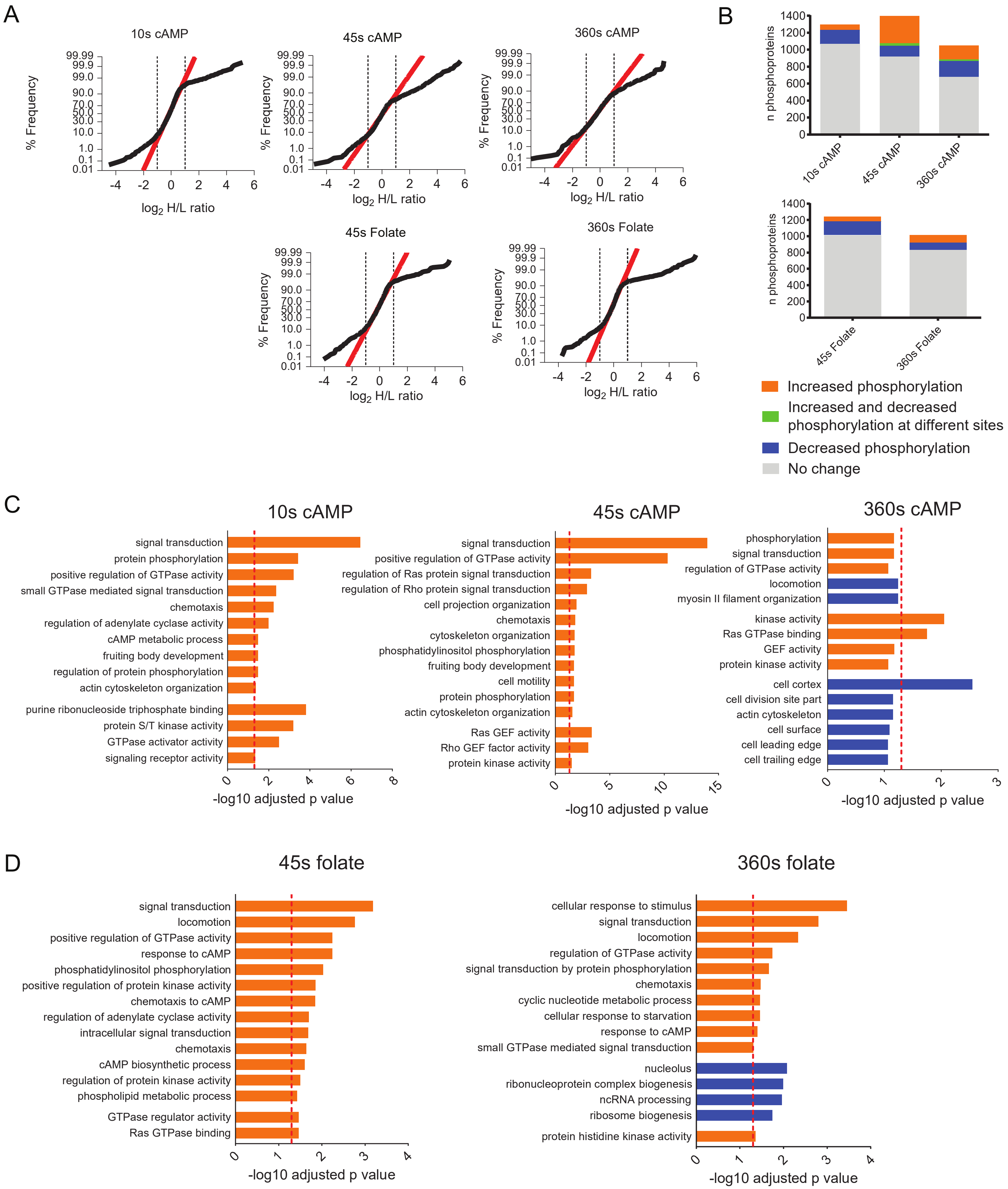
A
10s cAMP
45s cAMP
360s cAMP
45s Folate
360s Folate
% Frequency
99.99
99.9
99.0
90.0
70.0
50.0
30.0
10.0
1.0
0.1
0.01
-4 -2 0 2 4 6
log2 H/L ratio
B
n phosphoproteins
1400 1200 1000 800 600 400 200 0
10s cAMP
45s cAMP
360s cAMP
45s Folate
360s Folate
Increased phosphorylation
Increased and decreased phosphorylation at different sites
Decreased phosphorylation
No change
C
10s cAMP
signal transduction
protein phosphorylation
positive regulation of GTPase activity
small GTPase mediated signal transduction
chemotaxis
regulation of adenylate cyclase activity
cAMP metabolic process
fruiting body development
regulation of protein phosphorylation
actin cytoskeleton organization
purine ribonucleoside triphosphate binding
protein S/T kinase activity
GTPase activator activity
signaling receptor activity
0 2 4 6 8
-log10 adjusted p value
45s cAMP
signal transduction
positive regulation of GTPase activity
regulation of Ras protein signal transduction
regulation of Rho protein signal transduction
cell projection organization
chemotaxis
cytoskeleton organization
phosphatidylinositol phosphorylation
fruiting body development
cell motility
protein phosphorylation
actin cytoskeleton organization
Ras GEF activity
Rho GEF factor activity
protein kinase activity
0 5 10 15
-log10 adjusted p value
360s cAMP
phosphorylation
signal transduction
regulation of GTPase activity
locomotion
myosin II filament organization
kinase activity
Ras GTPase binding
GEF activity
protein kinase activity
cell cortex
cell division site part
actin cytoskeleton
cell surface
cell leading edge
cell trailing edge
0 1 2 3
-log10 adjusted p value
D
45s folate
signal transduction
locomotion
positive regulation of GTPase activity
response to cAMP
phosphatidylinositol phosphorylation
positive regulation of protein kinase activity
chemotaxis to cAMP
regulation of adenylate cyclase activity
intracellular signal transduction
chemotaxis
cAMP biosynthetic process
regulation of protein kinase activity
phospholipid metabolic process
GTPase regulator activity
Ras GTPase binding
0 1 2 3 4
-log10 adjusted p value
360s folate
cellular response to stimulus
signal transduction
locomotion
regulation of GTPase activity
signal transduction by protein phosphorylation
chemotaxis
cyclic nucleotide metabolic process
cellular response to starvation
response to cAMP
small GTPase mediated signal transduction
nucleolus
ribonucleoprotein complex biogenesis
ncRNA processing
ribosome biogenesis
protein histidine kinase activity
0 1 2 3 4
-log10 adjusted p value

Figure S1. Phosphorylation changes in response to cAMP and folate treatment. Related to Figure 1.

(A) Cumulative frequency plots showing distributions of SILAC ratios for cAMP and folate phosphoproteomic experiments. Red line shows Gaussian distributions fitted to the data for each experiment. Dashed lines show 2-fold SILAC ratio threshold used to define phosphorylation changes.
(B) Numbers of phosphoproteins with phosphorylation sites detected in each cAMP and folate experiment.
(C) and (D) GO analysis of phosphorylation changes following cAMP treatments (C) and folate treatments (D). Orange bars denote enrichment among sites of increased phosphorylation. Blue bars denote enrichment among sites with decreased phosphorylation. Dashed line indicates p=0.05. Reported p-values corrected for multiple comparisons using the Benjamini-Hochberg method.

A

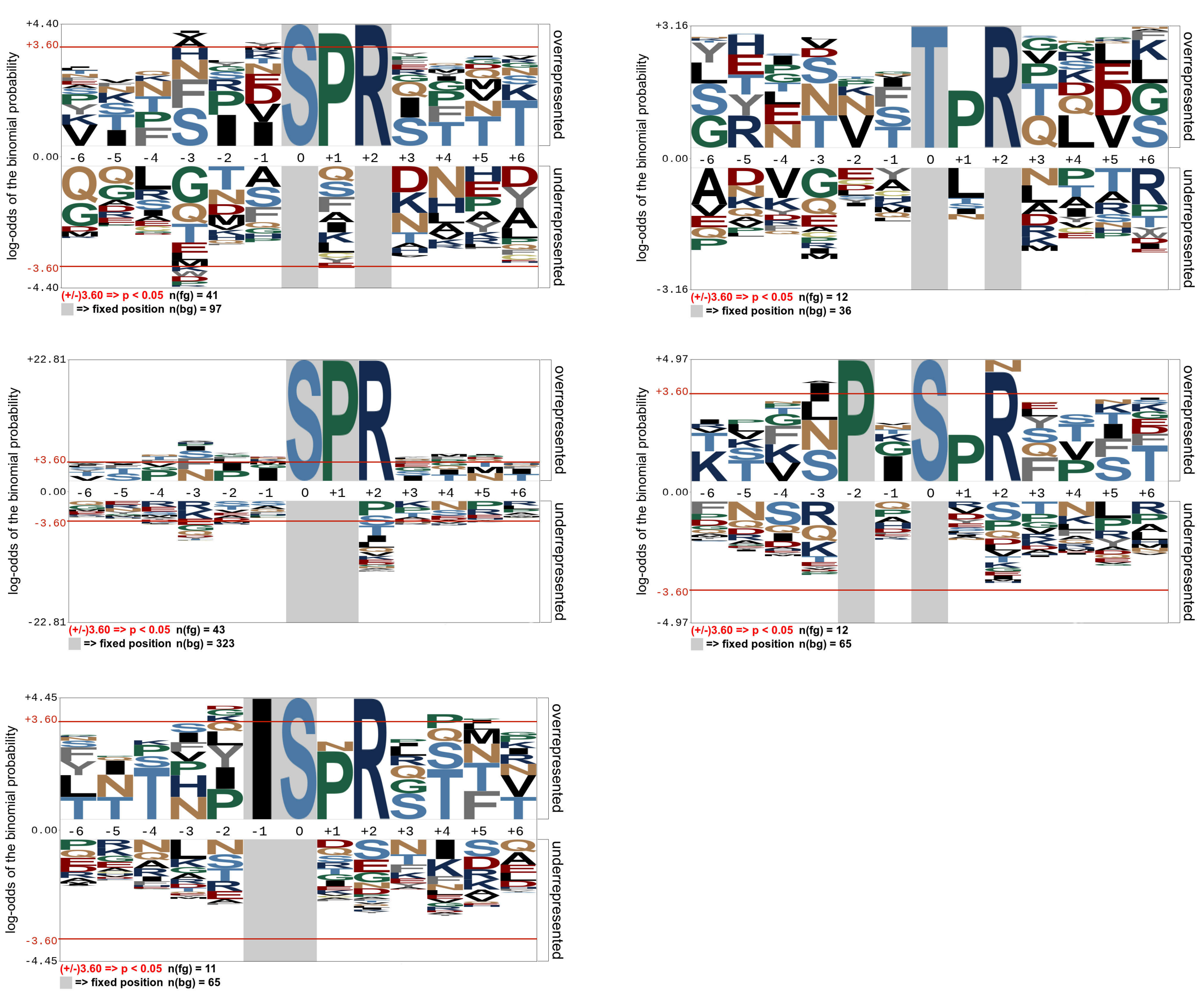

B

Increased phosphorylation

motif	10s cAMP	45s cAMP	360s cAMP	45s folate	360s folate
[S/T]PR	1	$6.8x10^{-9}$	$5.3x10^{-27}$	$1.2x10^{-36}$	$3.03x10^{-77}$
PKB	$2.7x10^{-8}$	$6.0x10^{-10}$	$4.8x10^{-3}$	0.06	1
PKA	0.01	$3.1x10^{-8}$	$4.8x10^{-9}$	1	0.04
CAMK	$5.1x10^{-4}$	$1.1x10^{-12}$	1	1	1
MAPK	1	1	0.07	$2.3x10^{-6}$	$1.25x10^{-5}$
CK1	1	0.01	0.29	0.86	0.43

Decreased phosphorylation

motif	10s cAMP	45s cAMP	360s cAMP	45s folate	360s folate
CK2	$4.2x10^{-32}$	$2.0x10^{-56}$	$3.8x10^{-27}$	$1.3x10^{-51}$	$1.3x10^{-63}$

C

Name	Sequence
[S/T]PR	p[ST]PR
Proline directed	p[ST]P
MAPK	Pxp[ST]
CDK	p[ST]Px[KR]
GSK	p[ST]xxx[ST]
CK2	p[ST][DE]x[DE]
PKA	RRxp[ST]Φ
PKB	RxRxxp[ST]
Pak	[RK]R[RK]p[ST]
CAMK	Rxxp[ST]
Nek	[FL]xxp[ST]
CK1	p[ST]xx[ST]

Figure S2. Identification of novel and known kinase consensus motifs in phosphoproteomic data. Related to Figure 2.

(A) Extended sequence logos, generated using pLogo, showing coincidence of residues making up the p[S/T]PR motif. From the base logos shown in Figure 2, a statistically overrepresented residue can be 'fixed' and used as the basis for set reduction to investigate the conditional probabilities of correlations between residues; allowing extended logos to be built. For both phosphoserine and phosphothreonine sites, when statistically significant residues from the base logo are fixed (grey box), it is observed that proline at +1 and arginine at +2 are both consistently overrepresented in the extended logos, suggesting close association of these residues in cAMP and folate regulated phosphorylation sites.
(B) Results of statistical overrepresentation of different kinase consensus motifs amongst phosphorylation sites with chemoattractant responses. Overrepresentation was tested using Fisher's exact test and p-values are adjusted using the Benjamini-Hochberg method.
(C) Kinase substrate motif consensus sequences used for classification of phosphorylation sites in Figure 2B.

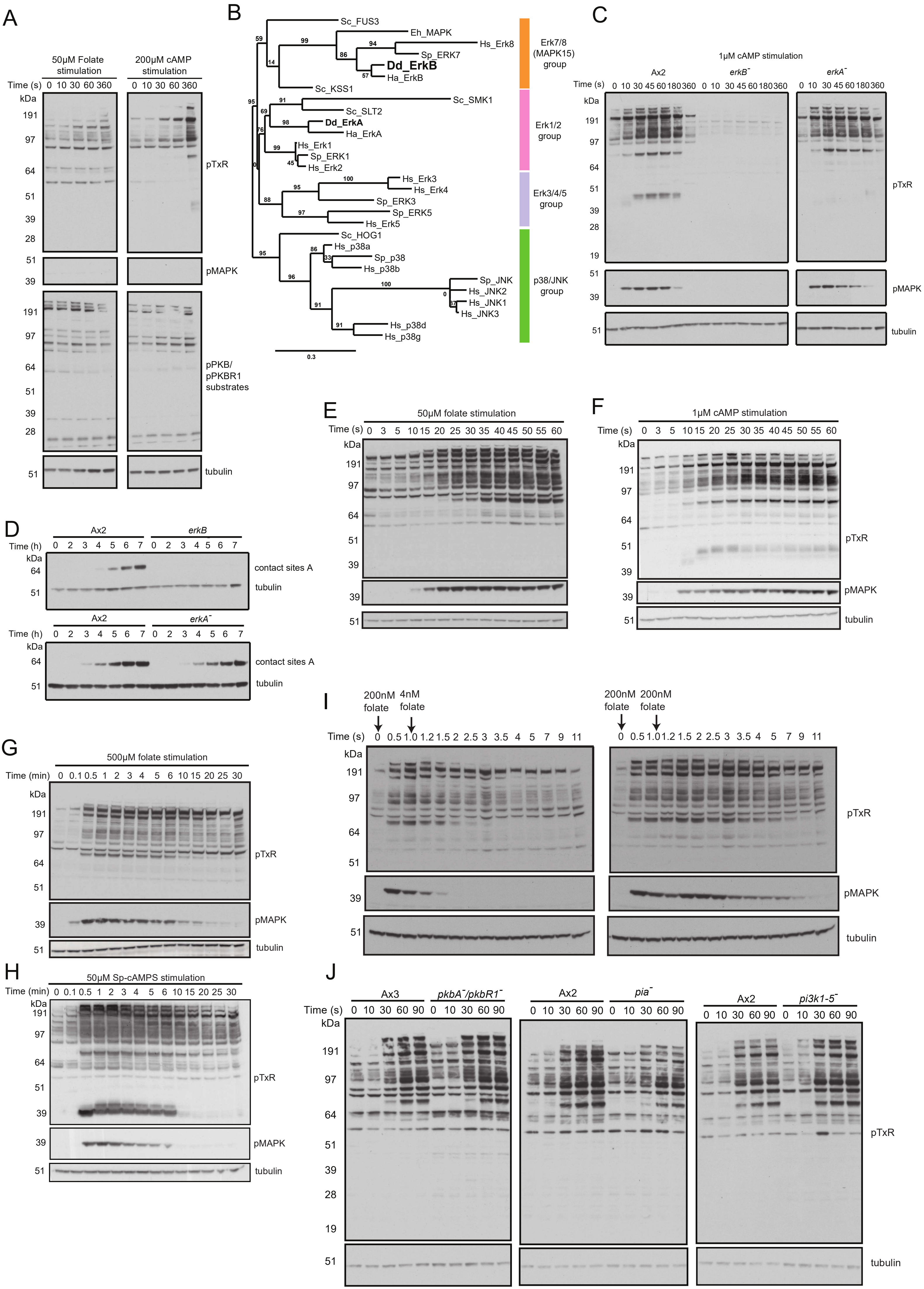

A
50µM Folate stimulation
200µM cAMP stimulation
Time (s) 0 10 30 60 360
kDa
pTxR
pMAPK
pPKB/ pPKBR1 substrates
tubulin
B
Sc_FUS3
Eh_MAPK
Hs_Erk8
Sp_ERK7
Dd_ErkB
Ha_ErkB
Sc_KSS1
Sc_SMK1
Sc_SLT2
Dd_ErkA
Ha_ErkA
Hs_Erk1
Sp_ERK1
Hs_Erk2
Hs_Erk3
Hs_Erk4
Sp_ERK3
Sp_ERK5
Hs_Erk5
Sc_HOG1
Hs_p38a
Sp_p38
Hs_p38b
Sp_JNK
Hs_JNK2
Hs_JNK1
Hs_JNK3
Hs_p38d
Hs_p38g
0.3
Erk7/8 (MAPK15) group
Erk1/2 group
Erk3/4/5 group
p38/JNK group
C
1µM cAMP stimulation
Ax2
erkB⁻
erkA⁻
Time (s) 0 10 30 45 60 180 360
pTxR
pMAPK
tubulin
D
Ax2
erkB
erkA⁻
Time (h) 0 2 3 4 5 6 7
contact sites A
tubulin
E
50µM folate stimulation
Time (s) 0 3 5 10 15 20 25 30 35 40 45 50 55 60
F
1µM cAMP stimulation
Time (s) 0 3 5 10 15 20 25 30 35 40 45 50 55 60
G
500µM folate stimulation
Time (min) 0 0.1 0.5 1 2 3 4 5 6 10 15 20 25 30
H
50µM Sp-cAMPS stimulation
Time (min) 0 0.1 0.5 1 2 3 4 5 6 10 15 20 25 30
I
200nM folate
4nM folate
200nM folate
200nM folate
Time (s) 0 0.5 1.0 1.2 1.5 2 2.5 3 3.5 4 5 7 9 11
J
Ax3
pkbA⁻/pkbR1⁻
Ax2
pia⁻
pi3k1-5⁻
Time (s) 0 10 30 60 90

Figure S3. Investigating pTxR motif and pMAPK phosphorylation by immunoblotting. Related to Figure 3.

(A) Buffer control immunoblots for folate and cAMP treatments shown in Figure 3A. Phosphorylation in 360 s cAMP control sample likely to be caused by endogenous cAMP release from aggregation-competent amoebae.

(B) Maximum likelihood tree of *D. discoideum* ErkA and ErkB and MAPKs from other selected species: *Homo sapiens* (Hs), sea urchin (*Strongylocentrotus purpuratus*, Sp), *Saccharomyces cerevisiae* (Sc), *Dictyostelium discoideum* (Dd), *Heterostelium album* (Ha), *Entamoeba histolytica* (Ea). *Dictyostelium* ErkB aligns with metazoan Erk7/ERk8/MAPK15, while *Dictyostelium* ErkA is grouped with metazoan Erk1/2. Sequences were aligned using MUSCLE (v3.8.31). Ambiguous regions were removed using Gblocks (v0.91b), resulting in an alignment focused on the kinase domain of the proteins. Tree construction used the maximum likelihood method in PhyML and was rendered using TreeDyn.

(C) Immunoblotting of pTxR phosphorylation and pMAPK following 1 µM cAMP treatment of cAMP-pulsed wild-type, *erkB*$^-$ and *erkA*$^-$ amoebae. Cells were pulsed at 6 minute intervals with 90 nM cAMP for 5 hours before being washed and stimulated with 1 µM cAMP. Phosphorylation responses are seen in wild-type and *erkA*$^-$ cells but not observed in *erkB*$^-$ cells. Tubulin shown as a loading control.

(D) Immunoblots showing expression of the developmental marker CsaA following cAMP pulsing treatment of different durations. In wild-type and *erkA*$^-$ cells, expression of CsaA is observed after 3 hours of pulsing with cAMP. In *erkB*$^-$ cells, CsaA expression is not observed even after 7 hours of cAMP pulsing treatment. Tubulin shown as a loading control.

(E) Immunoblot showing onset of pTxR motif phosphorylation and ErkB phosphorylation (pMAPK) at high temporal resolution following stimulation of undifferentiated amoebae with 50 µM folate. Tubulin shown as loading control. Quantification is shown in Figure 3E. Representative of 2 independent experiments.

(F) Immunoblot showing onset of pTxR motif phosphorylation and ErkB phosphorylation (pMAPK) at high temporal resolution following stimulation of cAMP-pulsed amoebae with 1 µM cAMP. Tubulin shown as loading control. Quantification is shown in Figure 3E. Representative of 2 independent experiments.

(G) Immunoblot showing duration of pTxR motif phosphorylation and ErkB phosphorylation (pMAPK) during continuous saturating stimulation of undifferentiated amoebae with 500 µM folate. Tubulin shown as loading control. Quantification is shown in Figure 3F. Representative of 2 independent experiments.

(H) Immunoblot showing duration of pTxR motif phosphorylation and ErkB phosphorylation (pMAPK) during continuous saturating stimulation of cAMP-pulsed *acaA*$^-$ amoebae with 50 µM Sp-cAMPS. Tubulin shown as loading control. Quantification is shown in Figure 3F. Representative of 2 independent experiments.

(I) Immunoblot showing dephosphorylation of pTxR motif phosphoproteins and of ErkB following stimulation of wild-type undifferentiated amoebae with 200 nM folate and subsequent dilution after 60 s in buffer to reduce folate concentration to 4 nM. Dilution of cells to the same extent with buffer containing 200 nM folate leads to continued phosphorylation. Tubulin shown as loading control. Blots shown are representative of two independent experiments. Quantification shown in Figure 3G.

(J) pTxR phosphorylation response (upper panels) to 50 µM folate treatment in chemotaxis signalling mutants and parent wild-type strains. The pTxR response is intact in knockout cells lacking PKB and PKBR1, cells lacking the TORC2 component Pianissimo, and cells lacking PI3K1-5. Similarly, the response is intact in knockouts of the PI5K PikI, RasC/G (singly and double), GcA/SgaA double, SCAR, PLA2, PKAcat, and IPLA (data not shown). Lower panels show tubulin as a loading control.

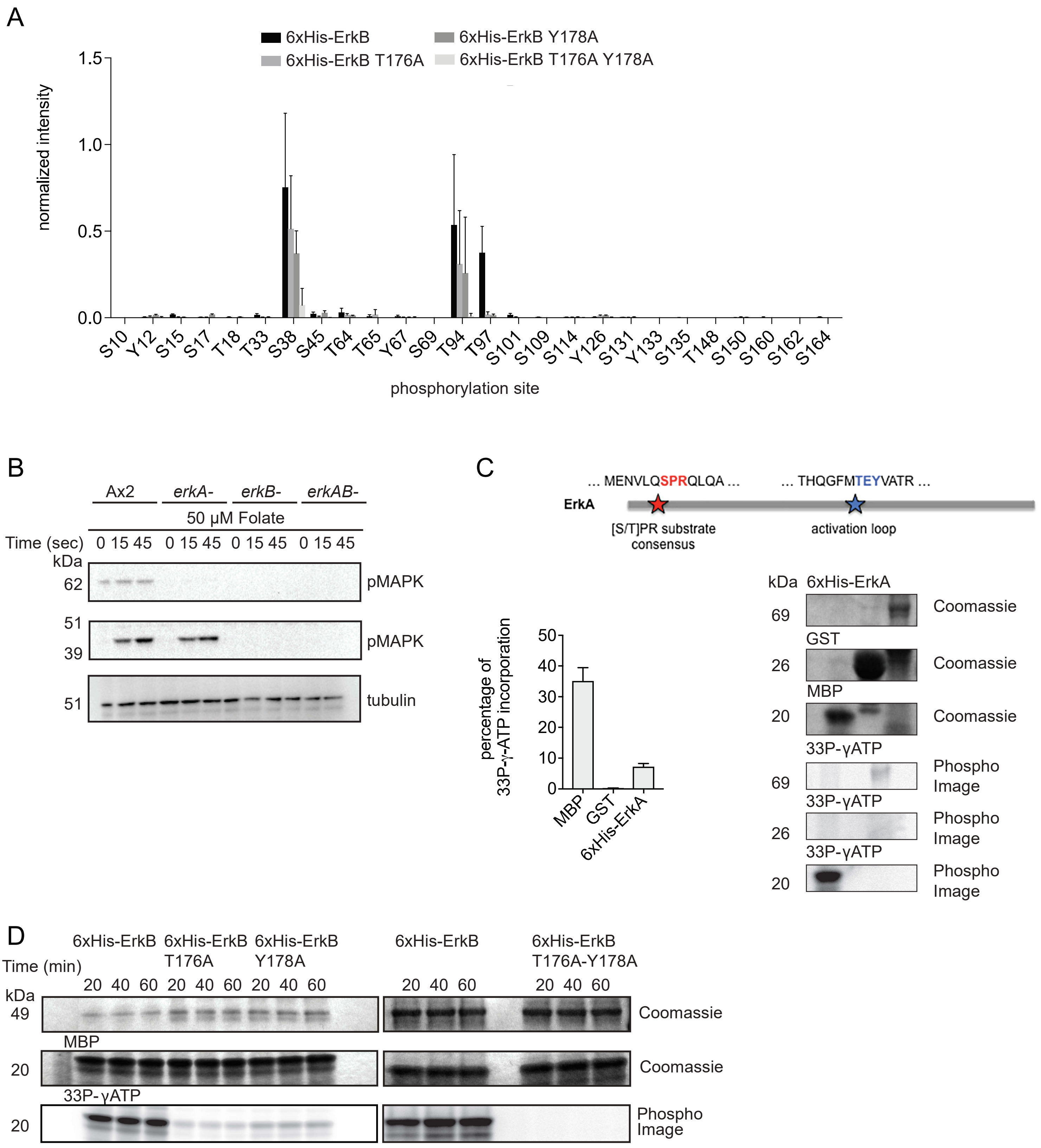

A
6xHis-ErkB
6xHis-ErkB Y178A
6xHis-ErkB T176A
6xHis-ErkB T176A Y178A
normalized intensity
1.5
1.0
0.5
0.0
S10 Y12 S15 S17 T18 T33 S38 S45 T64 T65 Y67 S69 T94 T97 S101 S109 S114 Y126 S131 Y133 S135 T148 S150 S160 S162 S164
phosphorylation site
B
Ax2
erkA-
erkB-
erkAB-
50 µM Folate
Time (sec) 0 15 45 0 15 45 0 15 45 0 15 45
kDa
62
pMAPK
51
39
pMAPK
51
tubulin
C
... MENVLQSPRQLQA ...
... THQGFMTEYVATR ...
ErkA
[S/T]PR substrate consensus
activation loop
percentage of 33P-γ-ATP incorporation
50
40
30
20
10
0
MBP
GST
6xHis-ErkA
kDa
6xHis-ErkA
69
Coomassie
GST
26
Coomassie
MBP
20
Coomassie
33P-γATP
69
Phospho Image
33P-γATP
26
Phospho Image
33P-γATP
20
Phospho Image
D
6xHis-ErkB
6xHis-ErkB T176A
6xHis-ErkB Y178A
6xHis-ErkB
6xHis-ErkB T176A-Y178A
Time (min)
20 40 60 20 40 60 20 40 60
20 40 60 20 40 60
kDa
49
Coomassie
MBP
20
Coomassie
33P-γATP
20
Phospho Image

Figure S4. Identification of ErkA as a ErkB substrate and analysis of ErkB activation loop and autophosphorylation. Related to Figure 5.

(A) Mass spectrometry analysis of phosphorylation sites on MBP substrate following the kinase assay with wild-type and activation loop point mutants of 6xHis-tagged ErkB. Phospho-site intensity is normalized such that the most abundant phospho-site signal in each experimental replicate = 1. Bar chart shows mean of N=3 experiments. Error bars indicate SD. No phosphorylation was detected in samples containing only MBP.

(B) Western blot of folate stimulated Ax2 wild-type and *erkA*⁻, *erkB*⁻ and *erkAB*⁻ mutant cells. Activation loop phosphorylation was analysed using a pMAPK antibody. ErkA activation loop phosphorylation could only be detected in wild-type cells and was abolished in *erkA*⁻, *erkB*⁻ and *erkAB*⁻ cells, indicating a dependency of ErkA phosphorylation on ErkB. Tubulin blot shown as loading control.

(C) Schematic overview of ErkA highlighting important structures present in the protein. The red star symbolises the position of the SPR motif close to the N-terminus of the protein. The blue star marks the position of the activation loop. Bacterial expressed 6xHis-ErkA was used for kinase assays. Both the paper-based assay and the gel assay show a direct phosphorylation of 6xHis-ErkA by 6xHis-ErkB. GST served as negative control while MBP was used as positive control. Coomassie gels are shown as loading controls. Graph shows the mean of three experiments. Error bars indicate SD.

(D) 6xHis tagged ErkB and activation loop mutants were incubated with Myelin Basic Protein (MBP) to assay their kinase activity by monitoring 33P-ATP incorporation. Different incubation times were used (20min, 30min and 40min) While the single mutants T176A and Y178A show a reduced activity independent of the incubation time, no substrate phosphorylation was detected in the T176A Y178A double mutant. Coomassie gels are shown to confirm equal sample loading. The phosphoimages illustrate phosphorylation of MBP.

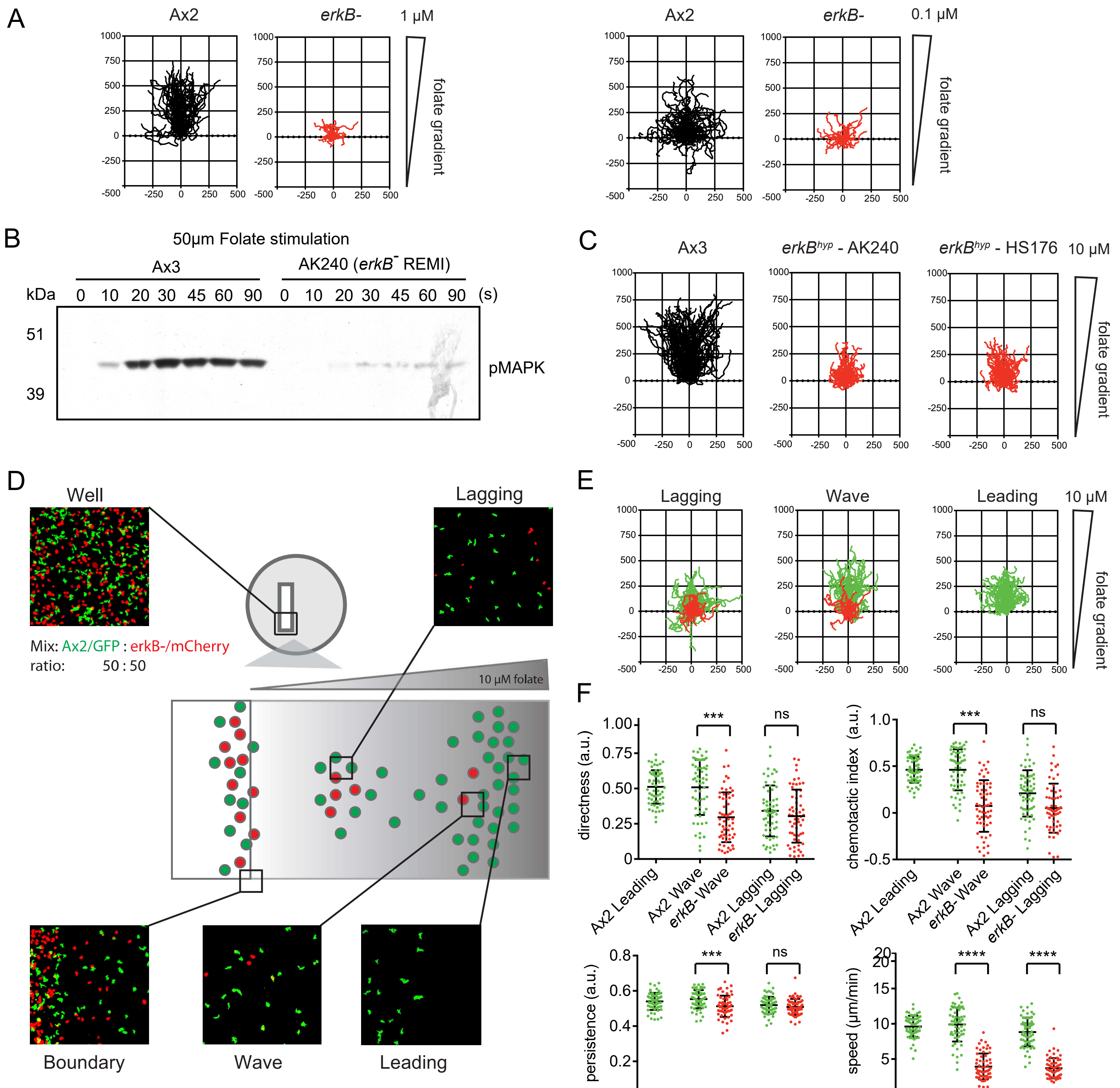

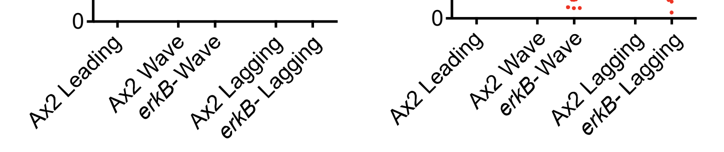

G

10 μM folate	Ax2 (wave)	*erkB-* (wave)	Ax2 (lagging)	*erkB-* (lagging)
**Chemotactic Index (a.u.)**	0.463<br>SD ± 0.219	0.074 ****<br>SD ± 0.275	0.210<br>SD ± 0.246	0.0506 ns<br>SD ± 0.260
**Speed (μm/min)**	9.902<br>SD ± 2.401	3.946 ****<br>SD ± 1.816	8.852<br>SD ± 3.240	3.879 ****<br>SD ± 1.567
**Persistence (a.u.)**	0.553<br>SD ± 0.051	0.512 ***<br>SD ± 0.060	0.518<br>SD ± 0.048	0.509 ns<br>SD ± 0.045
**Directness (a.u.)**	0.507<br>SD ± 0.194	0.300 ***<br>SD ± 0.174	0.341<br>SD ± 0.178	0.305 ns<br>SD ± 0.186

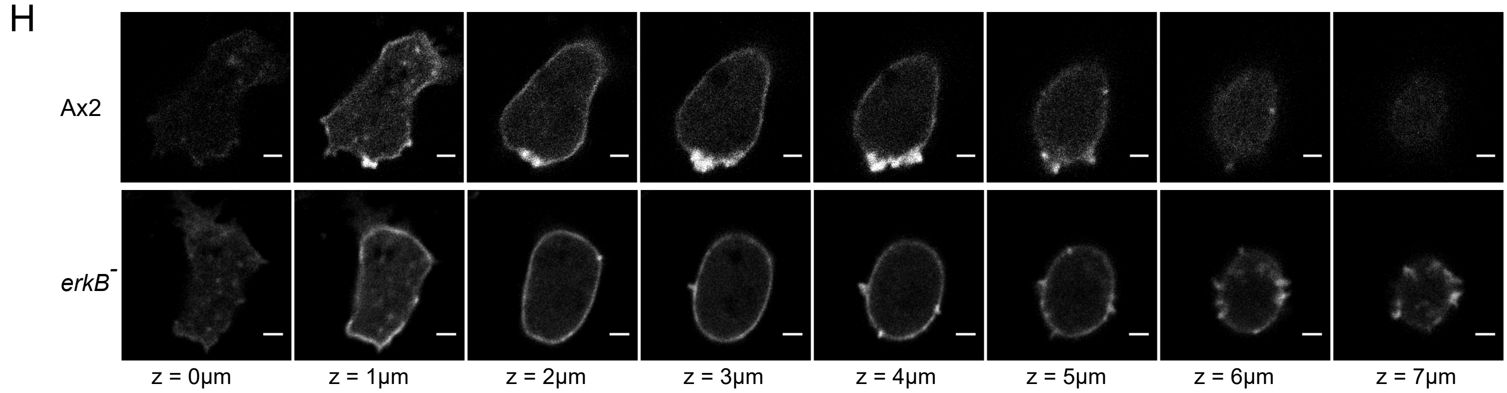

Figure S5. Chemotaxis and cytoskeletal phenotypes of $erkB^-$ mutants. Related to Figure 6.

(A) Individual cell tracks for undifferentiated wild-type (Ax2) and $erkB^-$ amoebae during chemotaxis under agarose containing 1 µM and 0.1 µM folate. N=60 cells. Scale in micrometres. See Figure 6F for chemotaxis parameters.

(B) Immunoblotting timecourse of ErkB phosphorylation following 50 µM folate stimulation of wild-type cells and of AK240 $erkB^-$ REMI mutant cells (hypomorph), showing residual ErkB activation in the AK240 insertional mutant cells.

(C) Individual cell tracks for undifferentiated wild-type (Ax3) and *erkB* hypomorph mutant amoebae during chemotaxis under agarose containing 10 µM folate. N=100 cells, ≥ 20 cells of three independent assays. Scale in micrometres. See Figure 6G for quantification of chemotaxis parameters.

(D) Scheme of under agarose chemotaxis cell mixing experiments. Chemotaxis in the under agarose assay results from amoebae degrading folate present in the agarose. This results in production of a self-generated chemoattractant gradient in which the cells chemotax as a wave. To test whether chemotaxis defects of $erkB^-$ cells in this assay are due to a defect in folate breakdown, wild-type and mutant amoebae expressing GFP or mCherry respectively were mixed in a 1:1 ratio. Both wild-type and $erkB^-$ cells were placed in a well cut in an agarose sheet containing 10 µM folate. Cells were able to move underneath the agarose layer (Boundary). Chemotaxis was filmed and cells tracked in the leading front of cells, in the main body of the cell wave, and in the region lagging behind the cell wave.

(E) Cell tracks of wild-type (green) and $erkB^-$ (red) cells in leading, wave and lagging regions. No $erkB^-$ cells were observed at the leading edge of the cell wave. N=60 cells, 20 cells from each of three independent assays. Scale in micrometres.

(F) Chemotaxis parameters of cells in different regions. In the main cell wave, where wild-type amoebae are able to chemotax, $erkB^-$ amoebae show defective speed, directionality and chemotactic index in the same gradient. Bar indicates mean of all cells. Error bars show SD. Statistical analysis were performed using Welch's t-tests, ****$p < 0.0001$, ***$p < 0.001$.

(G) Direct comparison of speed, chemotactic index, persistence and directness. Statistical analyses were performed using Welch's t-tests, ****$p < 0.0001$, ***$p < 0.001$.

(H) Images of F-actin localisation in wild-type and $erkB^-$ vegetative amoebae assayed by lifeact:mCherry fluorescence. Images are confocal z-planes at 1 µm intervals from the ventral cortex of cells upwards. F-actin puncta are observed within the ventral 2 µm of both wild-type and $erkB^-$ cells. Scale bar = 1µm

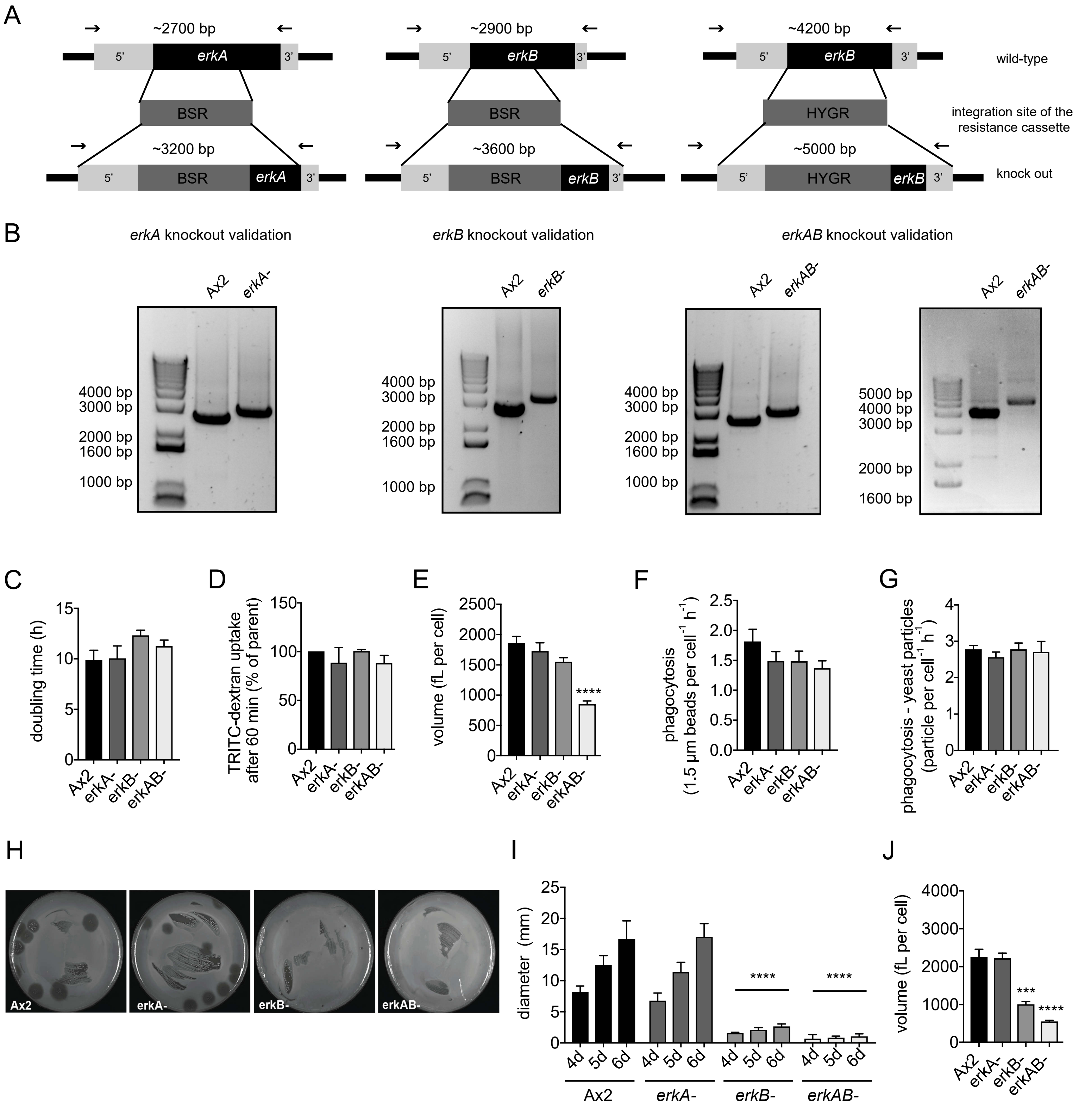
A
~2700 bp
5'
erkA
3'
~2900 bp
erkB
~4200 bp
wild-type
BSR
HYGR
integration site of the resistance cassette
~3200 bp
~3600 bp
~5000 bp
knock out
B
erkA knockout validation
erkB knockout validation
erkAB knockout validation
Ax2
erkA-
erkB-
erkAB-
5000 bp
4000 bp
3000 bp
2000 bp
1600 bp
1000 bp
C
doubling time (h)
D
TRITC-dextran uptake after 60 min (% of parent)
E
volume (fL per cell)
****
F
phagocytosis (1.5 µm beads per cell⁻¹ h⁻¹)
G
phagocytosis - yeast particles (particle per cell⁻¹ h⁻¹)
H
I
diameter (mm)
4d
5d
6d
J
***

Figure S6. Mutant validation and analysis of the physiological behavior of *erkA⁻*, *erkB⁻* and *erkAB⁻* cells. Related to Figure 3 and 6.

(A) Schematic overview of the strategy used to knock out *erkA* and *erkB*. The *erkA* gene was disrupted using a BS resistance cassette while two different constructs were created to interrupt the *erkB* gene locus, one using a BS and another one using a Hygromycin resistance cassette. Light grey bars indicate the cutoffs of the recombination arms used, dark grey bars show the resistance cassettes, while black bars indicate the gene coding sequence. Black lines represent regions down stream and up stream of the targeted gene, while arrows indicate primer binding regions used for the identification of knockout mutants. The site of insertion of the restriction cassette is indicated. The sizes of fragments expected in wild-type and knock out mutants is shown.

(B) PCRs confirming knockout mutants for *erkA⁻*, *erkB⁻* and *erkAB⁻* cell lines. The primer combinations indicated in (A) were used to confirm the accuracy of the three erk mutant strains.

(C) Axenic growth of *erkA⁻*, *erkB⁻* and *erkAB⁻* cells was measured in shaking suspension. Growth rates were compared. The doubling times of the mutant strains are shown. No significant differences were observed.Graphs show mean of three experiments. Error bars indicate the SEM.

(D) The ability to conduct bulk fluid uptake was assayed in same mutant strains. TRITC-dextran uptake was analysed after 60 min using flow cytometry. Results are plotted relative to Ax2. No fluid uptake defect was observed. Bar graphs show the mean of three independent measurements, error bars show the SEM.

(E) The cell volume of axenically grown mutant strains was measured. Cell volume was determined using an Eclipse flow cytometer. Bar graphs show the mean of three measurements. Significant decrease in cell volume was found for *erkB⁻* and *erkAB⁻* cells. While *erkB⁻* show just a mild decrease in cell volume, *erkAB⁻* are half the size of the parent strain Ax2. Statistical analyses were performed using a Welch's t-test (*$p<0.1$, ****$p<0.0001$). Error bars show the SEM.

(F) The phagocytosis of small particles was analysed in all mutant strains using 1.5 µm beads. Cells were incubated with fluorescent latex beads for 60 min, washed free of residual beads and uptake was analysed by flow cytometry. *erkA⁻*, *erkB⁻* and *erkAB⁻* cells showed a mild but non-significant defect in bead uptake. Plot shows mean bead uptake per cell per hour for five independent measurements. Statistics show a Welch's t-test (***$p<0.001$). Error bars show SEM. Values were compensated for cell size.

(G) Yeast particle uptake was performed using microscopy. Cells were fed for 60 min and quenched with trypan blue before analysis. 100 *Dictyostelium* cells were analysed per cell line and experiment. Bar graphs show the means of three experiments. Similarly to the 1.5 µm bead uptake assy, all knockout cell lines behave like the parent strain. Statistics were performed using a Welch's unpaired t-test (***$p<0.001$, ****$p<0.0001$). Error bars show the SEM. Values were compensated for cell size.

(H) Photographs of plaques of analysed mutant cell lines, illustrating the ability to grow on bacterial lawn. *erkB⁻* and *erkAB⁻* cells show a strong growth defect. Cells were grown in conjunction with *K. aerogenes* bacteria for 6 days before imaging.

(I) Quantification of bacterial growth assays on bacterial lawns. Parent and mutant strains were grown in conjunction with *K. aerogenes* bacteria. *Dictyostelium* cells were plated at high dilution to ensure single colonies were formed. The diameter of plaques was measured after 4d, 5d and 6d. At least 5 plaques were analysed per experiment. As shown in (H) *erkB⁻* and *erkB⁻* cells have severe growth defects on bacterial lawns, showing a 10-fold decreased plaque size. Bar graphs show the mean of the diameters from three experiments. Statistics were performed using a Welch's t-test (****$p<0.0001$). Error bars show the SEM.

(J) The cell volume of bacterial grown mutant cell strains was investigated. The volume was measured using an Eclipse flow cytometer. The mean of three independent measurements is shown as bar graphs. The *erkA⁻* cells have a similar cell volume as the parent strain while *erkB⁻* and *erkAB⁻* cells show a 2 fold to 4 fold reduced cell volume. The volume is plotted as bar graphs in fL per cell. The mean of five experiments is shown. Statistics were performed using a Welch's t-test (***$p<0.001$, ****$p<0.0001$). Error bars show the SEM.

A

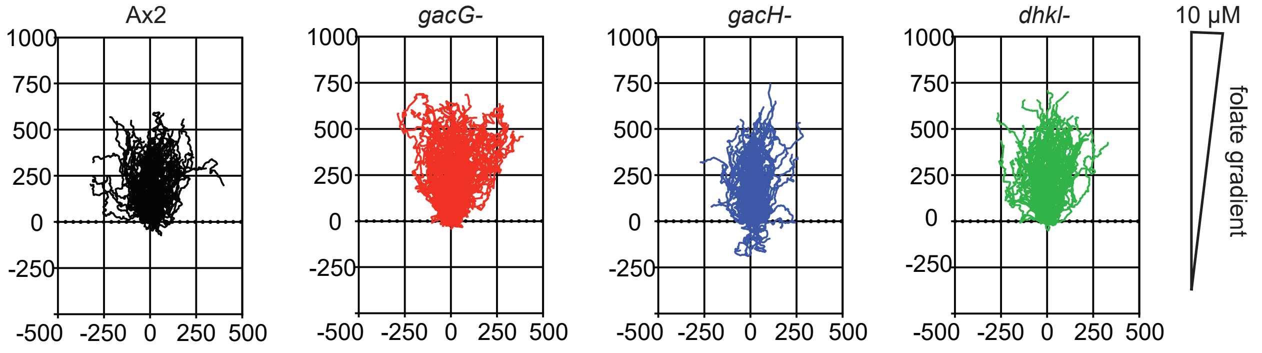

B

10 µM folate	Ax2	gacG-	gacH-	dhkl-
Chemotactic Index (a.u.)	0.487<br>SD ± 0.124	0.453 ns<br>SD ± 0.087	0.467 ns<br>SD ± 0.206	0.539 ns<br>SD ± 0.097
Speed (µm/min)	12.280<br>SD ± 2.008	20.140 ****<br>SD ± 5.102	11.460 ns<br>SD ± 2.073	15.060 ns<br>SD ± 2.452
Persistence (a.u.)	0.553<br>SD ± 0.033	0.545 ns<br>SD ± 0.047	0.564 ns<br>SD ± 0.054	0.570 ns<br>SD ± 0.038
Directness (a.u.)	0.515<br>SD ± 0.114	0.471 ns<br>SD ± 0.085	0.505 ns<br>SD ± 0.168	0.553 ns<br>SD ± 0.090

C

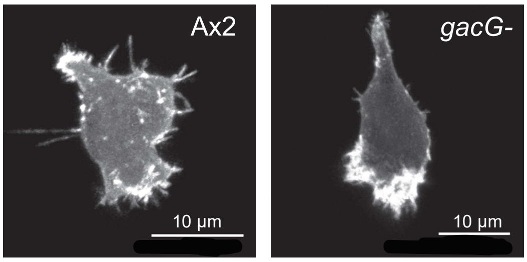

D

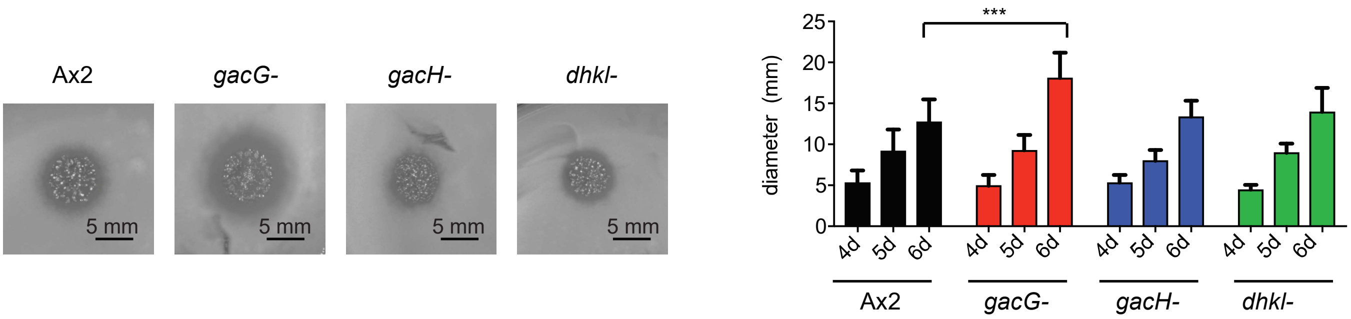

E

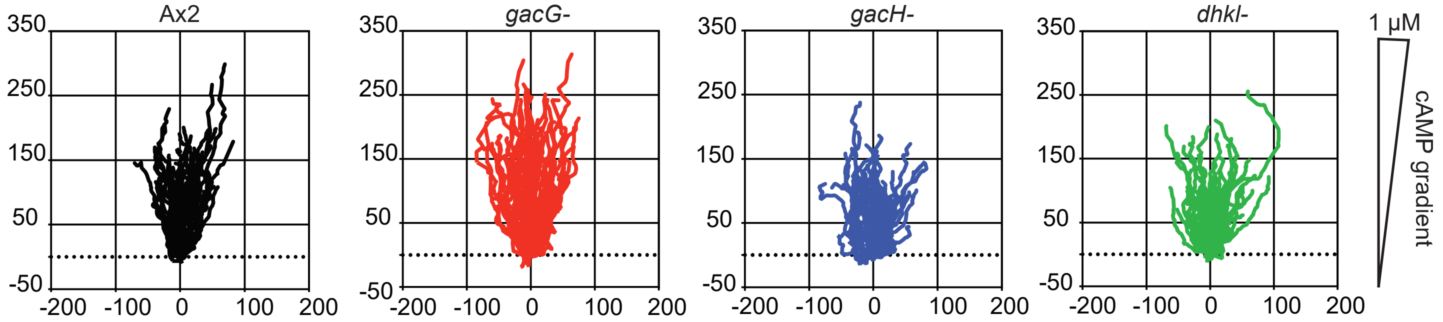

F

1 µM cAMP	Ax2	gacG-	gacH-	dhkl-
Chemotactic Index (a.u.)	0.805<br>SD ± 0.046	0.794 ns<br>SD ± 0.091	0.675 ***<br>SD ± 0.1069	0.728 *<br>SD ± 0.105
Speed (µm/min)	13.36<br>SD ± 3.752	17.76 ***<br>SD ± 3.864	12.44 ns<br>SD ± 3.601	12.34 ns<br>SD ± 4.224
Persistence (a.u)	0.854<br>SD ± 0.093	0.868 ns<br>SD ± 0.084	0.816 ns<br>SD ± 0.119	0.830 ns<br>SD ± 0.117
Directness (a.u.)	0.8216<br>SD ± 0.044	0.805 ns<br>SD ± 0.090	0.6945 **<br>SD ± 0.108	0.7588 *<br>SD ± 0.089

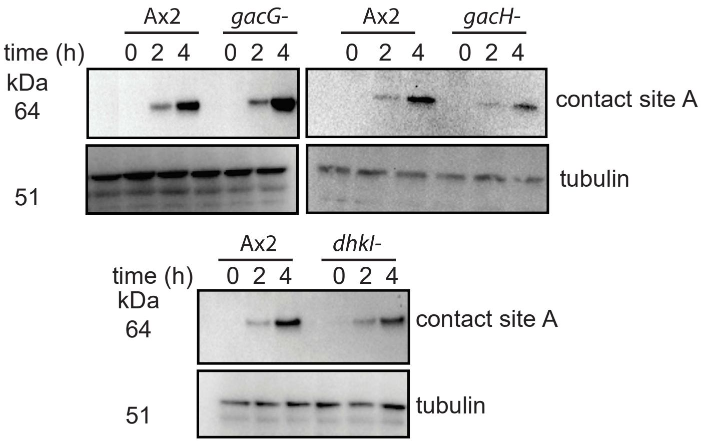

G

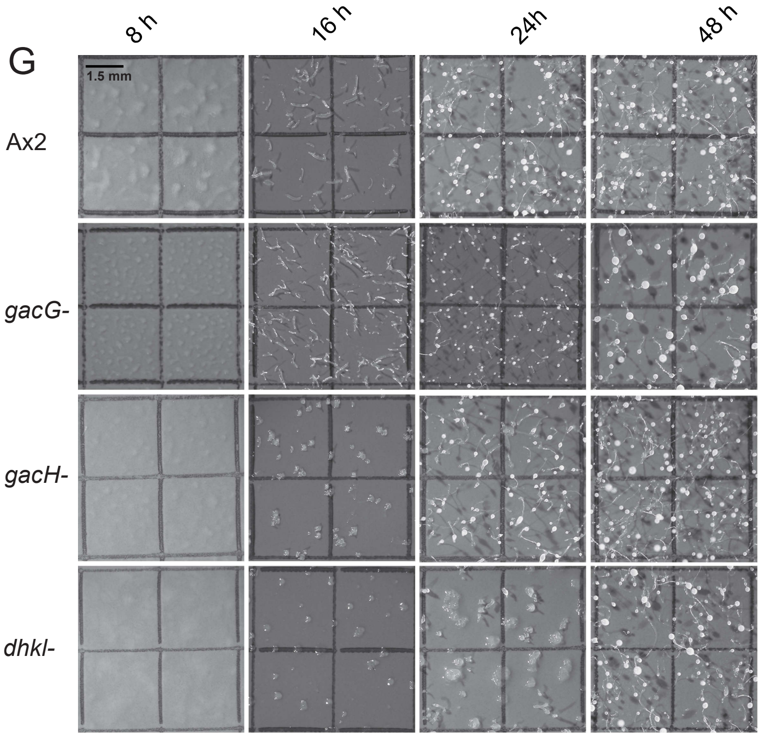

Figure S7. Core proteins show chemotaxis or motility phenotypes. Related to Table 1.

(A) Ax2, *gacG*⁻, *gacH*⁻ and *dhkI*⁻ cells were allowed to chemotax to folate under agarose and examined by microscopy. The tracks of 100 cells per cell line were plotted (The triangle indicates the direction of the folate gradient; scale in micrometres. ≥ 20 cells of three independent assays were analysed).

(B) Quantification of chemotactic index, speed, persistence and directness of wild-type Ax2 and knockout cells plotted in (A). Only *gacG*⁻ cells show an increase in cell speed. The chemotactic index and directionality are wild-type as in all assayed cell lines. Presented is the mean ± SD. Statistical analyses show Welch's t-tests, ****p < 0.0001, ns >0.1.

(C) Comparison of undifferentiated wild-type Ax2 and *gacG*⁻ amoebae expressing the F-actin marker lifeact::mCherry. Cells were grown in bacterial suspension and washed free of bacteria before imaging. Shown are Z-projections of confocal sections. *gacG*⁻ cells are more strongly polarized than Ax2 cells. Scale bar = 10 µm.

(D) Representative images and quantification of the plaque diameters of Ax2, *gacG*⁻, *gacH*⁻ and *dhkI*⁻ cells. *Dictyostelium* amoebae were grown in conjunction with *K. aerogenes* bacteria on SM agar plates using high dilutitions to ensure the formation of single plaques. Diameters were quantified after 4d, 5d and 6d of growth. Per time point and cell line at least 5 single plaques were measured (N=3). Images display a single representative plaque for each cell line, photographed on day 6. *gacG*⁻ cells exhibit an increase in plaque size after day 6. Graphs show the mean ± SD. Statistical analysis was performed using a Welch's t-test, ***p < 0.001.

(E) Ax2, *gacG*⁻, *gacH*⁻ and *dhkI*⁻ cells were allowed to chemotax to cAMP in an Insall chamber examined by microscopy. The tracks of 60 cells per cell line were plotted to illustrate their chemotactic behavior. Triangle indicates direction of the cAMP gradient; scale in micrometres. 20 cells of three independent assays were analysed.

(F) The chemotactic index, speed, persistence and directness of wild-type Ax2 and knockout cells plotted in (E) were quantified. Displayed is the mean ± SD. Statistical analyses show Welch's unpaired t-tests, ****p < 0.00001, ns >0.1. Western blot analysis was used to check if developmental genes are expressed at correct time points. Samples of cAMP pulsed cells were taken at the indicated time points. The contact site A antibody confirms a proper developemental timing in all knockout mutants. Tubulin was used as loading control.

(G) Developmental time course of all four strains. Cells were developed on gridded filters. Images were captured with a low magnification microscope every 8 h with a final image after 48 h confirming all cell lines produce mature fruiting bodies. Scale bar = 1.5 mm.

Protein	Role	10s cAMP	45s cAMP	360s cAMP
cAR1	cAMP receptor	*S299**<br>**S302***<br>**S303***<br>*S324**<br>*S325**	*S324**	*S299**<br>*S325**
Gα2	Gα subunit			*S113**
RckA	RGS protein		**S83**	**S349**<br>**S351**
GefR	GEF for RasG	**S434**<br>**T860**	**S433**<br>**S468**<br>**S846**<br>**S858**<br>**T860**	**S310**
Sca1	Ras scaffold protein	**S359***<br>**S923**	**T357**<br>**S359***<br>**S372**<br>**S401**<br>**S920**<br>**S921**<br>**S923**<br>**S946**	**S355**<br>**S359***<br>**S920**<br>**S946**
RipA	TORC2 component	**S301**	**T300**<br>**S301**	**S301**<br>*S642*
PikI	PIP5K		**T262***<br>*T338*<br>*S585*	
SHAPS	PKB substrate	**T395**	**S258**	**S258**
PikA	PI3K	**S199**	**S199**	**S199**
PikB	PI3K	**S518**	**S518**<br>**S536**	**S518**<br>**S536**<br>**S542**
GbpC	cGMP binding protein	**S1566**<br>**S2322**	**S1566**<br>**S2262**<br>**T2312**<br>**S2322**<br>**T2329**	**S2322**
DocA	DOCK RacGEF		**S12**<br>**S480**<br>**S481**<br>**S486**<br>**S756**<br>**S762**	**T15**
ElmoE	Gβγ effector	*S994*	*S994*	*S925*<br>**S1675**
MyoE	Myosin I	**S334**	**S334**	
MyoG	MyTH/FERM myosin	**S1593**	**S1504**<br>**S1593**	**T1387**<br>**S1504**
RegA	PDE/response regulator		**S413**	**S413**
Tsunami	Protein kinase		**S579**	**S575**<br>**S579**

Table S1. cAMP responsive phosphorylation sites detected on proteins with known roles in chemotactic signal transduction. Related to Figure 1.

Selected known chemotaxis proteins observed to be phosphorylated in cAMP SILAC experiments are shown. Individual phosphorylation sites are listed. Sites with increased phosphorylation at a given timepoint are shown in bold. Sites with decreased phosphorylation at a given timepoint are shown in italics. Phosphorylation sites previously described in the literature are marked with *.

Site	Protein	Wild type ratio (log$_2$)	*erkB*$^-$ KO ratio (log$_2$)	Sequence window	Protein detected in *erkB*$^-$ KO
**Sites upregulated in wild type and not detected in *erkB*$^-$ KO**					
T1178	DhkI-1	5.7	ND	YSNLISTPRGGVG	🔴
S297	GacQ	4.6	ND	SSKNPISPRSPIG	🔴
T550	DDB_G0270072	3.9	ND	KHNNPITPRQRLN	🔴
T233	ForA	3.7	ND	GSLSPVTPRVDDG	🟢
S1093	DDB_G0288915	3.7	ND	TTTNPVSPRFVST	🟢
S293	GefM	3.6	ND	VKPPPPSPRFSTN	🟢
T254	MhkD	3.6	ND	LYIVPTTPRPSKS	🟢
S530	SepA	3.4	ND	NKKLPLSPRQPSS	🟢
S596	DDB_G0272006	3.2	ND	SGSPSNSPRLFTK	🟢
T405	DDB_G0268078	3.1	ND	NQKLDSTPRLSPR	🟢
T963	DDB_G0292746	3.0	ND	DSSIPLTPR____	🔴
T550	GacHH	3.0	ND	ASPSPLTPRSIAR	🟢
S536	PikB	3.0	ND	ESDISSSPRSIGS	🟢
S469	GacG	2.9	ND	LNIIGISPRGTNR	🟢
S1070	DDB_G0288915	2.8	ND	KSVNPSSPRYTST	🟢
S335	DDB_G0287765	2.6	ND	NTPLRTSGNNYKN	🔴
T291	DDB_G0269608	2.5	ND	PNSGISTPRAINR	🔴
S577	DDB_G0282105	2.4	ND	QSKMTISPRDKDR	🟢
S1504	MyoG	2.4	ND	ITGAPKSPRNSSE	🟢
S447	GacG	2.3	ND	SSSSTSSPRVNAP	🟢
T58	GacY	2.0	ND	APQRKVTFGSRVR	🔴
T60	DDB_G0273377	1.9	ND	TTPTSTTPRSKSS	🟢
S994	DDB_G0278311	1.8	ND	DEKKSSSRFFGKS	🟢
T984	DDB_G0268348	1.8	ND	SSTNTNTPRTIVL	🔴
S430	GacG	1.7	ND	SSSTSSSPRNNGN	🟢
S2141	DDB_G0292230	1.6	ND	GLENSGSIAVGRG	🟢
S752	DDB_G0283821	1.5	ND	SRKISNSDQFTPN	🟢
S1482	DhkI-1	1.4	ND	TSPPISSPRSNNN	🔴
S2846	DDB_G0285063	1.4	ND	SFNRIPSPRRILK	🟢
T573	GacF	1.4	ND	RPRKGSTVQYLNR	🟢
S30	Kif9	1.4	ND	LRSRSNSSPSTSS	🟢

S31	DDB_G0292322	1.3	ND	NKSRNNSRESGLS	●
S149	DDB_G0291205	1.3	ND	IQGPTISPRTLSI	●
T431	RapGAP3	1.3	ND	ILPRKSTIIGPNG	●
T262	DDB_G0267588	1.1	ND	NRVRLNTSQRLKM	●
S228	ForA	1.0	ND	SNASLGSLSPVTP	●
**Sites upregulated in wild type with no response in *erkB*$^-$ KO**					
T85	DstA	4.8	0.0	GRSNNLTPRTNQL	●
S390	DDB_G0281657	2.9	0.1	LSFLPNSPRNPIS	●
S833	DDB_G0278995	2.6	0.2	VRFSYLSPRGGNQ	●
S289	DDB_G0272638	2.1	0.2	LINFQRSPRGTLT	●
S251	KeaA	1.9	-0.1	NGSGQFSPRIHPS	●
S325	EppA	1.5	0.1	RNPFGGSPRNYDN	●
S367	GefS	1.5	-0.2	NIKTGSSSDSFLK	●
S1452	Roco9	1.4	-0.2	KLPSPISPRQSFI	●
S960	AbcC8	1.2	0.1	NKKLNNSGSGVSL	●
**Sites upregulated in both wild type and *erkB*$^-$ KO**					
S618	PikG	5.1	3.8	TNSKSSSSNNLFK	●
T627	GefL	5.0	3.1	LQQTVSTPRQQSF	●
S363	DDB_G0281653	2.2	3.2	NRYRSNSGRDNLP	●
T860	GefR	1.9	2.5	KKERSFTIGLVSG	●
S301	RipA	1.8	2.0	VTFTGTSVKLNSK	●
S572	GacF	1.7	2.1	TRPRKGSTVQYLN	●
S361	DDB_G0281653	1.6	3.0	ALNRYRSNSGRDN	●
S2322	GbpC	1.5	2.4	DGARSGSISYLGR	●
T50	DDB_G0275317	1.2	2.1	QRKRGATVIGTRE	●
S110	DDB_G0279731	1.1	1.7	GRSASLSINNRDA	●
T135	DDB_G0275345	1.1	2.0	DSFRRATINTGSI	●
S847	DDB_G0286003	1.1	1.6	QRSRSNSNSSTAS	●

Table S2. Comparison of phosphorylation changes in wild type and *erkB*⁻ knockout cells following 45s folate treatment. Related to Figure 4

Phosphorylation sites with increased phosphorylation after 45s folate treatment in wild type cells are listed, grouped by phosphorylation response in wild type and *erkB*⁻ experiments.

Sequence windows show amino acid sequence context for each phosphorylation site and are colour coded by kinase consensus motif; red for p[S/T]PR sites, yellow for Akt/PKB sites (RxRxxp[S/T]).

ND = site not detected in *erkB*⁻ experiment. Green dots indicate that other peptides mapping to the same protein were observed in the *erkB*⁻ experiment, confirming expression of the protein in *erkB*⁻ cells. Red dots indicate that no other peptides mapping to the same protein were observed in the *erkB*⁻ experiment. In most cases where a phosphorylation site was not detected in *erkB*⁻ cells, expression of the protein was detected in the same sample.
